# Asymmetric Synthesis of *N*‐Substituted α‐Amino Esters from α‐Ketoesters via Imine Reductase‐Catalyzed Reductive Amination

**DOI:** 10.1002/anie.202016589

**Published:** 2021-03-09

**Authors:** Peiyuan Yao, James R. Marshall, Zefei Xu, Jesmine Lim, Simon J. Charnock, Dunming Zhu, Nicholas J. Turner

**Affiliations:** ^1^ Department of Chemistry University of Manchester Manchester Institute of Biotechnology 131 Princess Street Manchester M1 7DN UK; ^2^ National Technology Innovation Center of Synthetic Biology National Engineering Laboratory for Industrial Enzymes and Tianjin Engineering Research Center of Biocatalytic Technology Tianjin Institute of Industrial Biotechnology Chinese Academy of Sciences 32 Xi Qi Dao, Tianjin Airport Economic Area Tianjin 300308 P.R. China; ^3^ Prozomix Ltd Building 4, West End Ind. Estate Haltwhistle NE49 9HA UK

**Keywords:** biocatalysis, chiral amines, imine reductase, reductive amination, α-amino acid

## Abstract

*N*‐Substituted α‐amino esters are widely used as chiral intermediates in a range of pharmaceuticals. Here we report the enantioselective biocatalyic synthesis of *N*‐substituted α‐amino esters through the direct reductive coupling of α‐ketoesters and amines employing sequence diverse metagenomic imine reductases (IREDs). Both enantiomers of *N*‐substituted α‐amino esters were obtained with high conversion and excellent enantioselectivity under mild reaction conditions. In addition >20 different preparative scale transformations were performed highlighting the scalability of this system.

## Graphical abstract

The asymmetric synthesis of *N*‐substituted α‐amino esters has been achieved, in high yields and excellent enantioselectivities, employing sequence diverse metagenomic imine reductases (IREDs).

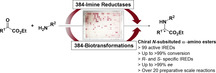

*N*‐Substituted α‐amino acids and their derivatives have attracted increasing attention in the pharmaceutical and fine chemical industries in recent years, forming the key scaffolds in a number of bioactive molecules (Figure 1).^[1]^ For example, *N*‐methylated analogues of peptides and peptidomimetics can improve the pharmacokinetic properties of such molecules, including metabolic stability, membrane permeability, and oral bioavailability.^[2]^ Although tremendous efforts have been devoted to the preparation of *N*‐alkyl‐α‐amino acids,[^1a^, ^3^] including bio‐inspired asymmetric reductive aminations,^[4]^ there are a number of limitations to these synthetic methods. The *N*‐alkylation process often requires genotoxic alkylating agents, such as alkyl halides,^[3a]^ and can be impractical on large scale due to difficulty in removing specific protecting groups.^[5]^ Biomimetic routes also have their constraints due to the need to preform the imine and the employment of transition metals such as ruthenium.[^4^, ^6^]

**Figure 1:**
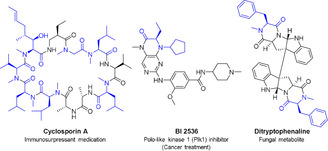
Representative *N*‐substituted α‐amino acid derivatives with biological activities where the *N*‐substituted α‐amino acid scaffolds are highlighted in blue.

As a more sustainable and alternative approach to chemical methods, biocatalysis has become an attractive option in preparing chiral *N*‐substituted α‐amino acids or their derivatives.^[1]^ Current approaches include *N*‐methylation of amino acids and peptides by *N*‐methyltransferases,^[7]^ synthesis of *N*‐arylated aspartic acids catalyzed by ethylenediamine‐*N*,*N*′‐disuccinic acid lyase,^[8]^ and reductive amination of α‐ketoacids by NAD(P)H‐dependent oxidoreductases including opine dehydrogenases (OpDHs), ketimine reductases and *N*‐methyl amino acid dehydrogenases,^[9]^ and engineered heme‐dependent proteins.^[10]^ However, the current enzymatic toolbox for the synthesis of *N*‐substituted α‐amino acids has several limitations including strict stereoselectivity for formation of the l‐(*S*)‐enantiomer. Many enzyme families have narrow substrate specificities, with respect to either the α‐ketoacid or amine partner, with high activities limited to simple primary amines such as methylamine. Furthermore, with the exception of a single patent reporting reductive aminations employing OpDHs, limited information is available regarding stereoselectivities and activities.^[11]^ Thus, methods for the asymmetric synthesis of *N*‐substituted α‐amino acids, or their derivatives, to access both enantiomeric series across a broad range of substrates, remain a significant challenge.

Imine reductases (IREDs) and reductive aminases (RedAms) belong to a family of NAD(P)H‐dependent oxidoreductases which have been utilised in the synthesis of chiral amines employing both reductive amination and cyclic imine reduction reactions.^[12]^ Reductive amination, employing wild‐type IRED biocatalysts, has helped to define the substrate scope across both carbonyl acceptors and amine partners (primary, and secondary amines).^[13]^ Recently, a large metagenomic (384 enzymes) IRED panel was generated and applied to the reductive coupling of β‐ketoesters to generate enantiocomplementary *N*‐substituted β‐amino esters on preparative scales.^[14]^ Furthermore, the synthetic utility of this enzyme class was recently highlighted on a kilogram scale using an engineered IRED for the synthesis of a lysine‐specific demethylase‐1 (LSD1) inhibitor, GSK2879552.^[13a]^

We therefore sought to evaluate the wild‐type metagenomic IRED panel^[14]^ for the reductive amination of aliphatic and aromatic α‐ketoesters, using various amine partners, to assess both the level of conversion and enantioselectivities, in order to extend the IRED catalysed reductive amination scope to this class of compounds.

Previous reports had demonstrated the ability of metagenomic IREDs and RedAms to accept γ‐ and β‐ketoesters for reductive aminations.[^13e^, ^14^] However, with α‐ketoacids, specifically pyruvic acid, low activities were observed towards this substrate using the reductive aminase from *Aspergillus oryzae* (*Asp*RedAm).^[13e]^ A genetically engineered *Corynebacterium glutamicum* strain, expressing an imine reducing enzyme of a different oxidoreductase family to the metagenomic IREDs, belonging to Delta(1)‐pyrroline‐2‐carboxylate/Delta(1)‐piperideine‐2‐carboxylate reductase (*DpkA*) was shown to transform pyruvate to *N*‐methyl‐l‐alanine.^[15]^ Hence exploring additional IRED and RedAm sequence space was of interest to see if we could obtain greater activities with α‐ketoester substrates.

The panel of 384 IREDs (cell‐free extracts) was initially screened for the reductive amination of model substrate ethyl 2‐oxo‐4‐phenylbutyrate (**1**, 25 mm) with propargylamine (**a**, 50 mm) in a 100 μL reaction volume. This initial screen revealed that 99 out of the 384 different IREDs (as shown in Supplementary Table S3) were found to catalyse the desired transformation. Further analysis revealed that 78 of these IREDs were *R*‐selective (of which 35 IREDs exhibited excellent stereoselectivity with *ee*>99 %), while 20 were found to be *S*‐selective where only pIR‐338 showed both excellent conversion and selectivity, and one IRED generated racemic **1 a**. The top 5 *S*‐selective and top 7 *R*‐selective enzymes, with relative activities for the generation of **1 a**, are shown in Table 1. These 12 enzymes were subsequently selected for further reductive amination reactions with a broader range of α‐ketoesters.

**Table 1:** Conversion and enantioselectivity of the top 12 selected IREDs out of 384 enzymes towards reductive amination between ethyl 2‐oxo‐4‐phenylbutyrate (**1**) and propargylamine (**a**).^[a]^ (*S*)‐**1 a** given in green and (*R*)‐**1 a** given in blue. 
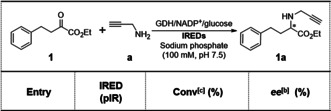

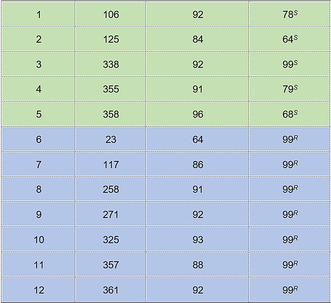

[a] Reaction conditions: 25 mm ethyl 2‐oxo‐4‐phenylbutyrate (**1**), propargylamine (**a**, 50 mm), 5 mg mL^−1^ lysate of *E. coli* expressing IRED, 6 U mL^−1^ CDX‐901 glucose dehydrogenase (GDH), 0.4 mm NADP^+^, 62.5 mm glucose, 10 % (v/v) DMSO, sodium phosphate buffer (100 mm, pH 7.5), 100 μL reaction volume, 30 °C, 200 rpm, 20 h. [b] Enantiomeric excess (*ee*) was determined by chiral HPLC. [c] Conversion into product was determined by GC.

Initially the 12 IREDs were evaluated across a variety of aryl and alkyl α‐ketoesters (**2**–**11**) ([S]=50 mm) with propargylamine (**a**) selected as the amine donor ([S]=100 mm) on an analytical scale. All ketoester substrates except **10** and **11** were transformed to the corresponding *N*‐propargyl amino esters with moderate to high conversion, high stereoselectivity, and with complementary enantiopreference (Figure 2 and Supplementary Table S4); larger substituents, such as benzyl and pentyl groups, were tolerated well. Interestingly, the stereoselectivities of enzymes such as pIR‐117 and pIR‐258 were inverted when challenged with different α‐ketoesters. For example, both IREDs generated (*R*)‐**2 a** with a benzyl substituent at the β‐position with >99 % *ee*. However, when this substituent was modified to a methyl group, both enzymes generated (*S*)‐**3 a** with 97 % *ee* for pIR‐117 and 94 % *ee* for pIR‐258. This inversion of stereoselectivity is not uncommon within this enzyme family and has been previously observed.^[16]^

**Figure 2:**
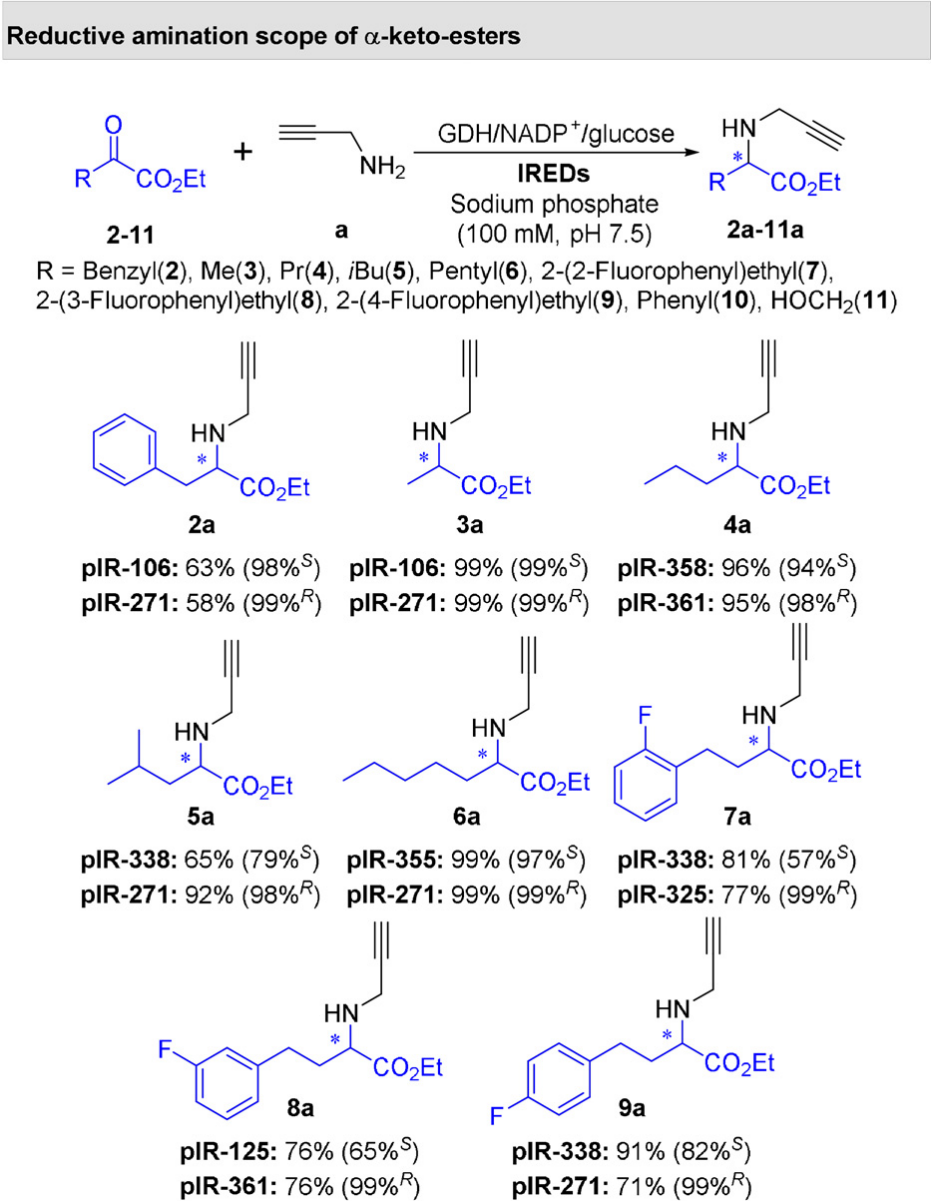
Reductive amination scope of α‐ketoesters with varying substituents at the β‐position. % Conversion into product and enantioselectivity (% *ee*) of 12 selected IREDs towards α‐ketoesters (**2**–**9**) and propargylamine (a) where the top IREDs to generate *S* and *R* enantiomers are shown with an extended table show in Supplementary Table S4. No IREDs showed activity towards **10** and **11**.

The scope of the selected 12 IREDs was further evaluated with a variety of amine partners (Table 2) where analytical scale biotransformations with **1** (50 mm) and amines **a**, **d**, **e**, and **g** (100 mm) and amines **b**, **c**, **f** (500 mm) were performed. Functionalised amines were selected including propargylamine (**a**), allylamine (**d**) and 4‐methylbenzylamine (**g**), as well as linear amines (propylamine (**c**)) and cyclic amines (cyclopropylamine (**e**)). As highlighted in Table 2, pIR‐23, pIR‐271, pIR‐325, and pIR‐338 exhibited excellent stereoselectivity towards **a**–**e**, whilst again the enantioselectivities of some IREDs varied depending on the amine partner presented. The stereoselectivity of pIR‐355 was inverted to afford the *R*‐enantiomers with amines methylamine (**b**) and allylamine (**d**) to generate (*R*)‐**1 b** and (*R*)‐**1 d** respectively. Only pIR‐23 showed activity towards **1** and **g** with 82 % conversion and 99 % *ee* (*R*), but no activity was observed for cyclopentylamine (**f**).

**Table 2:** Amine scope of reductive aminations with ethyl 2‐Oxo‐4‐phenylbutyrate. Conversion and enantioselectivity of selected IREDs towards Ethyl 2‐Oxo‐4‐phenylbutyrate (**1**) and different Amines (**a**–**g**). Highlighted in green is the top performing enzyme (both selectivity and conversion) for the given (*S*)‐product and blue highlights the top performing enzyme for the given (*R*)‐product.^[a]^ 
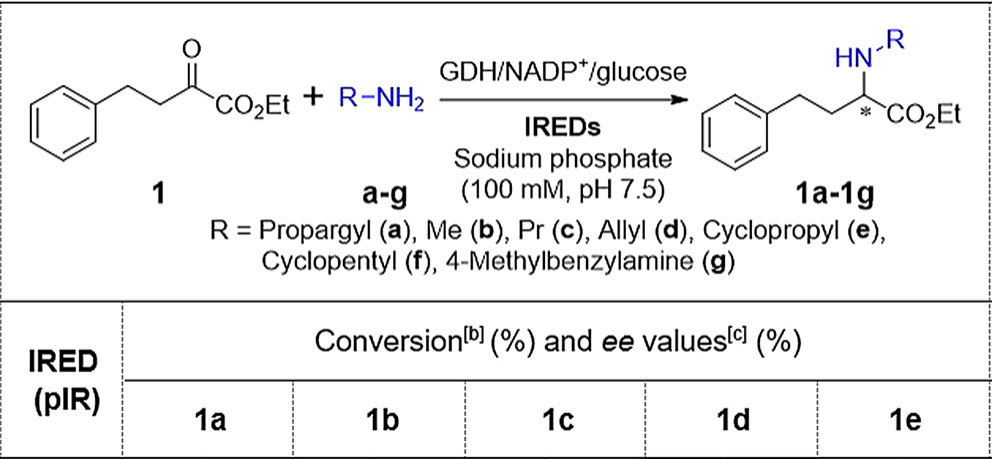

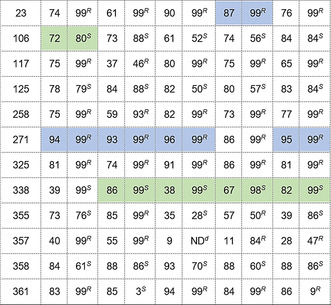

[a] Reaction conditions: 50 mm ethyl 2‐oxo‐4‐phenylbutyrate (**1**), amine (100 mm for **a**, **d**, **e**, and **g**, 500 mm for **b**, **c**, and **f**), 50 mg mL^−1^ *E. coli* whole cells expressing IRED, 6 U mL^−1^ CDX‐901 GDH, 0.4 mm NADP^+^, 125 mm glucose, 10 % (v/v) DMSO, sodium phosphate buffer (100 mm, pH 7.5), 500 μL reaction volume, 30 °C, 200 rpm, 20 h. [b] Conversion into product was determined by GC. [c] Enantiomeric excess (*ee*) was determined by chiral HPLC. [d] Not determined owing to low conversion for **1** and **f**, only pIR‐23 showed activity towards **1** and **g**.

To demonstrate the synthetic practicality of this biocatalytic approach, a series of preparative scale reactions was performed on a 2.5 mmol scale in a total reaction volume of 50 mL (Figure 3). For all of these preparative reactions, good to excellent conversions were observed (53–99 %), and the products were isolated as their HCl salts in moderate to high yields (27–80 %) with excellent *ee* values for *R*‐selective IREDs (98–99 %) and good to excellent *ee* values for *S*‐selective IREDs (26–99 %). With allylamine (**d**) as the amine partner, employing metagenomic IRED pIR‐271, (*R*)‐**1 d** was synthesised in 58 % isolated yield with excellent enantioselectivity (>99 %), with this enzyme also being employed on preparative scale to ascertain 13 out of a possible 14 (*R*)‐products (Figure 3). To highlight the robustness of these enzymes towards the preparation of **1 a**, we obtained space time yields up to 6.6 g L^−1^ d^−1^ with pIR‐271. Employing purified IR‐338 for the preparation of **1 a** we obtained total turnover numbers (TTN) of 3500 with a turnover frequency (TOF) of 24 min^−1^. Conversion to **1 a** was monitored over 24 hours with pIR‐271 (Figure S96), where nearly full conversion was reached after 2.5 hours of reaction time. Owing to the competing ketone reduction catalysed by endogenous *E. coli* ketoreductase activity, lower isolated yields of (*S*)‐**1 c**, (*S*)‐**1 d**, (*R*)‐**2 a**, and (*S*)‐**2 a** were obtained.^[17]^ This was further investigated with purified pIR‐338 for the preparation of **1 a** as shown in Supporting Information Table S5. The origin of this ketoreductase activity appears to be either directly the CDX‐901, or the lysate from which the GDH is formulated,^[18]^ as purified pIR‐338 demonstrated no ketoreductase acitvity.

**Figure 3:**
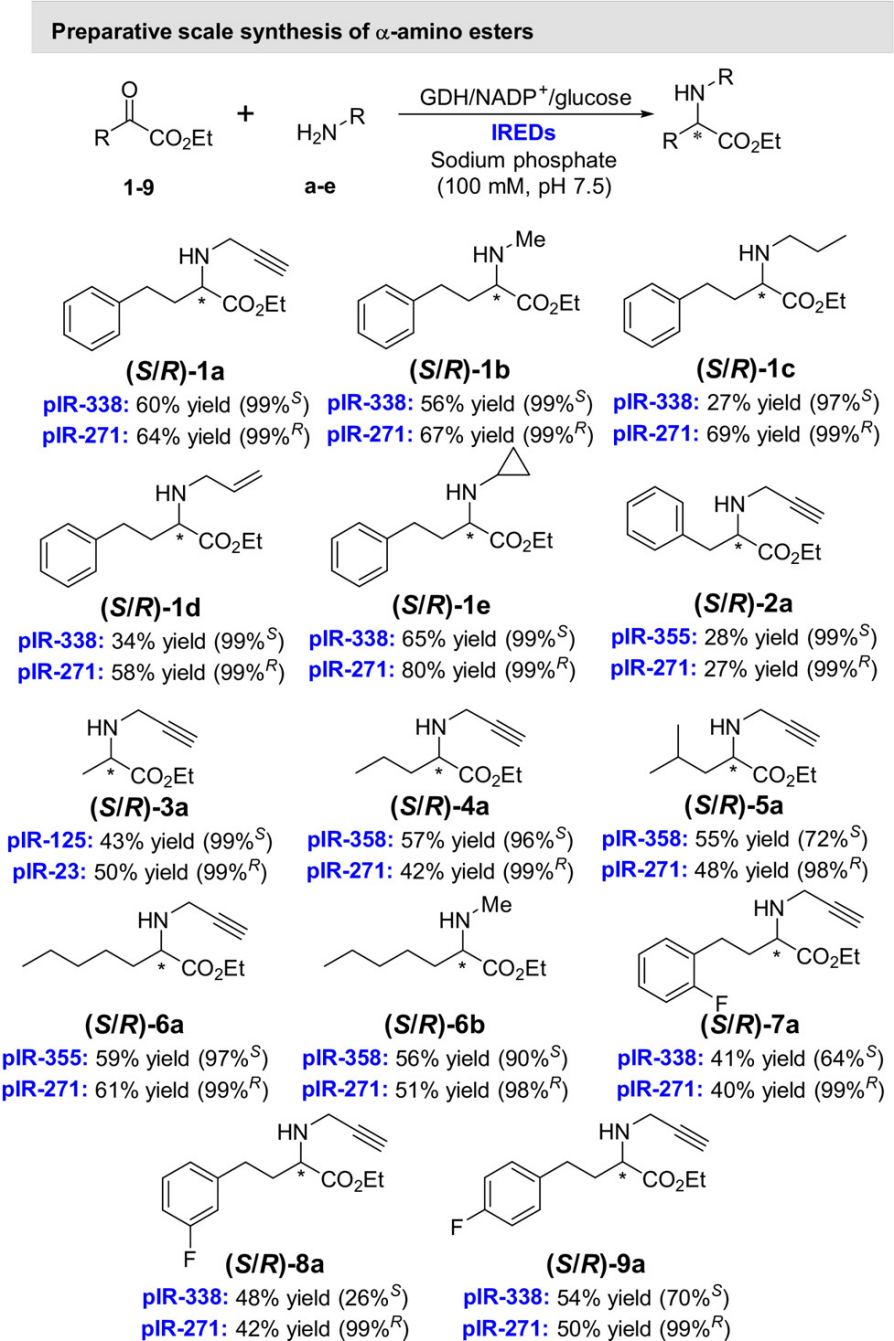
Preparative scale reductive aminations of α‐ketoesters with different amines to generate *N*‐substituted α‐amino esters. Each product isolated as the HCl salt with yields for each given and *ee* in brackets. Conversions into product were determined by GC analysis and *ee* values were determined by chiral GC analysis and HPLC. Full figure with conversion data inlcuded is given in Supplementary Figure S2.

In summary, we have developed a highly efficient biocatalytic strategy for the synthesis of *N*‐substituted amino esters from α‐ketoesters and amines catalysed by IREDs. This approach offers efficient access to various enantiomerically pure *N*‐substituted amino esters from aryl and alkyl substituted α‐ketoesters with exquisite and complementary enantioselectivities, addressing problems identified in previous chemocatalytic and biocatalytic approaches to attain these compounds. The synthetic utility and scalability of this system was highlighted through 28 preparative scale transformations. This study continues to emphasise the value and applicability of metagenomic imine reductases in the synthesis of high‐value chiral amines.

## Conflict of interest

The authors declare no conflict of interest.

## Supporting information

As a service to our authors and readers, this journal provides supporting information supplied by the authors. Such materials are peer reviewed and may be re‐organized for online delivery, but are not copy‐edited or typeset. Technical support issues arising from supporting information (other than missing files) should be addressed to the authors.

Supplementary
