# Supplementary material for: Asymmetric Synthesis of N‐Substituted α‐Amino Esters from α‐Ketoesters via Imine Reductase‐Catalyzed Reductive Amination
Source: Angew Chem Int Ed Engl. 2021 Mar 9;60(16):8717–21. doi: 10.1002/anie.202016589 (PMC8048798; doi:10.1002/anie.202016589)
Supplement: Supplementary file 1 — Supplementary [file ANIE-60-8717-s001.pdf]

Angewandte Chemie
Eine Zeitschrift der Gesellschaft Deutscher Chemiker

Supporting Information

Asymmetric Synthesis of *N*-Substituted α-Amino Esters from α-Ketoesters via Imine Reductase-Catalyzed Reductive Amination

Peiyuan Yao[+], James R. Marshall[+], Zefei Xu, Jesmine Lim, Simon J. Charnock, Dunming Zhu, and Nicholas J. Turner**

anie_202016589_sm_miscellaneous_information.pdf

Table of Contents

Experimental Procedures 3

Supporting Tables 17

Supporting Figures 19

References 70

1. Experimental Procedures

1.1. Materials

Commercially available chemicals and reagents were purchased from Sigma-Aldrich (Poole, Dorset, UK), Alfa Aesar (Karlsruhe, Germany), Fluorochem (Hadfield, Derbyshire, UK) and Acros Organics (Geel, Belgium) unless stated otherwise. Isopropyl β-D-1-thiogalactopyranoside (IPTG), kanamycin, Terrific broth (TB) and LB (Luria-Bertani) Agar Miller was purchased from Formedium (Hunstanton, England). α-Ketoesters **1**, **3** and **10** was purchased from Sigma-Aldrich (Poole, Dorset, UK). α-Ketoesters **2**, **4**, **5**, **6**, **7**, **8** and **9** were prepared from the diethyl oxalate by the addition of corresponding Grignard reagents following a procedure previously described.[1] Ethyl hydroxypyruvate was prepared from ethyl β-bromopyruvate as described in the reference.[2] Racemic **1a**, **1b**, **1c**, **1d**, **1e** and **1g** were synthesized from ethyl 2-oxo-4-phenylbutyrate (**1**) and corresponding amines following a similar procedure previously described.[3] Spectra of ^{1}H and ^{13}C NMR were recorded on a Bruker Avance 400 instrument (400 MHz for ^{1}H and 100 MHz for ^{13}C) or Bruker Advance 500 instrument (500 MHz for ^{1}H and 125 MHz for ^{13}C) in CD_3OD using residual protic solvent as an internal standard. GC analysis was performed on an Agilent 6850 GC (Agilent, Santa Clara, CA, USA) with a flame ionization detector (FID) and autosampler. Columns used include a 25 m CP-Chirasil-DEX CB column with 0.25 mm inner diameter and 0.25 µm film thickness (Agilent, Santa Clara, CA, USA), a 30 m HP-5 column with 0.32 mm inner diameter and 0.25 µm film thickness (Agilent, Santa Clara, CA, USA) and a 30 m HP-1 column with 0.32 mm inner diameter and 0.25 µm film thickness (Agilent, Santa Clara, CA, USA). Chiral normal phase HPLC was performed on an Agilent system (Santa Clara, CA, USA) equipped with a G1379A degasser, G1312A binary pump, a G1367A well plate autosampler unit, a G1316A temperature controlled column compartment and a G1315C diode array detector. CHIRALPAK®IC and CHIRALPAK®AD-H Analytical (Daicel (Osaka, Japan), 250 mm length, 4.6 mm diameter, 5 µm particle size) columns were used. The typical injection volume was 10 µL and chromatograms were monitored at 254 nm. All solvent mixtures are given in (v/v) ratios. Flash Column Chromatography was performed using silica gel (Sigma-Alrich) 40-63 µm. Glucose dehydrogenase (CDX-901) was purchased from Codexis (Redwood, California, USA).

1.2. Protein Expression

Metagenomic enzymes were obtained from our own collection.[4]

Flask scale expression of the 12 selected metagenomic enzymes was performed by addition of a single colony of transformant from an agar plate into 20 mL LB medium and incubated overnight at 37°C in an orbital shaker at 200 rpm. These starter cultures were used as the inoculum for 0.4 L Formedium's TB medium, supplemented with kanamycin (35 µg / mL) in a 2 L baffled flask. The cultures were then incubated at 37°C at 200 rpm. At an $OD_{600\ nm}$ of between 0.6 and 0.8, IPTG was added to a final concentration of 0.1 mM to induce expression of IREDs. The cultures were grown at 23°C for 20 h at 200 rpm. Cells were harvested by centrifugation at 4000 rpm at 4ºC.

Processing of the *E. coli* cells to crude lyophilised cell-free extract was obtained through resuspension of wet-cell pellet, lysis, and lyophilisation. E. coli cells were resuspended to approx. 5x wet-cell pellet weight in 0.1 M Sodium Phophate pH 7.6. Lysis was undertaken using a MSE Soniprep 150 sonicator with a precooled 9.5 mm probe at an amplitude 16 microns with 1 minute bursts per 50 mL of resuspended cells with 5x repetitions. Lysed cells were clarified using a JA-25.50 Aluminium Fixed-Angle Rotor in a Beckman Coulter centrifuge for 45 minutes at 18,000 rpm. Following clarification the lysate of the E. coli lysate expressing the selected IRED was then lyophilised in a Lyovapor™ L-200 (Buchi, Suffolk, UK). Protein expression was then monitored via 4-20 % SDS-PAGE gels.

1.3. Sodium Dodecyl Sulphate–Polyacrylamide Gel Electrophoresis (SDS-PAGE)

All cell-free extract samples were analysed by SDS-PAGE. Samples were prepped by the addition of 10 mg/ml lyophilised cell free extract, 2x Sample Buffer of Laemmli 2× Concentrate (Sigma-Aldrich) in deionised H_2O. Samples were heated at 100 °C for 10 minutes. 3 µL of Colour Prestained Protein Standard, Broad Range (11-245 kDa) (New England BioLabs) was used. All samples were ran on 4-30 MINI-PROTEAN TGX (BIO-RAD) Gels at 200 V for 45 minutes. Gels were subsequently visualized with Instant Blue Protein Stain (Expedeon). SDS-PAGE analysis was performed on all selected wild-type IREDs.

1.3.1 Protein Purification

Immobilized metal affinity chromatography (IMAC)

Cell pellets were re-suspended to a concentration of 200 mg/mL (5x pellet weight) in 0.1 M sodium phosphate buffer pH 7.0 containing 0.3 M NaCl and 0.03 M imidazole. The cells were then lysed as outlined in section 1.2. Following lysis cell lysate was clarified by centrifugation at 18,000 rpm for 45 min. Supernatant was then filtered through a 0.45 µm MiniStart syringe filter(Satorius) prior to being loaded onto a nickel affinity chromatography column. Using an ÄKTA purifier (GE Healthcare) a HisTrap FF 5 mL column (GE LifeSciences) the column was conditioned with 0.1 M Sodium Phosphate buffer pH 7.0 containing 0.3 M NaCl and 0.03 M imidazole. Once the supernatant was loaded onto the column, the column was washed with 3x column volumes of with 0.1 M sodium phosphate buffer pH 7.0 containing 0.3 M NaCl and 0.03 M imidazole, followed by another wash with with 0.1 M Sodium Phosphate buffer pH 7.0 containing 0.3 M NaCl and 0.06 M imidazole. The protein was then eluted in 0.1 M Sodium Phosphate buffer pH 7.0 containing 0.3 M NaCl and 0.3 M imidazole. Fractions were then analysed by SDS-PAGE and the protein content was checked using NanoDrop 1000 (Thermofisher). Protein was then dialysed into 0.1 M Sodium Phosphate pH 7.0. Following dialysis into 0.1 M Sodium Phosphate buffer pH 7.0, protein concentration was determined by Pierce™ BCA Protein Assay Kit (Thermofisher) and then snap frozen in liquid N2 and stored at -80 °C.

1.4. General procedure for the preparation of standards

For (*R*)-2a, (*S*)-3a, (*R*)-4a and (*S*)-5a

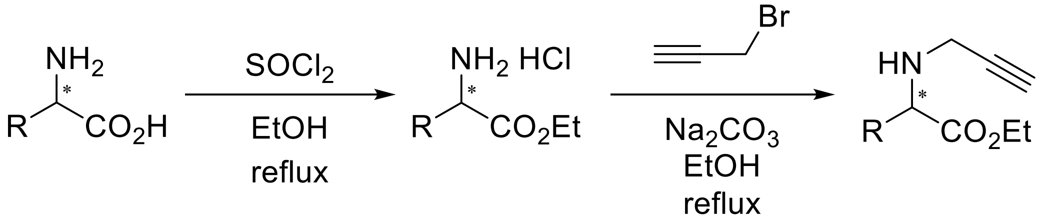

To a mixture of amino acid (5.0 mmol) in EtOH (20 mL) was dropwise added $SOCl_2$ (1.45 mL, 20.0 mmol) at ice water bath. Then the reaction mixture was heated to reflux for 4 hrs. The solvent was removed in *vacuo* to get the amino ester hydrochloride as white solid in quantitative yield.

The amino ester hydrochloride (1 mmol) was then dissolved in 10 mL of EtOH. Sodium carbonate (0.424 g, 4.0 mmol) and 80% propargyl bromide in toluene (0.300 g, 2.0 mmol) were added to the above mixture. The reaction mixture was refluxed for 6 hrs and stirred at room temperature overnight. The reaction mixture was quenched by adding water (50 mL) and extracted with ethyl acetate (2 x 50 mL). The organic phase was dried over anhydrous Na_2SO_4. After evaporation of the solvent in *vacuo*, purification was carried out by column chromatography on silica gel to afford the desired standards.

Ethyl (*R*)-3-phenyl-2-(propargylamino)propionate ((*R*)-2a)

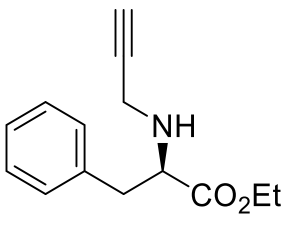

106 mg ethyl (*R*)-3-phenyl-2-(propargylamino)propionate ((*R*)-**2a**) was obtained from 230 mg ethyl D-phenylalaninate hydrochloride in 46% isolated yield as colorless oil. ^{1}H NMR (500MHz, METHANOL-d$_4$) δ = 7.32 - 7.18 (m, 5 H), 4.12 - 4.06 (m, 2 H), 3.76 (dd, *J* = 6.7, 7.6 Hz, 1 H), 3.47 - 3.34 (m, 2 H), 3.03 - 2.92 (m, 2 H), 1.15 (t, *J* = 7.0 Hz, 3 H). ^{13}C NMR (125MHz, METHANOL-d$_4$) δ = 174.91, 138.24, 130.34, 129.47, 127.84, 81.58, 73.53, 62.47, 61.85, 37.01, 14.36. **HRMS** calcd. for $C_{14}H_{18}NO_2^+$ 232.1338 [M+H]$^+$, found 232.1342.

Ethyl (*S*)-2-(propargylamino)propionate ((*S*)-3a)

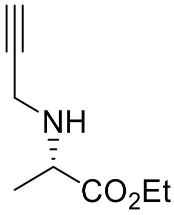

80 mg ethyl (*S*)-2-(propargylamino)propionate ((*S*)-**3a**) was obtained from 154 mg ethyl L-alanine hydrochloride in 52% isolated yield as colorless oil. ^1H NMR (500MHz, METHANOL-d_4) δ = 4.20 (q, *J* = 7.0 Hz, 2 H), 3.58 (q, *J* = 7.0 Hz, 1 H), 3.48 - 3.37 (m, 2 H), 2.63 (t, *J* = 2.3 Hz, 1 H), 1.34 - 1.26 (m, 6 H). ^{13}C NMR (125MHz, METHANOL-d_4) δ = 174.41, 80.10, 72.11, 60.60, 54.36, 35.22, 16.95, 13.09. **HRMS** calcd. for $C_8H_{14}NO_2^+$ 156.1025 [M+H]$^+$, found 156.1025.

Ethyl (*R*)-2-(propargylamino)valerate ((*R*)-4a)

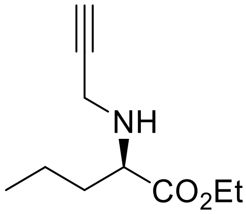

76 mg ethyl (*R*)-2-(propargylamino)valerate ((*R*)-**4a**) was obtained from 182 mg ethyl D-norvaline hydrochloride in 42% isolated yield as colorless oil. ^1H NMR (500MHz, METHANOL-d_4) δ = 4.21 (dq, *J* = 1.7, 7.1 Hz, 2 H), 3.49 (t, *J* = 6.6 Hz, 1 H), 3.47 - 3.34 (m, 2 H), 1.69 - 1.60 (m, 2 H), 1.44 - 1.35 (m, 2 H), 1.30 (t, *J* = 7.2 Hz, 3 H), 0.96 (t, *J* = 7.3 Hz, 3 H). ^{13}C NMR (125MHz, METHANOL-d_4) δ = 174.35, 80.33, 71.98, 60.47, 59.19, 35.65, 34.68, 18.43, 13.16, 12.78. **HRMS** calcd. for $C_{10}H_{18}NO_2^+$ 184.1338 [M+H]$^+$, found 184.1332.

Ethyl (*S*)-4-methyl-2-(propargylamino)valerate ((*S*)-5a)

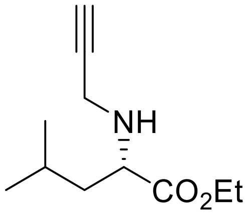

90 mg ethyl (*S*)-4-methyl-2-(propargylamino)valerate ((*S*)-**5a**) was obtained from 196 mg ethyl L-leucinate hydrochloride in 46% isolated yield as colorless oil. ^1H NMR (500MHz, METHANOL-d_4) δ = 4.21 (q, *J* = 7.0 Hz, 2 H), 3.52 (t, *J* = 7.2 Hz, 1 H), 3.47 - 3.34 (m, 2 H), 2.62 (t, *J* = 2.4 Hz, 1 H), 1.74 (quind, *J* = 6.7, 13.6 Hz, 1 H), 1.58 - 1.44 (m, 2 H), 1.30 (t, *J* = 7.2 Hz, 3 H), 0.96 (dd, *J* = 6.7, 8.9 Hz, 6 H). ^{13}C NMR (125MHz, METHANOL-d_4) δ = 174.77, 80.40, 71.95, 60.46, 57.95, 41.96, 35.65, 24.63, 23.00, 21.48, 13.14. **HRMS** calcd. for $C_{11}H_{20}NO_2^+$ 198.1494 [M+H]$^+$, found 198.1486.

For (*R*)-1a, (*S*)-10a and (*R*)-11a

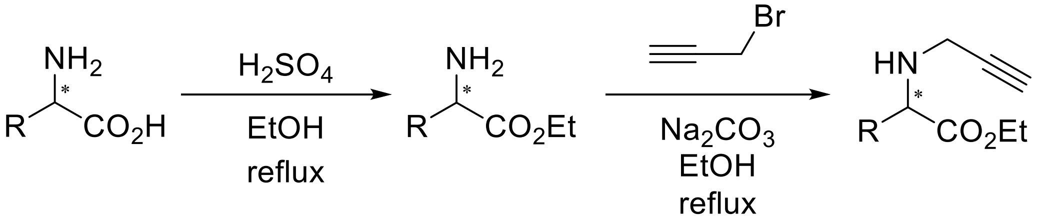

To a mixture of amino acid (5.0 mmol) in EtOH (40 mL) was dropwise added 98% H_2SO_4 (1.0 mL, 18.0 mmol) at room temperature. Then the reaction mixture was heated to reflux for 6 hrs. The solvent was removed by distillationand. The residue was poured into 100 mL 5% sodium carbonate after cooled down to room temperature, and extracted with ethyl acetate (2 x100 mL). The organic phase was dried over anhydrous Na_2SO_4. After evaporation of the solvent in *vacuo*, the desired amino ester was obtained as colorless oil which was used in the next step without further purification.

The amino ester was then dissolved in 40 mL of EtOH. Sodium carbonate (1.06 g, 10 mmol) and 80% propargyl bromide in toluene (1.5 g, 10 mmol) were added to the above mixture. The reaction mixture was refluxed for 6 hrs and stirred at room temperature overnight. The reaction mixture was quenched by adding water (100 mL) and extracted with ethyl acetate (2 x 100 mL). The organic phase was dried over anhydrous Na_2SO_4. After evaporation of the solvent in *vacuo*, purification was carried out by column chromatography on silica gel to afford the desired product as colorless oil. The product was dissolved with 8 mL methyl tert-butyl ether. A solution of HCl in dioxane (4 M, 2 mL) was added and cooled at 4°C for overnight. The corresponding hydrochloride was obtained after filtration, washing with methyl tert-butyl ether (3 x 5 mL), and drying under *vacuo*.

Ethyl (*R*)-4-phenyl-2-(propargylamino)butanoate hydrochloride ((*R*)-1a)

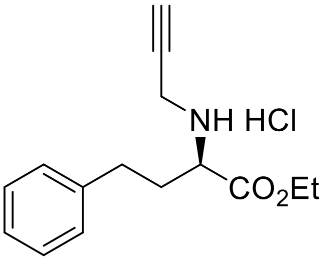

0.79 g ethyl (*R*)-4-phenyl-2-(propargylamino)butanoate hydrochloride ((*R*)-**1a**) was obtained from 0.9 g (*R*)-2-amino-4-phenylbutyric acid in 56% isolated yield as white solid. ^{1}H NMR (400MHz, METHANOL-d$_4$) δ = 7.38 - 7.31 (m, 2 H), 7.31 - 7.22 (m, 3 H), 4.36 (q, *J* = 7.1 Hz, 2 H), 4.23 (dd, *J* = 4.9, 6.8 Hz, 1 H), 4.13 - 4.01 (m, 2 H), 3.34 (br. s., 1 H), 2.93 - 2.80 (m, 1 H), 2.77 - 2.66 (m, 1 H), 2.40 - 2.21 (m, 2 H), 1.39 (t, *J* = 7.1 Hz, 3 H). ^{13}C NMR (100MHz, METHANOL-d$_4$) δ = 168.09, 139.39, 128.38, 128.13, 126.35, 78.55, 72.70, 62.74, 58.13, 35.19, 30.88, 30.35, 13.01. **HRMS** calcd. for $C_{15}H_{20}NO_2^+$ 246.1494 [M+H]$^+$, found 246.1471.

Ethyl (*S*)-2-phenyl-2-(propargylamino)acetate hydrochloride ((*S*)-10a)

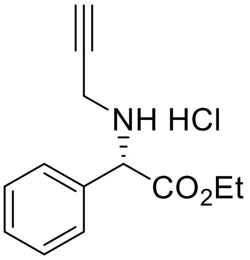

0.60 g ethyl (*S*)-2-phenyl-2-(propargylamino)acetate hydrochloride ((*S*)-**10a**) was obtained from 0.78 g L-phenylglycine in 47% isolated yield as white solid. ^{1}H NMR (400MHz, METHANOL-d$_4$) δ = 7.66 - 7.44 (m, 5 H), 5.32 (s, 1 H), 4.41 - 4.24 (m, 2 H), 3.98 - 3.78 (m, 2 H), 1.25 (t, *J* = 7.1 Hz, 3 H). ^{13}C NMR (100MHz, METHANOL-d$_4$) δ = 167.41, 130.58, 129.57, 128.59, 78.73, 72.56, 62.95, 61.95, 34.56, 12.77. **HRMS** calcd. for $C_{13}H_{16}NO_2^+$ 218.1181 [M+H]$^+$, found 218.1162.

Ethyl *N*-propargyl-D-serinate hydrochloride ((*R*)-11a)

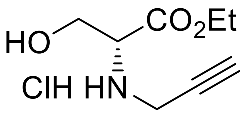

0.26 g ethyl *N*-propargyl-D-serinate hydrochloride ((*R*)-**11a**) was obtained from 0.85 g ethyl D-serinate hydrochloride in 25% isolated yield as colorless oil. ^{1}H NMR (400MHz, METHANOL-d_4) δ = 4.43 - 4.28 (m, 3 H), 4.17 - 4.03 (m, 4 H), 3.35 (br. s., 1 H), 1.37 (t, *J* = 7.0 Hz, 3 H). ^{13}C NMR (100MHz, METHANOL-d_4) δ = 166.89, 78.40, 72.79, 62.66, 60.03, 58.12, 34.75, 12.93. **HRMS** calcd. for $C_8H_{14}NO_3^+$ 172.0974 [M+H]$^+$, found 172.0981.

For Ethyl (*R*)-2-((4-methylbenzyl)amino)-4-phenylbutanoate hydrochloride ((*R*)-1g)

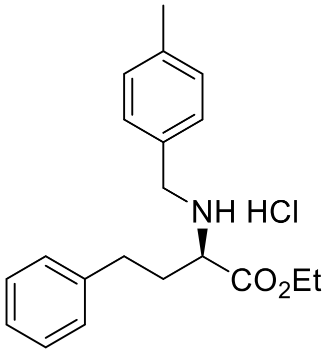

Ethyl (*R*)-4-phenyl-2-aminobutanoate from (*R*)-2-amino-4-phenylbutyric acid (0.90 g, 5.0 mmol) was dissolved in 80 mL of dichloromethane. 4-Methylbenzaldehyde (**g**, 0.60 g, 5.0 mmol) and sodium triacetoxyborohydride (2.11 g, 10.0 mmol) were added to the above mixture. The reaction mixture was stirred at room temperature overnight. The reaction mixture was quenched by adding 5% sodium carbonate (100 mL) and extracted with ethyl acetate (2 x 100 mL). The organic phase was dried over anhydrous Na_2SO_4. After evaporation of the solvent in *vacuo*, purification was carried out by column chromatography on silica gel to afford the desired product. The product was dissolved with 10 mL methyl tert-butyl ether. A solution of HCl in dioxane (4 M, 2 mL) was added and cooled at 4°C for overnight. After filtration, washing with methyl tert-butyl ether (3 x 5 mL) and drying under *vacuo*, 1.12 g ethyl (*R*)-2-((4-methylbenzyl)amino)-4-phenylbutanoate hydrochloride ((*R*)-**1g**) was obtained in 64% isolated yield as white solid. ^{1}H NMR (400MHz, METHANOL-d_4) δ = 7.40 (d, *J* = 7.8 Hz, 2 H), 7.36 - 7.28 (m, 4 H), 7.27 - 7.21 (m, 3 H), 4.35 (q, *J* = 7.3 Hz, 2 H), 4.25 (s, 2 H), 4.05 (dd, *J* = 5.0, 7.7 Hz, 1 H), 2.89 - 2.78 (m, 1 H), 2.77 - 2.65 (m, 1 H), 2.40 (s, 3 H), 2.38 - 2.21 (m, 2 H), 1.39 (t, *J* = 7.1 Hz, 3 H). ^{13}C NMR (100MHz, METHANOL-d_4) δ = 168.37, 139.87, 139.50, 130.00, 129.54, 128.35, 128.12, 127.41, 126.28, 62.62, 58.51, 49.95, 31.28, 30.57, 19.90, 13.05. **HRMS** calcd. for $C_{20}H_{26}NO_2^+$ 312.1964 [M+H]$^+$, found 312.1942.

1.5. Typical Procedure for IRED Catalysed Reductive Aminations

1.5.1. Screening of the 384-well 0.5 mg cell-free extract plates towards ethyl 2-oxo-4-phenylbutyrate (1) and propargylamine (a)

To generate assay master mix reagent: 25 mL of 250 mM D-glucose, 12 U/mL GDH (Codexis CDX-901, 50 U/mg), 0.8 mM NADP$^+$, 50 mM ethyl 2-oxo-4-phenylbutyrate (**1**), and propargylamine (**a**, 100 mM) (added from a pH adjusted (pH 7.5) 1 M stock) in 100 mM sodium phosphate buffer adjusted to pH 7.5 with 10% (v/v) DMSO. Then carefully add 50 µL of the master mix reagent to 384-DeepWell.

Using the microplate sealing film cross-hairs as a guide, carefully add 50 µL of the sodium phosphate buffer (100 mM, pH 7.5) with 10% (v/v) DMSO to each well to dissolve the cell-free extracts and then transfer them to the above 384-DeepWell. Change tips between additions, to avoid enzyme cross contamination. The reaction mixture was incubated at 30ºC with shaking at 200 rpm for 20 h and quenched by the addition of 50 µL of 1 M sodium carbonate and extracted with 200 µL methyl tert-butyl ether. The organic phase was dried over anhydrous $MgSO_4$, and analysed by GC analysis to determine the conversion. In order to eliminate the interference of starting material and by-products such as the alcohol on the HPLC chromatograms, the samples were then acidified by 100 µL of 0.5 M HCl. The organic phase was deserted and the aqueous phase was alkalified by the addition of 50 µL of 1 M sodium carbonate and

extracted with 200 µL methyl tert-butyl ether. The organic phase was dried over anhydrous $MgSO_4$, and analysed by chiral HPLC analysis.

1.5.2. Analytical scale reductive aminations procedure towards ethyl 2-oxo-4-phenylbutyrate (**1**)

For analytical scale reductive aminations a typical 500 µL reaction mixture contained 125 mM D-glucose, 6 U/mL GDH (Codexis CDX-901, 50 U/mg), 0.4 mM $NADP^+$, 50 mg/mL *E. coli* whole cells expressing IRED, 50 mM ethyl 2-oxo-4-phenylbutyrate (**1**), amine (100 mM for **a**, **d, e** and **g**, 500 mM for **b**, **c** and **f**)(added from a pH adjusted 1 M amine stock). These conditions were chosen to obtain the highest activities according to the nature of the amine partner which was based on previous reports.[5,6] The reaction volume was made up to 500 µL with sodium phosphate buffer (100 mM, pH 7.5) with 10% (v/v) DMSO. The reaction mixture was incubated at 30ºC with shaking at 200 rpm for 20 h and quenched by the addition of 200 µL of 1 M sodium carbonate and extracted with 500 µL methyl tert-butyl ether. The organic phase was dried over anhydrous $MgSO_4$, and analysed by GC analysis to determine the conversion. The samples were then acidified by 200 µL of 0.5 M HCl. The organic phase was deserted and the aqueous phase was alkalified by the addition of 200 µL of 1 M sodium carbonate and extracted with 500 µL methyl tert-butyl ether. The organic phase was dried over anhydrous $MgSO_4$, and analysed by chiral HPLC analysis.

1.5.3. Analytical scale reductive aminations procedure towards α-ketoesters with propargylamine (a)

For analytical scale reductive aminations a typical 500 µL reaction mixture contained 125 mM D-glucose, 6 U/mL GDH (Codexis CDX-901, 50 U/mg), 0.4 mM $NADP^+$, 50 mg/mL *E. coli* whole cells expressing IRED, 50 mM α-ketoesters (**2-11**), propargylamine (**a**, 100 mM)(added from a pH adjusted 1 M amine stock). The reaction volume was made up to 500 µL with sodium phosphate buffer (100 mM, pH 7.5) with 10% (v/v) DMSO. The reaction mixture was incubated at 30ºC with shaking at 200 rpm for 20 h and quenched by the addition of 200 µL of 1 M sodium carbonate and extracted with 500 µL methyl tert-butyl ether. The organic phase was dried over anhydrous $MgSO_4$, and analysed by chiral GC analysis to determine the conversion and *ee* values for **3a** (after acetylation by acetic anhydride catalysed by *N*, *N*-dimethylaminopyridine), **4a**, **5a** and **6a**. For **2a**, **7a**, **8a**, **9a** and **10a**, in order to eliminate the interference of starting material and by-products such as the alcohol on the HPLC chromatograms, after determined the conversion by GC analysis, the samples were then acidified by 200 µL of 0.5 M HCl. The organic phase was deserted and the aqueous phase was alkalified by the addition of 200 µL of 1 M sodium carbonate and extracted with 500 µL methyl tert-butyl ether. The organic phase was dried over anhydrous $MgSO_4$, and analysed by chiral HPLC analysis.

1.5.4. Preparative scale Biotransformations

The biotransformation on preparative scale (50 mL) was performed starting from α-keto esters (50 mM), amine (100 mM for **a**, **d**, **e** and **g**, 500 mM for **b** and **c**), cell-free extracts of IRED (2.5 g wet cells weight), D-glucose (125 mM), 16 mg of $NADP^+$, 6 mg GDH (300 U) and 50 mL sodium phosphate buffer (100 mM, pH 7.0) with 5%(v/v) DMSO, and the pH was adjusted to 7.5 with 1 M HCl. The reaction mixture was shaken at 30°C with 200 rpm. The reaction was quenched by the addition of sodium carbonate (4.24 g, 40 mmol) and extracted with ethyl acetate/cyclohexane (1/1, v/v) three times (50 mL×3) with centrifugation (15ºC, 4000 *g*, 10 min) to improve the separation of phases. The organic layers were combined and dried over anhydrous $MgSO_4$. After removal of the solvent under reduced pressure, the residue was dissolved with 5 mL diethyl ether. A solution of HCl in diethyl ether (2 M, 2 mL) was added, the ether layer decanted and the remaining solid or oil washed with diethyl ether (3 x 5 mL) and dried under vacuum to afford the desired product.

Ethyl (*R*)-4-phenyl-2-(propargylamino)butyrate hydrochloride ((*R*)-1a)

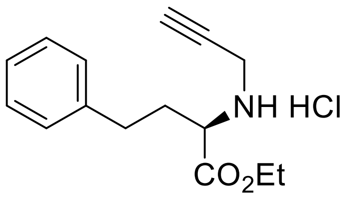

Ethyl 2-oxo-4-phenylbutyrate (**1**, 2.5 mmol, 515 mg) and propargylamine (**a**, 5.0 mmol, 275 mg) catalyzed by pIR-271 with 85% conversion into product and 14% ethyl 2-hydroxyl-4-phenylbutyrate gave 450 mg ethyl (*R*)-4-phenyl-2-(propargylamino)butyrate hydrochloride ((*R*)-**1a**) as brown solid in 64% isolated yield and >99% *ee*. ^{1}H NMR (400MHz, METHANOL-d_4) δ = 7.37 - 7.21 (m, 5 H), 4.34 (q, *J* = 7.2 Hz, 2 H), 4.21 (dd, *J* = 4.9, 7.1 Hz, 1 H), 4.12 - 4.00 (m, 2 H), 3.30 (t, *J* = 2.4 Hz, 1 H), 2.90 - 2.80 (m, 1 H), 2.70 (ddd, *J* = 6.8, 9.6, 13.9 Hz, 1 H), 2.38 - 2.21 (m, 2 H), 1.37 (t, *J* = 7.2 Hz, 3 H). ^{13}C NMR (100MHz, METHANOL-d_4) δ = 168.07, 139.35,

128.37, 128.10, 126.34, 78.52, 72.68, 62.73, 58.09, 35.15, 30.85, 30.31, 12.98. **HRMS** calcd. for $C_{15}H_{20}NO_2^+$ 246.1494 [M+H]$^+$, found 246.1488.

Ethyl (*S*)-4-phenyl-2-(propargylamino)butyrate hydrochloride ((*S*)-1a)

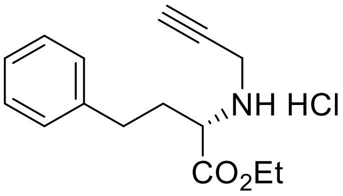

Ethyl 2-oxo-4-phenylbutyrate (**1**, 2.5 mmol, 515 mg) and propargylamine (**a**, 5.0 mmol, 275 mg) catalyzed by pIR-338 with 80% conversion into product and 19% ethyl 2-hydroxyl-4-phenylbutyrate gave 420 mg ethyl (*S*)-4-phenyl-2-(propargylamino)butyrate hydrochloride ((*S*)-**1a**) as brown solid in 60% isolated yield and >99% *ee*. ^{1}H NMR (500MHz, METHANOL-d$_4$) δ = 7.36 - 7.21 (m, 5 H), 4.34 (q, *J* = 7.0 Hz, 2 H), 4.23 - 4.18 (m, 1 H), 4.11 - 4.01 (m, 2 H), 3.29 (t, *J* = 2.4 Hz, 1 H), 2.88 - 2.71 (m, 2 H), 2.37 - 2.22 (m, 2 H), 1.37 (t, *J* = 7.2 Hz, 3 H). ^{13}C NMR (125MHz, METHANOL-d$_4$) δ = 168.07, 139.35, 128.37, 128.10, 126.33, 78.53, 72.67, 62.74, 58.10, 35.15, 30.85, 30.31, 12.98. **HRMS** calcd. for $C_{15}H_{20}NO_2^+$ 246.1494 [M+H]$^+$, found 246.1490.

Ethyl (*R*)-4-phenyl-2-(methylamino)butyrate hydrochloride ((*R*)-1b)

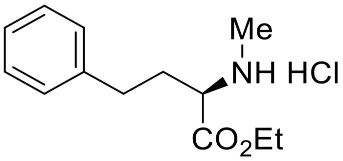

Ethyl 2-oxo-4-phenylbutyrate (**1**, 2.5 mmol, 515 mg) and methylamine hydrochloride (**b**, 25 mmol, 1.688 g) catalyzed by pIR-271 with 92% conversion into product and 8% ethyl 2-hydroxyl-4-phenylbutyrate gave 430 mg ethyl (*R*)-4-phenyl-2-(methylamino)butyrate hydrochloride ((*R*)-**1b**) as white solid in 67% isolated yield and >99% *ee*. ^{1}H NMR (400MHz, METHANOL-d$_4$) δ = 7.37 - 7.20 (m, 5 H), 4.34 (q, *J* = 6.9 Hz, 2 H), 4.06 (t, *J* = 5.7 Hz, 1 H), 2.88 - 2.79 (m, 1 H), 2.77 (s, 3 H), 2.74 - 2.63 (m, 1 H), 2.33 - 2.20 (m, 2 H), 1.37 (t, *J* = 7.1 Hz, 3 H). ^{13}C NMR (100MHz, METHANOL-d$_4$) δ = 168.30, 139.47, 128.37, 128.06, 126.31, 62.56, 60.14, 31.00, 30.77, 30.26, 13.01. **HRMS** calcd. for $C_{13}H_{20}NO_2^+$ 222.1494 [M+H]$^+$, found 222.1489.

Ethyl (*S*)-4-phenyl-2-(methylamino)butyrate hydrochloride ((*S*)-1b)

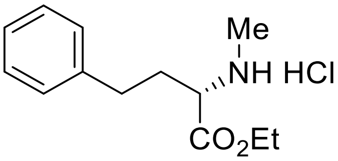

Ethyl 2-oxo-4-phenylbutyrate (**1**, 2.5 mmol, 515 mg) and methylamine hydrochloride (**b**, 25 mmol, 1.688 g) catalyzed by pIR-338 with 95% conversion into product and 5% ethyl 2-hydroxyl-4-phenylbutyrate gave 360 mg ethyl (*S*)-4-phenyl-2-(methylamino)butyrate hydrochloride ((*S*)-**1b**) as white solid in 56% isolated yield and >99% *ee*. ^{1}H NMR (400MHz, METHANOL-d$_4$) δ = 7.37 - 7.21 (m, 5 H), 4.34 (q, *J* = 7.3 Hz, 2 H), 4.05 (t, *J* = 6.0 Hz, 1 H), 2.88 - 2.79 (m, 1 H), 2.77 (s, 3 H), 2.74 - 2.64 (m, 1 H), 2.32 - 2.23 (m, 2 H), 1.37 (t, *J* = 7.1 Hz, 3 H). ^{13}C NMR (100MHz, METHANOL-d$_4$) δ = 168.30, 139.46, 128.37, 128.06, 126.31, 62.56, 60.13, 30.98, 30.76, 30.26, 13.01. **HRMS** calcd. for $C_{13}H_{20}NO_2^+$ 222.1494 [M+H]$^+$, found 222.1492.

Ethyl (*R*)-4-phenyl-2-(propylamino)butyrate hydrochloride ((*R*)-1c)

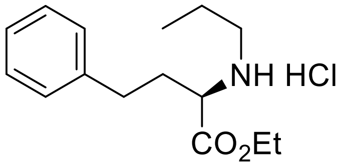

Ethyl 2-oxo-4-phenylbutyrate (**1**, 2.5 mmol, 515 mg) and propylamine (**c**, 25 mmol, 1.475 g) catalyzed by pIR-271 with 91% conversion into product and 9% ethyl 2-hydroxyl-4-phenylbutyrate gave 490 mg ethyl (*R*)-4-phenyl-2-(propylamino)butyrate hydrochloride ((*R*)-**1c**) as white solid in 69% isolated yield and >99% *ee*. ^{1}H NMR (400MHz, METHANOL-d$_4$) δ = 7.36 - 7.20 (m, 5 H), 4.35 (q, *J* = 7.1 Hz, 2 H), 4.07 (dd, *J* = 4.9, 7.3 Hz, 1 H), 3.09 - 2.95 (m, 2 H), 2.92 - 2.79 (m, 1 H), 2.76 - 2.64 (m, 1 H), 2.36 - 2.19 (m, 2 H), 1.81 - 1.70 (m, 2 H), 1.37 (t, *J* = 7.1 Hz, 3 H), 1.04 (t, *J* = 7.5 Hz, 3 H). ^{13}C NMR (100MHz, METHANOL-d$_4$) δ = 168.42, 139.55, 128.36, 128.08, 126.29, 62.56, 59.17, 48.21, 31.08, 30.44, 19.34, 13.03, 9.82. **HRMS** calcd. for $C_{15}H_{24}NO_2^+$ 250.1807 [M+H]$^+$, found 250.1806.

Ethyl (*S*)-4-phenyl-2-(propylamino)butyrate hydrochloride ((*S*)-1c)

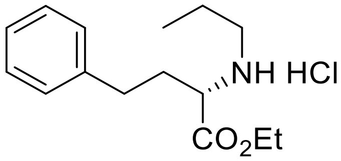

Ethyl 2-oxo-4-phenylbutyrate (**1**, 2.5 mmol, 515 mg) and propylamine (**c**, 25 mmol, 1.475 g) catalyzed by pIR-338 with 62% conversion into product and 38% ethyl 2-hydroxyl-4-phenylbutyrate gave 196 mg ethyl (*S*)-4-phenyl-2-(propylamino)butyrate hydrochloride ((*S*)-**1c**) as white solid in 27% isolated yield and 97% *ee*. ^{1}H NMR (500MHz, METHANOL-d$_4$) δ = 7.36 - 7.21 (m, 5 H), 4.35 (q, *J* = 7.0 Hz, 2 H), 4.06 (dd, *J* = 4.9, 7.3 Hz, 1 H), 3.09 - 2.96 (m, 2 H), 2.93 - 2.80 (m, 2 H), 2.35 - 2.21 (m, 2 H), 1.83 - 1.73 (m, 2 H), 1.37 (t, *J* = 7.2 Hz, 3 H), 1.04 (t, *J* = 7.5 Hz, 3 H). ^{13}C NMR (125MHz ,METHANOL-d$_4$) δ = 168.41, 139.55, 128.36, 128.07, 126.29, 62.57, 59.17, 48.22, 31.07, 30.44, 19.33, 13.02, 9.82. **HRMS** calcd. for $C_{15}H_{24}NO_2^+$ 250.1807 [M+H]$^+$, found 250.1806.

Ethyl (*R*)-4-phenyl-2-(allylamino)butyrate hydrochloride ((*R*)-1d)

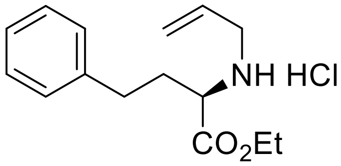

Ethyl 2-oxo-4-phenylbutyrate (**1**, 2.5 mmol, 515 mg) and allylamine (**d**, 5.0 mmol, 285 mg) catalyzed by pIR-271 with 86% conversion into product and 11% ethyl 2-hydroxyl-4-phenylbutyrate gave 410 mg ethyl (*R*)-4-phenyl-2-(allylamino)butyrate hydrochloride ((*R*)-**1d**) as white solid in 58% isolated yield and >99% *ee*. ^{1}H NMR (500MHz, METHANOL-d$_4$) δ = 7.36 - 7.21 (m, 5 H), 5.96 (tdd, *J* = 7.0, 10.1, 17.1 Hz, 1 H), 5.59 - 5.48 (m, 2 H), 4.38 - 4.31 (m, 2 H), 4.07 (dd, *J* = 4.7, 7.5 Hz, 1 H), 3.74 (d, *J* = 7.0 Hz, 2 H), 2.88 - 2.79 (m, 1 H), 2.74 - 2.65 (m, 1 H), 2.36 - 2.21 (m, 2 H), 1.37 (t, *J* = 7.2 Hz, 3 H). ^{13}C NMR (125MHz, METHANOL-d$_4$) δ = 168.33, 139.48, 128.36, 128.10, 127.39, 126.30, 123.61, 62.60, 58.30, 48.57, 31.04, 30.36, 13.02. **HRMS** calcd. for $C_{15}H_{22}NO_2^+$ 248.1651 [M+H]$^+$, found 248.1644.

Ethyl (*S*)-4-phenyl-2-(allylamino)butyrate hydrochloride ((*S*)-1d)

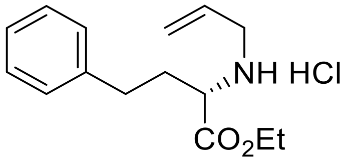

Ethyl 2-oxo-4-phenylbutyrate (**1**, 2.5 mmol, 515 mg) and allylamine (**d**, 5.0 mmol, 285 mg) catalyzed by pIR-338 with 51% conversion into product and 46% ethyl 2-hydroxyl-4-phenylbutyrate gave 240 mg ethyl (*S*)-4-phenyl-2-(allylamino)butyrate hydrochloride ((*S*)-**1d**) as grey solid in 34% isolated yield and >99% *ee*. ^{1}H NMR (500MHz, METHANOL-d$_4$) δ = 7.36 - 7.21 (m, 5 H), 5.96 (tdd, *J* = 7.0, 10.1, 17.1 Hz, 1 H), 5.58 - 5.48 (m, 2 H), 4.34 (q, *J* = 7.2 Hz, 2 H), 4.07 (dd, *J* = 4.7, 7.5 Hz, 1 H), 3.76 - 3.70 (m, 2 H), 2.88 - 2.80 (m, 1 H), 2.74 - 2.65 (m, 1 H), 2.35 - 2.21 (m, 2 H), 1.37 (t, *J* = 7.2 Hz, 3 H). ^{13}C NMR (125MHz, METHANOL-d$_4$) δ = 168.33, 139.48, 128.36, 128.09, 127.39, 126.31, 123.61, 62.60, 58.30, 48.57, 31.04, 30.36, 13.02. **HRMS** calcd. for $C_{15}H_{22}NO_2^+$ 248.1651 [M+H]$^+$, found 248.1646.

Ethyl (*R*)-4-phenyl-2-(cyclopropylamino)butyrate hydrochloride ((*R*)-1e)

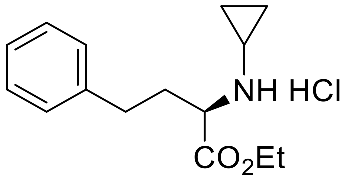

Ethyl 2-oxo-4-phenylbutyrate (**1**, 2.5 mmol, 515 mg) and cyclopropylamine (**e**, 5.0 mmol, 285 mg) catalyzed by pIR-271 with 94% conversion into product and 5% ethyl 2-hydroxyl-4-phenylbutyrate gave 570 mg ethyl (*R*)-4-phenyl-2-(cyclopropylamino)butyrate hydrochloride ((*R*)-**1e**) as white solid in 80% isolated yield and >99% *ee*. ^{1}H NMR (400MHz, METHANOL-d$_4$) δ = 7.37 - 7.20 (m, 5 H), 4.34 (q, *J* = 7.1 Hz, 2 H), 4.20 (dd, *J* = 4.5, 7.5 Hz, 1 H), 2.87 - 2.77 (m, 2 H), 2.74 - 2.63 (m, 1 H), 2.41 - 2.23 (m, 2 H), 1.38 (t, *J* = 7.1 Hz, 3 H), 1.02 - 0.89 (m, 4 H). ^{13}C NMR (100MHz, METHANOL-d$_4$) δ = 166.91, 138.02, 126.89, 126.59, 124.83, 61.08, 58.79, 29.40, 28.81, 27.55, 11.57, 1.53, 1.42. **HRMS** calcd. for $C_{15}H_{22}NO_2^+$ 248.1651 [M+H]$^+$, found 248.1644.

Ethyl (*S*)-4-phenyl-2-(cyclopropylamino)butyrate hydrochloride ((*S*)-1e)

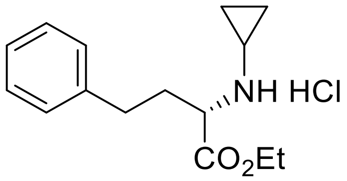

Ethyl 2-oxo-4-phenylbutyrate (**1**, 2.5 mmol, 515 mg) and cyclopropylamine (**e**, 5.0 mmol, 285 mg) catalyzed by pIR-338 with 91% conversion into product and 9% ethyl 2-hydroxyl-4-phenylbutyrate gave 460 mg ethyl (*S*)-4-phenyl-2-(cyclopropylamino)butyrate hydrochloride ((*S*)-**1e**) as white solid in 65% isolated yield and >99% *ee*. ^{1}H NMR (400MHz, METHANOL-d$_4$) δ = 7.36 - 7.21 (m, 5 H), 4.34 (q, *J* = 7.1 Hz, 2 H), 4.20 (dd, *J* = 4.6, 7.6 Hz, 1 H), 2.88 - 2.76 (m, 2 H), 2.69 (ddd, *J* = 6.6, 9.8, 13.9 Hz, 1 H), 2.41 - 2.22 (m, 2 H), 1.38 (t, *J* = 7.1 Hz, 3 H), 1.01 - 0.89 (m, 4 H). ^{13}C NMR (100MHz, METHANOL-d$_4$) δ = 166.91, 138.00, 126.89, 126.58, 124.84, 61.08, 58.77, 29.39, 28.80, 27.52, 11.56, 1.52, 1.42. **HRMS** calcd. for $C_{15}H_{22}NO_2^+$ 248.1651 [M+H]$^+$, found 248.1666.

Ethyl (*R*)-3-phenyl-2-(propargylamino)propionate hydrochloride ((*R*)-2a)

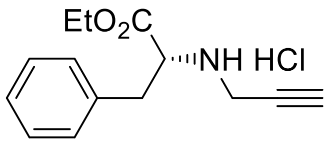

Ethyl phenylpyruvate (**2**, 2.5 mmol, 480 mg) and propargylamine (**a**, 5.0 mmol, 275 mg) catalyzed by pIR-271 with 58% conversion into product and 29% ethyl 2-hydroxyl-3-phenylpropionate gave 181 mg ethyl (*R*)-3-phenyl-2-(propargylamino)propionate hydrochloride ((*R*)-**2a**) as brown oil in 27% isolated yield and >99% *ee*. ^{1}H NMR (400MHz, METHANOL-d$_4$) δ = 7.44 - 7.33 (m, 3 H), 7.33 - 7.28 (m, 2 H), 4.47 (dd, *J* = 5.6, 8.1 Hz, 1 H), 4.26 - 4.16 (m, 2 H), 4.07 (d, *J* = 2.4 Hz, 2 H), 3.46 (dd, *J* = 5.5, 14.1 Hz, 1 H), 3.35 (d, *J* = 2.4 Hz, 1 H), 3.21 (dd, *J* = 8.3, 13.9 Hz, 1 H), 1.18 (t, *J* = 7.1 Hz, 3 H). ^{13}C NMR (100MHz, METHANOL-d$_4$) δ = 167.78, 133.51, 129.12, 128.72, 127.66, 78.67, 72.68, 62.47, 59.76, 35.34, 35.35, 12.72. **HRMS** calcd. for $C_{14}H_{18}NO_2^+$ 232.1338 [M+H]$^+$, found 232.1337.

Ethyl (*S*)-3-phenyl-2-(propargylamino)propionate hydrochloride ((*S*)-2a)

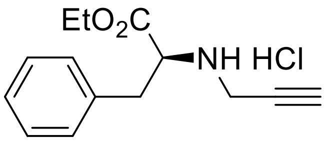

Ethyl phenylpyruvate (**2**, 2.5 mmol, 480 mg) and propargylamine (**a**, 5.0 mmol, 275 mg) catalyzed by pIR-355 with 53% conversion into product and 30% ethyl 2-hydroxyl-3-phenylpropionate gave 190 mg ethyl (*S*)-3-phenyl-2-(propargylamino)propionate hydrochloride ((*S*)-**2a**) as brown oil in 28% isolated yield and >99% *ee*. ^{1}H NMR (400MHz, METHANOL-d$_4$) δ = 7.43 - 7.33 (m, 3 H), 7.33 - 7.26 (m, 2 H), 4.46 (dd, *J* = 5.6, 8.3 Hz, 1 H), 4.25 - 4.17 (m, 2 H), 4.06 (d, *J* = 2.4 Hz, 2 H), 3.45 (dd, *J* = 5.5, 14.1 Hz, 1 H), 3.34 (d, *J* = 1.7 Hz, 1 H), 3.20 (dd, *J* = 8.3, 13.9 Hz, 1 H), 1.17 (t, *J* = 7.1 Hz, 3 H). ^{13}C NMR (100MHz, METHANOL-d$_4$) δ = 167.78, 133.50, 129.11, 128.73, 127.66, 78.66, 72.68, 62.47, 59.75, 35.34, 12.72. **HRMS** calcd. for $C_{14}H_{18}NO_2^+$ 232.1338 [M+H]$^+$, found 232.1333.

Ethyl (*R*)-2-(propargylamino)propionate hydrochloride ((*R*)-3a)

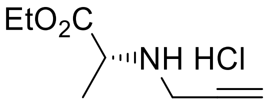

Ethyl pyruvate (**3**, 2.5 mmol, 290 mg) and propargylamine (**a**, 5.0 mmol, 275 mg) catalyzed by pIR-23 with >99% conversion into product gave 240 mg ethyl (*R*)-2-(propargylamino)propionate hydrochloride ((*R*)-**3a**) as brown solid in 50% isolated yield and >99% *ee*. ^{1}H NMR (400MHz, METHANOL-d$_4$) δ = 4.34 (q, *J* = 7.0 Hz, 2 H), 4.24 (q, *J* = 7.3 Hz, 1 H), 4.05 (d, *J* = 2.4 Hz, 2 H), 3.31 (t, *J* = 2.6 Hz, 1 H), 1.61 (d, *J* = 7.3 Hz, 3 H), 1.35 (t, *J* = 7.1 Hz, 3 H). ^{13}C NMR (100MHz, METHANOL-d$_4$) δ = 168.86, 78.25, 72.75, 62.62, 54.24, 34.40, 13.46, 12.89. **HRMS** calcd. for $C_8H_{14}NO_2^+$ 156.1025 [M+H]$^+$, found 156.1023.

Ethyl (*S*)-2-(propargylamino)propionate hydrochloride ((*S*)-3a)

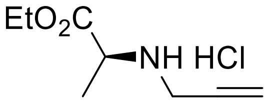

Ethyl pyruvate (**3**, 2.5 mmol, 290 mg) and propargylamine (**a**, 5.0 mmol, 275 mg) catalyzed by pIR-125 with >99% conversion into product gave 208 mg ethyl (*S*)-2-(propargylamino)propionate hydrochloride ((*S*)-**3a**) as brown solid in 43% isolated yield and >99% *ee*. ^{1}H NMR (400MHz, METHANOL-d$_4$) δ = 4.34 (q, *J* = 7.1 Hz, 2 H), 4.24 (q, *J* = 7.1 Hz, 1 H), 4.05 (d, *J* = 2.7 Hz, 2 H), 3.31 (t, *J* = 2.6 Hz, 1 H), 1.61 (d, *J* = 7.3 Hz, 3 H), 1.35 (t, *J* = 7.1 Hz, 3 H). ^{13}C NMR (100MHz, METHANOL-d$_4$) δ = 168.85, 78.25, 72.75, 62.62, 54.24, 34.40, 13.46, 12.89. **HRMS** calcd. for $C_8H_{14}NO_2^+$ 156.1025 [M+H]$^+$, found 156.1024.

Ethyl (*R*)-2-(propargylamino)valerate hydrochloride ((*R*)-4a)

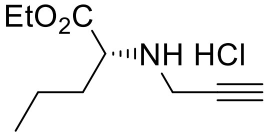

Ethyl 2-oxovalerate (**4**, 2.5 mmol, 360 mg) and propargylamine (**a**, 5.0 mmol, 275 mg) catalyzed by pIR-271 with 91% conversion into product and 9% ethyl 2-hydroxylvalerate gave 230 mg ethyl (*R*)-2-(propargylamino)valerate hydrochloride ((*R*)-**4a**) as brown oil in 42% isolated yield and >99% *ee*. ^{1}H NMR (500MHz, METHANOL-d$_4$) δ = 4.41 - 4.30 (m, 2 H), 4.19 (dd, *J* = 5.0, 6.9 Hz, 1 H), 4.08 - 3.99 (m, 2 H), 2.03 - 1.88 (m, 2 H), 1.57 - 1.38 (m, 2 H), 1.36 (t, *J* = 7.2 Hz, 3 H), 1.03 (t, *J* = 7.3 Hz, 3 H). ^{13}C NMR (125MHz, METHANOL-d$_4$) δ = 168.26, 78.41, 72.73, 62.61, 58.52, 35.05, 30.93, 17.63, 12.92, 12.44. **HRMS** calcd. for $C_{10}H_{18}NO_2^+$ 184.1338 [M+H]$^+$, found 184.1332.

Ethyl (*S*)-2-(propargylamino)valerate hydrochloride ((*S*)-4a)

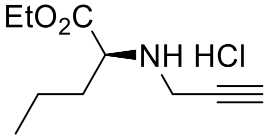

Ethyl 2-oxovalerate (**4**, 2.5 mmol, 360 mg) and propargylamine (**a**, 5.0 mmol, 275 mg) catalyzed by pIR-358 with 95% conversion into product and 5% ethyl 2-hydroxylvalerate gave 315 mg ethyl (*S*)-2-(propargylamino)valerate hydrochloride ((*S*)-**4a**) as brown oil in 57% isolated yield and 96% *ee*. ^{1}H NMR (500MHz, METHANOL-d_4) δ = 4.41 - 4.30 (m, 2 H), 4.20 (dd, *J* = 5.0, 6.9 Hz, 1 H), 4.09 - 4.00 (m, 2 H), 3.31 (t, *J* = 2.4 Hz, 1 H), 2.03 - 1.90 (m, 2 H), 1.57 - 1.38 (m, 2 H), 1.35 (t, *J* = 7.2 Hz, 3 H), 1.02 (t, *J* = 7.3 Hz, 3 H). ^{13}C NMR (125MHz, METHANOL-d_4) δ = 168.25, 78.44, 72.74, 62.58, 58.53, 35.10, 30.95, 17.66, 12.95, 12.47. **HRMS** calcd. for $C_{10}H_{18}NO_2^+$ 184.1338 [M+H]$^+$, found 184.1330.

Ethyl (*R*)-4-methyl-2-(propargylamino)valerate hydrochloride ((*R*)-5a)

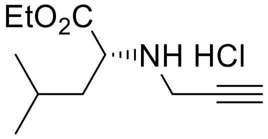

Ethyl 4-methyl-2-oxovalerate (**5**, 2.5 mmol, 395 mg) and propargylamine (**a**, 5.0 mmol, 275 mg) catalyzed by pIR-271 with 98% conversion into product and 2% ethyl 2-hydroxyl-4-methylvalerate gave 280 mg ethyl (*R*)-4-methyl-2-(propargylamino)valerate hydrochloride ((*R*)-**5a**) as brown oil in 48% isolated yield and >99% *ee*. ^{1}H NMR (500MHz, METHANOL-d_4) δ = 4.34 - 4.26 (m, 2 H), 4.13 - 4.09 (m, 1 H), 4.00 (d, *J* = 2.4 Hz, 2 H), 1.85 - 1.69 (m, 3 H), 1.31 (t, *J* = 7.0 Hz, 3 H), 0.98 (t, *J* = 6.1 Hz, 6 H). ^{13}C NMR (125MHz, METHANOL-d_4) δ = 168.52, 78.52, 72.74, 62.62, 57.27, 38.17, 34.99, 24.60, 21.81, 20.39, 12.91. **HRMS** calcd. for $C_{11}H_{20}NO_2^+$ 198.1494 [M+H]$^+$, found 198.1490.

Ethyl (*S*)-4-methyl-2-(propargylamino)valerate hydrochloride ((*S*)-5a)

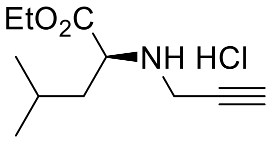

Ethyl 4-methyl-2-oxovalerate (**5**, 2.5 mmol, 395 mg) and propargylamine (**a**, 5.0 mmol, 275 mg) catalyzed by pIR-358 with 98% conversion into product and 2% ethyl 2-hydroxyl-4-methylvalerate gave 320 mg ethyl (*S*)-4-methyl-2-(propargylamino)valerate hydrochloride ((*S*)-**5a**) as brown oil in 55% isolated yield and 72% *ee*. ^{1}H NMR (500MHz, METHANOL-d_4) δ = 4.33 (ttd, *J* = 3.6, 7.1, 10.7 Hz, 2 H), 4.13 (dd, *J* = 5.2, 8.5 Hz, 1 H), 4.06 - 3.98 (m, 2 H), 1.87 - 1.71 (m, 3 H), 1.33 (t, *J* = 7.2 Hz, 3 H), 1.01 (t, *J* = 6.0 Hz, 6 H). ^{13}C NMR (125MHz, METHANOL-d_4) δ = 168.52, 78.52, 72.74, 62.62, 57.27, 38.17, 34.99, 24.60, 21.81, 20.39, 12.91. **HRMS** calcd. for $C_{11}H_{20}NO_2^+$ 198.1494 [M+H]$^+$, found 198.1490.

Ethyl (*R*)-2-(propargylamino)heptanoate hydrochloride ((*R*)-6a)

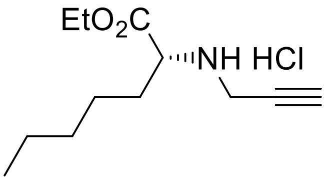

Ethyl 2-oxoheptanoate (**6**, 2.5 mmol, 430 mg) and propargylamine (**a**, 5.0 mmol, 275 mg) catalyzed by pIR-271 with 93% conversion into product and 7% ethyl 2-hydroxylheptanoate gave 380 mg ethyl (*R*)-2-(propargylamino)heptanoate hydrochloride ((*R*)-**6a**) as brown oil in 61% isolated yield and >99% *ee*. ^{1}H NMR (500MHz, METHANOL-d_4) δ = 4.36 - 4.24 (m, 2 H), 4.15 (dd, *J* = 4.9, 7.0 Hz, 1 H), 4.03 - 3.94 (m, 2 H), 3.25 (t, *J* = 2.6 Hz, 1 H), 2.02 - 1.85 (m, 2 H), 1.48 - 1.27 (m, 9 H), 0.93 - 0.84 (m, 3 H). ^{13}C NMR (125MHz, METHANOL-d_4) δ = 168.26, 78.45, 72.73, 62.58, 58.67, 35.13, 30.87, 28.90, 23.84, 21.88, 12.99, 12.81. **HRMS** calcd. for $C_{12}H_{22}NO_2^+$ 212.1651 [M+H]$^+$, found 212.1650.

Ethyl (*S*)-2-(propargylamino)heptanoate hydrochloride ((*S*)-6a)

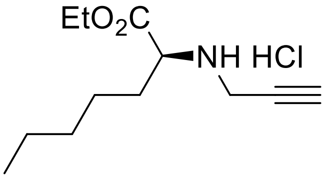

Ethyl 2-oxoheptanoate (**6**, 2.5 mmol, 430 mg) and propargylamine (**a**, 5.0 mmol, 275 mg) catalyzed by pIR-355 with 95% conversion into product and 5% ethyl 2-hydroxylheptanoate gave 368 mg ethyl (*S*)-2-(propargylamino)heptanoate hydrochloride ((*S*)-**6a**) as brown oil in 59% isolated yield and 97% *ee*. ^{1}H NMR (500MHz, METHANOL-d_4) δ = 4.37 - 4.25 (m, 2 H), 4.15 (dd, *J* = 4.9, 7.0 Hz, 1 H), 4.03 - 3.94 (m, 2 H), 3.28 - 3.26 (m, 1 H), 2.02 - 1.86 (m, 2 H), 1.49 - 1.27 (m, 9 H), 0.95 - 0.84 (m, 3 H). ^{13}C NMR (125MHz, METHANOL-d_4) δ = 168.25, 78.43, 72.73, 62.58, 58.67, 35.10, 30.87, 28.88, 23.82, 21.88, 12.96, 12.78. **HRMS** calcd. for $C_{12}H_{22}NO_2^+$ 212.1651 [M+H]$^+$, found 212.1646.

Ethyl (*R*)-2-(methylamino)heptanoate hydrochloride ((*R*)-6b)

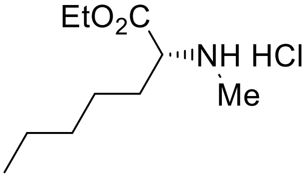

Ethyl 2-oxoheptanoate (**6**, 2.5 mmol, 430 mg) and methylamine (**b**, 25 mmol, 1.688 g) catalyzed by pIR-271 with 97% conversion into product and 3% ethyl 2-hydroxylheptanoate gave 285 mg as white solid in 51% isolated yield and 98% *ee*. ^{1}H NMR (500MHz, METHANOL-d_4) δ = 4.40 - 4.26 (m, 2 H), 4.03 (t, *J* = 5.8 Hz, 1 H), 2.74 (s, 3 H), 2.01 - 1.88 (m, 2 H), 1.50 - 1.31 (m, 9 H), 0.97 - 0.90 (m, 3 H). ^{13}C NMR (125MHz, METHANOL-d_4) δ = 168.51, 62.41, 60.63, 30.96, 30.91, 28.79, 23.73, 21.89, 13.00, 12.79.

Ethyl (*S*)-2-(methylamino)heptanoate hydrochloride ((*S*)-6b)

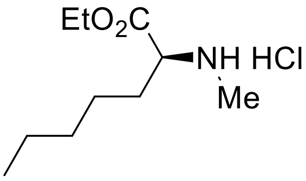

Ethyl 2-oxoheptanoate (**6**, 2.5 mmol, 430 mg) and methylamine (**b**, 25 mmol, 1.688 g) catalyzed by pIR-358 with 98% conversion into product and 2% ethyl 2-hydroxylheptanoate gave 310 mg as white solid in 56% isolated yield and 90% *ee*. ^{1}H NMR (400MHz, METHANOL-d_4) δ = 4.43 - 4.26 (m, 2 H), 4.04 (t, *J* = 5.9 Hz, 1 H), 2.75 (s, 3 H), 2.04 - 1.87 (m, 2 H), 1.52 - 1.43 (m, 1 H), 1.43 - 1.27 (m, 8 H), 0.98 - 0.87 (m, 3 H). ^{13}C NMR (125MHz, METHANOL-d_4) δ = 168.49, 62.38, 60.63, 30.99, 30.91, 28.80, 23.74, 21.89, 13.01, 12.80.

Ethyl (*R*)-4-(2-fluorophenyl)-2-(propargylamino)butanoate hydrochloride ((*R*)-7a)

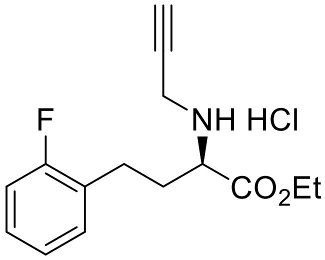

Ethyl 2-oxo-4-(2-fluorophenyl)butyrate (**7**, 2.5 mmol, 560 mg) and propargylamine (**a**, 5.0 mmol, 275 mg) catalyzed by pIR-271 with 67% conversion into product and 33% ethyl 2-hydroxyl-4-(2-fluorophenyl)butyrate gave 298 mg ethyl (*R*)-4-(2-fluorophenyl)-2-(propargylamino)butyrate hydrochloride ((*R*)-**7a**) as brown solid in 40% isolated yield and >99% *ee*. ^{1}H NMR (400MHz, METHANOL-d_4) δ = 7.40 - 7.31 (m, 1 H), 7.11 (d, *J* = 7.6 Hz, 1 H), 7.05 (d, *J* = 10.0 Hz, 1 H), 7.00 (t, *J* = 8.6 Hz, 1 H), 4.36 (q, *J* = 7.1 Hz, 2 H), 4.23 (dd, *J* = 4.8, 7.2 Hz, 1 H), 4.15 - 4.03 (m, 2 H), 3.34 (br. s., 1 H), 2.94 - 2.84 (m, 1 H), 2.80 - 2.69 (m, 1 H), 2.42 - 2.22 (m, 2 H), 1.39 (t, *J* = 7.1 Hz, 3 H). ^{13}C NMR (100MHz, METHANOL-d_4) δ = 167.98, 163.04 (*J* = 243 Hz), 142.22 (*J* = 7 Hz), 130.11 (*J* = 8 Hz), 124.07 (*J* = 3 Hz), 114.89 (*J* = 22 Hz), 113.05 (*J* = 21 Hz), 78.57, 72.69, 62.80, 58.01, 35.18, 30.46, 30.08, 12.99. **HRMS** calcd. for $C_{15}H_{19}FNO_2^+$ 264.1400 [M+H]$^+$, found 264.1394.

Ethyl (*S*)-4-(2-fluorophenyl)-2-(propargylamino)butanoate hydrochloride ((*S*)-7a)

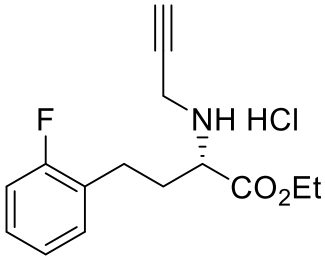

Ethyl 2-oxo-4-(2-fluorophenyl)butyrate (**7**, 2.5 mmol, 560 mg) and propargylamine (**a**, 5.0 mmol, 275 mg) catalyzed by pIR-338 with 87% conversion into product and 11% ethyl 2-hydroxyl-4-(2-fluorophenyl)butyrate gave 306 mg ethyl (*S*)-4-(2-fluorophenyl)-2-(propargylamino)butyrate hydrochloride ((*S*)-**7a**) as brown solid in 41% isolated yield and 64% *ee*.^{1}H NMR (400MHz, METHANOL-d_4) δ = 7.37 (q, *J* = 7.5 Hz, 1 H), 7.12 (d, *J* = 7.6 Hz, 1 H), 7.06 (d, *J* = 10.0 Hz, 1 H), 7.00 (t, *J* = 8.6 Hz, 1 H), 4.36 (q, *J* = 7.1 Hz, 2 H), 4.23 (dd, *J* = 4.9, 7.1 Hz, 1 H), 4.15 - 4.02 (m, 2 H), 3.34 (s, 1 H), 2.96 - 2.83 (m, 1 H), 2.81 - 2.68 (m, 1 H), 2.41 - 2.22 (m, 2 H), 1.39 (t, *J* = 7.1 Hz, 3 H). ^{13}C NMR (100MHz, METHANOL-d_4) δ = 167.98, 163.03 (*J* = 244 Hz), 142.20 (*J* = 7 Hz), 130.11 (*J* = 8 Hz), 124.06 (*J* = 3 Hz), 114.88 (*J* = 22 Hz), 113.05 (*J* = 21 Hz), 78.56, 72.69, 62.80, 58.01, 35.17, 30.46, 30.07, 12.98. **HRMS** calcd. for $C_{15}H_{19}FNO_2^+$ 264.1400 [M+H]$^+$, found 264.1392.

Ethyl (*R*)-4-(3-fluorophenyl)-2-(propargylamino)butanoate hydrochloride ((*R*)-8a)

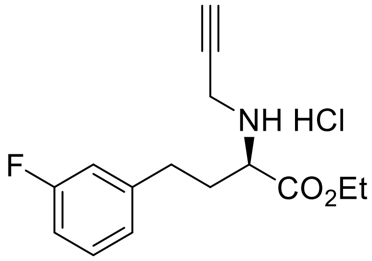

Ethyl 2-oxo-4-(3-fluorophenyl)butyrate (**8**, 2.5 mmol, 560 mg) and propargylamine (**a**, 5.0 mmol, 275 mg) catalyzed by pIR-271 with 69% conversion into product and 30% ethyl 2-hydroxyl-4-(3-fluorophenyl)butyrate gave 310 mg ethyl (*R*)-4-(3-fluorophenyl)-2-(propargylamino)butyrate hydrochloride ((*R*)-**8a**) as brown solid in 42% isolated yield and >99% *ee*.^{1}H NMR (400MHz, METHANOL-d_4) δ = 7.38 - 7.25 (m, 2 H), 7.21 - 7.06 (m, 2 H), 4.37 (ttd, *J* = 3.5, 7.0, 10.6 Hz, 2 H), 4.27 (dd, *J* = 4.6, 7.1 Hz, 1 H), 4.16 - 4.02 (m, 2 H), 3.34 (br. s., 1 H), 2.96 - 2.85 (m, 1 H), 2.83 - 2.71 (m, 1 H), 2.42 - 2.21 (m, 2 H), 1.39 (t, *J* = 7.1 Hz, 3 H). ^{13}C NMR (100MHz, METHANOL-d_4) δ = 167.98, 161.08 (*J* = 243 Hz), 130.50 (*J* = 5 Hz), 128.53 (*J* = 9 Hz), 126.16 (*J* = 15 Hz), 114.26 (*J* = 2 Hz), 115.00

(J = 22 Hz), 78.59, 72.65, 62.81, 58.15, 35.19, 29.37, 23.94, 12.96. **HRMS** calcd. for $C_{15}H_{19}FNO_2^+$ 264.1400 [M+H]$^+$, found 264.1384.

Ethyl (*S*)-4-(3-fluorophenyl)-2-(propargylamino)butanoate hydrochloride ((*S*)-8a)

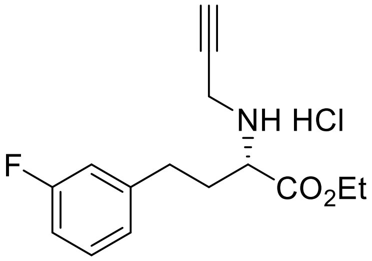

Ethyl 2-oxo-4-(3-fluorophenyl)butyrate (**8**, 2.5 mmol, 560 mg) and propargylamine (**a**, 5.0 mmol, 275 mg) catalyzed by pIR-338 with 87% conversion into product and 12% ethyl 2-hydroxyl-4-(3-fluorophenyl)butyrate gave 360 mg ethyl (*S*)-4-(2-fluorophenyl)-2-(propargylamino)butyrate hydrochloride ((*S*)-**7a**) as brown solid in 41% isolated yield and 26% *ee*. ^{1}H NMR (400MHz, METHANOL-d$_4$) δ = 7.38 - 7.26 (m, 2 H), 7.21 - 7.07 (m, 2 H), 4.38 (ttd, J = 3.6, 7.1, 10.6 Hz, 2 H), 4.26 (dd, J = 4.6, 7.1 Hz, 1 H), 4.15 - 4.01 (m, 2 H), 3.35 (br. s., 1 H), 2.96 - 2.85 (m, 1 H), 2.83 - 2.71 (m, 1 H), 2.41 - 2.20 (m, 2 H), 1.39 (t, J = 7.1 Hz, 3 H). ^{13}C NMR (100MHz, METHANOL-d$_4$) δ = 168.03, 161.08 (J = 243 Hz), 130.49 (J = 5 Hz), 128.53 (J = 8 Hz), 126.16 (J = 15 Hz), 114.26 (J = 4 Hz), 115.00 (J = 22 Hz), 78.53, 72.71, 62.80, 58.16, 35.17, 29.39, 23.95, 12.96. **HRMS** calcd. for $C_{15}H_{19}FNO_2^+$ 264.1400 [M+H]$^+$, found 264.1393.

Ethyl (*R*)-4-(4-fluorophenyl)-2-(propargylamino)butanoate hydrochloride ((*R*)-9a)

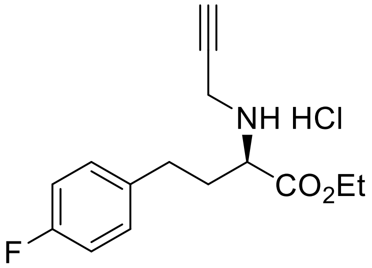

Ethyl 2-oxo-4-(4-fluorophenyl)butyrate (**9**, 2.5 mmol, 560 mg) and propargylamine (**a**, 5.0 mmol, 275 mg) catalyzed by pIR-271 with 73% conversion into product and 26% ethyl 2-hydroxyl-4-(4-fluorophenyl)butyrate gave 370 mg ethyl (*R*)-4-(4-fluorophenyl)-2-(propargylamino)butyrate hydrochloride ((*R*)-**9a**) as brown solid in 50% isolated yield and >99% *ee*.^{1}H NMR (400MHz, METHANOL-d$_4$) δ = 7.31 (dd, J = 5.5, 8.2 Hz, 2 H), 7.08 (t, J = 8.8 Hz, 2 H), 4.37 (q, J = 7.1 Hz, 2 H), 4.22 (dd, J = 4.9, 7.1 Hz, 1 H), 4.14 - 4.01 (m, 2 H), 3.34 (s, 1 H), 2.92 - 2.79 (m, 1 H), 2.77 - 2.63 (m, 1 H), 2.39 - 2.19 (m, 2 H), 1.39 (t, J = 7.1 Hz, 3 H). ^{13}C NMR (100MHz, METHANOL-d$_4$) δ = 168.05, 161.76 (J = 242 Hz), 135.34 (J = 3 Hz), 129.88 (J = 8 Hz), 114.96 (J = 22 Hz), 78.55, 72.72, 62.77, 58.04, 35.18, 30.88, 29.56, 13.00. **HRMS** calcd. for $C_{15}H_{19}FNO_2^+$ 264.1400 [M+H]$^+$, found 264.1391.

Ethyl (*S*)-4-(4-fluorophenyl)-2-(propargylamino)butanoate hydrochloride ((*S*)-9a)

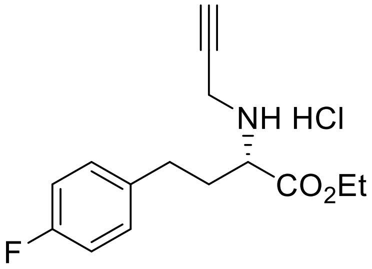

Ethyl 2-oxo-4-(4-fluorophenyl)butyrate (**9**, 2.5 mmol, 560 mg) and propargylamine (**a**, 5.0 mmol, 275 mg) catalyzed by pIR-338 with 93% conversion into product and 6% ethyl 2-hydroxyl-4-(4-fluorophenyl)butyrate gave 400 mg ethyl (*S*)-4-(2-fluorophenyl)-2-(propargylamino)butyrate hydrochloride ((*S*)-**9a**) as brown solid in 54% isolated yield and 70% *ee*. ^{1}H NMR (400MHz, METHANOL-d$_4$) δ = 7.31 (dd, J = 5.6, 8.1 Hz, 2 H), 7.07 (t, J = 8.7 Hz, 2 H), 4.37 (q, J = 7.1 Hz, 2 H), 4.22 (dd, J = 4.9, 7.1 Hz, 1 H), 4.15 - 4.02 (m, 2

H), 3.34 (br. s., 1 H), 2.92 - 2.80 (m, 1 H), 2.77 - 2.66 (m, 1 H), 2.39 - 2.20 (m, 2 H), 1.39 (t, J = 7.1 Hz, 3 H). ^{13}C NMR (100MHz, METHANOL-d$_4$) δ = 168.04, 161.76 (J = 242 Hz), 135.34 (J = 3 Hz), 129.89 (J = 8 Hz), 114.96 (J = 21 Hz), 78.55, 72.72, 62.77, 58.04, 35.18, 30.88, 29.56, 13.01. **HRMS** calcd. for $C_{15}H_{19}FNO_2^+$ 264.1400 [M+H]$^+$, found 264.1387.

2. Supporting Tables

Table S1. Chiral GC analysis: methods and retention times. injector temperature: 200ºC, inlet pressure: 13.5 psi, detector temperature: 220ºC, helium flow: 2.0 mL/min, column: CP-ChiraSil-DEX CB. Absolute configurations were assigned by comparison with standards of known configuration.

2-Ketoester	Amine	Product	Oven temp.	2-Ketoester retention time/min	Product retention time/min
**3**	**a**	**3a**[a]	*	1.75	18.06(*S*)<br>18.61(*R*)
**4**	**a**	**4a**	**	6.79	12.74(*R*)<br>12.83(*S*)
**5**	**a**	**5a**	**	7.83	13.52(*R*)<br>13.63(*S*)
**6**	**a**	**6a**	**	11.75	16.88(*R*)<br>16.95(*S*)
**6**	**b**	**6b**	**	11.75	12.54(*R*)<br>12.62(*S*)

[a] derivatization with acetic anhydride. * oven temperature: Hold at 120ºC for 20 min. ** oven temperature: Hold at 60ºC for 0 min, 60 – 150ºC (5ºC/min), hold at 150ºC for 2 min.

Table S2. GC-FID analysis: methods and retention times. injector temperature: 200ºC, inlet pressure: 22.40 psi, detector temperature: 220ºC, helium flow: 2.0 mL/min, column: HP-1.

2-Ketoester	Amine	Product	Column	Oven temp.	Ketone retention time/min	Product retention time/min
**1**	**a**	**1a**	HP-1	**	6.74	9.32
**1**	**b**	**1b**	HP-1	**	6.74	7.54
**1**	**c**	**1c**	HP-1	**	6.74	9.06
**1**	**d**	**1d**	HP-1	**	6.74	8.98
**1**	**e**	**1e**	HP-1	**	6.74	9.39
**2**	**a**	**2a**	HP-1	**	5.60	8.01
**1**	**g**	**1g**	HP-5	****	7.70	15.47
**7**	**a**	**7a**	HP-5	***	7.70	10.20
**8**	**a**	**8a**	HP-5	***	7.63	10.19
**9**	**a**	**9a**	HP-5	***	7.74	10.26
**10**	**a**	**10a**	HP-5	**	5.55	7.92

** oven temperature: Hold at 100ºC for 2 min, 100 – 180ºC (10ºC/min), hold at 180ºC for 2 min. *** oven temperature: Hold at 100ºC for 2 min, 100 – 200ºC (10ºC/min), hold at 200ºC for 1 min. **** oven temperature: Hold at 100ºC for 2 min, 100 – 260ºC (10ºC/min), hold at 260ºC for 2 min.

Table S3. Conversion and enantioselectivity of selected IREDs towards ethyl 2-oxo-4-phenylbutyrate (**1**) and propargylamine (**a**)[a].

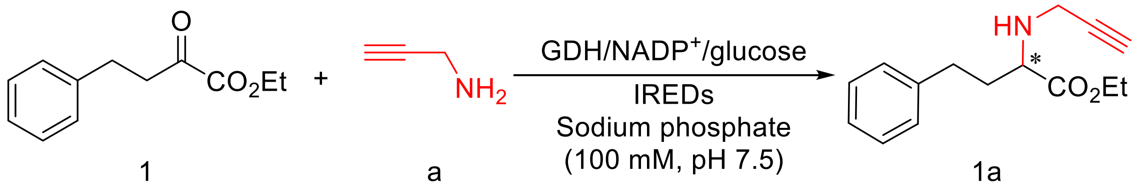

1 a 1a

Entry	IRED (pIR-)	*ee*[b] (%)	conv[c] (%)	Entry	IRED (pIR-)	*ee*[b] (%)	conv[c] (%)	Entry	IRED (pIR-)	*ee*[b] (%)	conv[c] (%)
1	1	24^{S}	65	34	88	10^{R}	80	67	207	74^{R}	68
2	2	66^{R}	17	35	89	80^{R}	73	68	223	84^{R}	87
3	3	25^{R}	59	36	90	99^{R}	20	69	228	99^{R}	79
4	4	74^{R}	67	37	94	37^{R}	59	70	230	76^{R}	85
5	6	62^{R}	60	38	103	72^{S}	59	71	234	48^{R}	67
6	7	99^{R}	27	39	104	56^{R}	85	72	236	99^{R}	55
7	8	87^{R}	47	40	105	60^{R}	82	73	238	42^{R}	59
8	9	54^{S}	77	41	106	78^{S}	92	74	241	40^{S}	75
9	12	22^{R}	77	42	107	60^{R}	80	75	242	44^{R}	63
10	13	24^{R}	66	43	110	10^{R}	68	76	246	58^{R}	87
11	14	59^{R}	16	44	112	99^{R}	63	77	258	99^{R}	91
12	16	58^{S}	82	45	114	48^{R}	83	78	259	99^{R}	76
13	17	52^{R}	54	46	117	99^{R}	86	79	260	99^{R}	63
14	20	99^{R}	31	47	120	60^{S}	69	80	265	58^{R}	91
15	23	99^{R}	64	48	121	0	61	81	268	99^{R}	35
16	28	82^{R}	31	49	122	29^{R}	75	82	270	99^{R}	62
17	31	50^{R}	61	50	123	90^{S}	69	83	271	99^{R}	92
18	33	65^{S}	43	51	125	64^{S}	84	84	273	99^{R}	74
19	37	99^{R}	12	52	128	78^{R}	79	85	310	99^{R}	54
20	45	92^{R}	50	53	129	99^{R}	78	86	324	99^{R}	54
21	48	99^{R}	15	54	134	99^{R}	47	87	325	99^{R}	93
22	49	54^{R}	56	55	143	99^{R}	71	88	333	10^{R}	69
23	50	31^{S}	78	56	146	68^{S}	80	89	338	99^{S}	92
24	55	99^{R}	21	57	148	76^{R}	89	90	339	26^{R}	93
25	57	12^{S}	60	58	151	99^{R}	55	91	351	37^{R}	70
26	58	40^{R}	42	59	173	99^{R}	67	92	355	79^{S}	91
27	62	99^{R}	50	60	174	99^{R}	51	93	356	39^{S}	77
28	65	99^{R}	35	61	186	36^{R}	87	94	357	99^{R}	88
29	66	99^{R}	23	62	197	82^{R}	79	95	358	68^{S}	96
30	73	99^{R}	42	63	201	18^{S}	87	96	360	99^{R}	47
31	78	62^{S}	73	64	202	34^{S}	89	97	361	99^{R}	92
32	79	78^{R}	47	65	204	82^{R}	89	98	364	99^{R}	53
33	82	77^{R}	52	66	206	18^{R}	45	99	374	30^{R}	90

[a] Reaction conditions: 25 mM ethyl 2-oxo-4-phenylbutyrate (**1**), propargylamine (**a**, 50 mM), 5 mg/mL lysate of *E. coli* expressing IRED, 6 U/mL CDX-901 GDH, 0.4 mM $NADP^+$, 62.5 mM glucose, 10% (v/v) DMSO, sodium phosphate buffer (100 mM, pH 7.5), 100 µL reaction volume, 30 °C, 200 rpm, 20 h, [b] Enantiomeric excess (*ee*) was determined by chiral HPLC. [c] Conversion into product was determined by GC according to the area of substrate, by-product alcohol and product.

Table S4. Conversion and enantioselectivity of selected IREDs towards α-ketoesters (**2**-**11**) and propargylamine (**a**)[a].

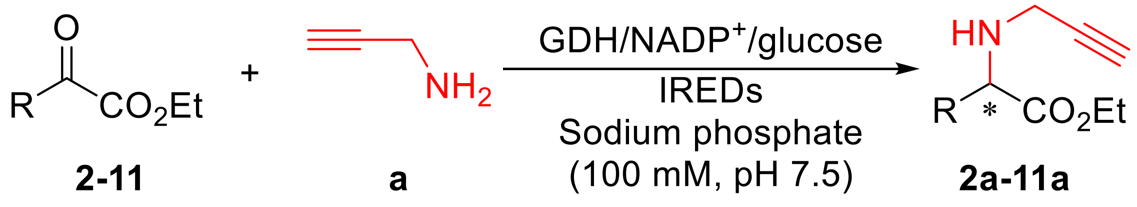

R = Benzyl (**2**), Me (**3**), Pr (**4**), iBu (**5**), Pentyl (**6**), 2-(2-Fluorophenyl)ethyl (**7**), 2-(3-Fluorophenyl)ethyl (**8**), 2-(4-Fluorophenyl)ethyl (**9**), Phenyl (**10**), $HOCH_2$(**11**)

Entry	IRED (pIR-)	Conversion[b] (%) and *ee* values[c] (%)															
		**2a**		**3a**		**4a**		**5a**		**6a**		**7a**		**8a**		**9a**	
1	23	38	99^R	99	99^R	80	97^R	67	90^R	98	99^R	75	99^R	73	99^R	72	94^R
2	106	63	98^S	99	99^S	93	60^S	90	12^R	99	93^S	77	67^S	70	56^S	80	72^S
3	117	49	99^R	99	97^S	88	96^R	73	96^R	98	99^R	54	99^R	43	99^R	35	78^R
4	125	65	91^S	99	99^S	95	60^S	93	8^R	99	88^S	80	27^S	76	65^S	85	84^S
5	258	44	99^R	99	94^S	88	93^R	66	94^R	98	99^R	60	91^R	55	92^R	65	84^R
6	271	58	99^R	99	99^R	94	97^R	92	98^R	99	99^R	66	99^R	71	99^R	71	99^R
7	325	48	99^R	99	99^R	89	95^R	83	82^R	98	99^R	77	99^R	63	99^R	73	80^R
8	338	8	ND^d	0	ND^d	56	94^S	65	79^S	95	83^S	81	57^S	77	21^S	91	82^S
9	355	51	99^S	99	41^R	96	38^R	67	1^S	99	97^S	31	99^R	37	99^R	53	90^R
10	357	0	ND^d	99	58^S	35	4^S	29	85^R	91	94^R	26	99^R	26	99^R	41	48^R
11	358	69	72^S	99	89^S	96	94^S	93	72^S	99	95^S	79	33^S	76	50^S	88	72^S
12	361	62	68^R	99	65^S	95	98^R	88	88^R	99	95^R	76	99^R	76	99^R	86	88^R

[a] Reaction conditions: 50 mM α-ketoesters (**2**-**6**), 100 mM propargylamine (**a**), 50 mg/mL E. coli whole cells expressing IRED, 6 U/mL CDX-901 GDH, 0.4 mM NADP+, 125 mM glucose, 10% (v/v) DMSO, sodium phosphate buffer (100 mM, pH 7.5), 500 μL reaction volume, 30 °C, 200 rpm, 20 h. [b] Conversion into product was determined by GC according to the area of substrate, by-product alcohol and product. [c] Enantiomeric excess (*ee*) was determined by chiral HPLC or GC. d Not determined owing to low conversion, and no IREDs showed activity towards **10** and **11**.

3. Supporting Figures

3.1. SDS-PAGE analysis of the selected IREDs

Figure S1. SDS-PAGE analysis of the selected IREDs recombinantly expressed in *E. coli* BL21 (DE3). All samples were made up of 10 mg/ml crude lyophilised lysate. Lane M: Molecular Weight Marker(Page Ruler Plus™ Prestained Protein Ladder), 1: pIR-23, 2: pIR-106, 3: pIR-117, 4: pIR-125, 5: pIR-258, 6: pIR- 271, 7: pIR-325, 8: pIR-338, 9: pIR-355, 10: pIR-357, 11: pIR-358, 12: pIR-361.

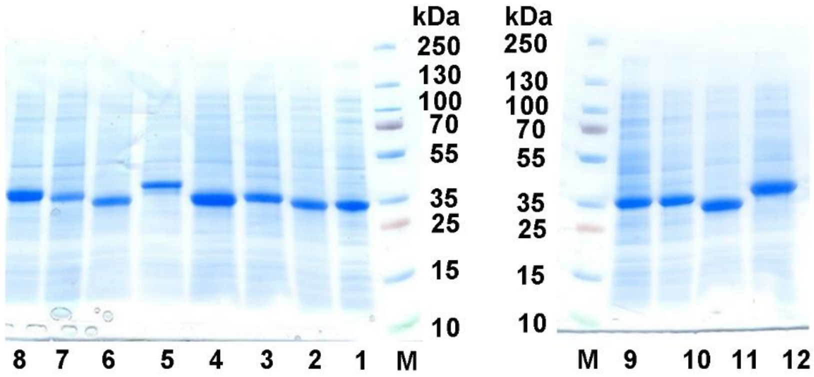

3.2. Results of preparative scale reductive aminations of α-ketoesters with different amines

Figure S2. An expansion of **Figure 3** in the manuscript.

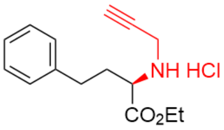<br>(R)-1a<br>pIR-271<br>conv. 85%(99%*ee*)<br>64% isolated yield	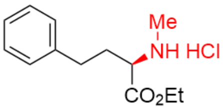<br>(R)-1b<br>pIR-271<br>conv. 92%(99%*ee*)<br>67% isolated yield	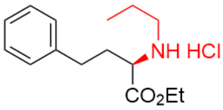<br>(R)-1c<br>pIR-271<br>conv. 91%(99%*ee*)<br>69% isolated yield	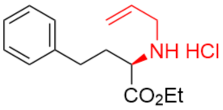<br>(R)-1d<br>pIR-271<br>conv. 86%(99%*ee*)<br>58% isolated yield
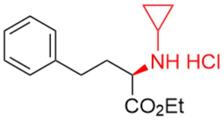<br>(*R*)-1e<br>pIR-271<br>conv. 94%(99%*ee*)<br>80% isolated yield	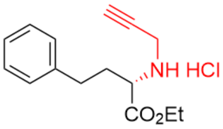<br>(*S*)-1a<br>pIR-338<br>conv. 80%(99%*ee*)<br>60% isolated yield	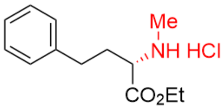<br>(*S*)-1b<br>pIR-338<br>conv. 95%(99%*ee*)<br>56% isolated yield	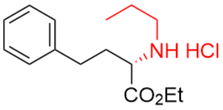<br>(*S*)-1c<br>pIR-338<br>conv. 62%(97%*ee*)<br>27% isolated yield
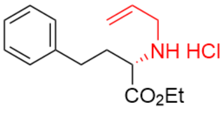<br>(*S*)-1d<br>pIR-338<br>conv. 51%(99%*ee*)<br>34% isolated yield	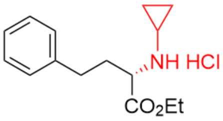<br>(*S*)-1e<br>pIR-338<br>conv. 91%(99%*ee*)<br>65% isolated yield	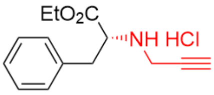<br>(*R*)-2a<br>pIR-271<br>conv. 58%(99%*ee*)<br>27% isolated yield	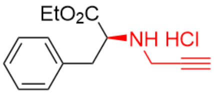<br>(*S*)-2a<br>pIR-355<br>conv. 53%(99%*ee*)<br>28% isolated yield
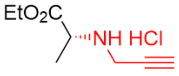<br>(*R*)-3a<br>pIR-23<br>conv. 99%(99%*ee*)<br>50% isolated yield	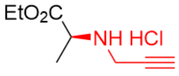<br>(*S*)-3a<br>pIR-125<br>conv. 99%(99%*ee*)<br>43% isolated yield	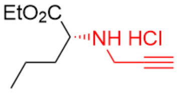<br>(*R*)-4a<br>pIR-271<br>conv. 91%(99%*ee*)<br>42% isolated yield	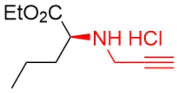<br>(*S*)-4a<br>pIR-358<br>conv. 95%(96%*ee*)<br>57% isolated yield
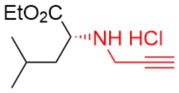<br>(*R*)-5a<br>pIR-271<br>conv. 98%(98%*ee*)<br>48% isolated yield	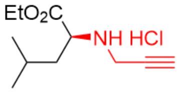<br>(*S*)-5a<br>pIR-358<br>conv. 98%(72%*ee*)<br>55% isolated yield	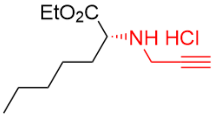<br>(*R*)-6a<br>pIR-271<br>conv. 93%(99%*ee*)<br>61% isolated yield	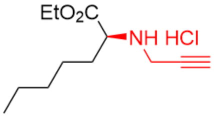<br>(*S*)-6a<br>pIR-355<br>conv. 95%(97%*ee*)<br>59% isolated yield
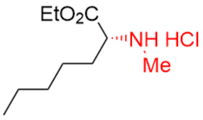<br>(*R*)-6b<br>pIR-271<br>conv. 97%(98%*ee*)<br>51% isolated yield	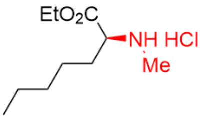<br>(*S*)-6b<br>pIR-358<br>conv. 98%(90%*ee*)<br>56% isolated yield	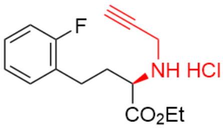<br>(*R*)-7a<br>pIR-271<br>conv. 67%(99%*ee*)<br>40% isolated yield	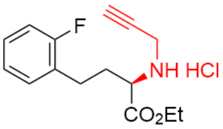<br>(*S*)-7a<br>pIR-338<br>conv. 87%(64%*ee*)<br>41% isolated yield
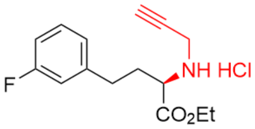<br>(*R*)-8a<br>pIR-271<br>conv.69%(99%*ee*)<br>42% isolated yield	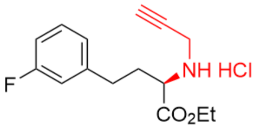<br>(*S*)-8a<br>pIR-338<br>conv. 87%(26%*ee*)<br>48% isolated yield	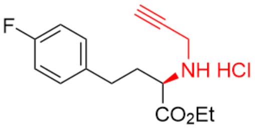<br>(*R*)-7a<br>pIR-271<br>conv. 73%(99%*ee*)<br>50% isolated yield	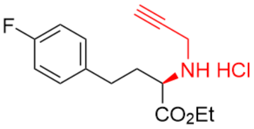<br>(*S*)-7a<br>pIR-338<br>conv. 93%(70%*ee*)<br>54% isolated yield

3.3. ^{1}H-NMR and ^{13}C-NMR spectra of biocatalytic products

Figure S3. ^{1}H-NMR spectra of product ethyl (*R*)-4-phenyl-2-(propargylamino) butyrate hydrochloride ((*R*)-**1a**) of IR271.

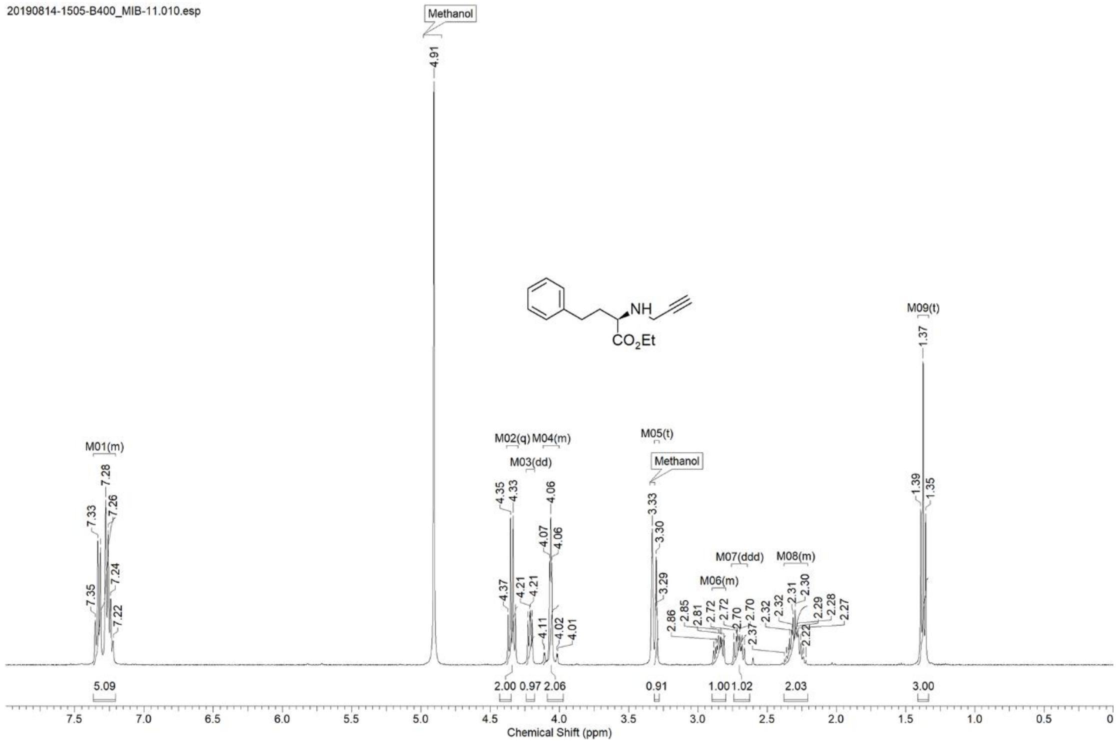

Figure S4. ^{13}C-NMR spectra of product ethyl (*R*)-4-phenyl-2-(propargylamino) butyrate hydrochloride ((*R*)-**1a**) of IR271.

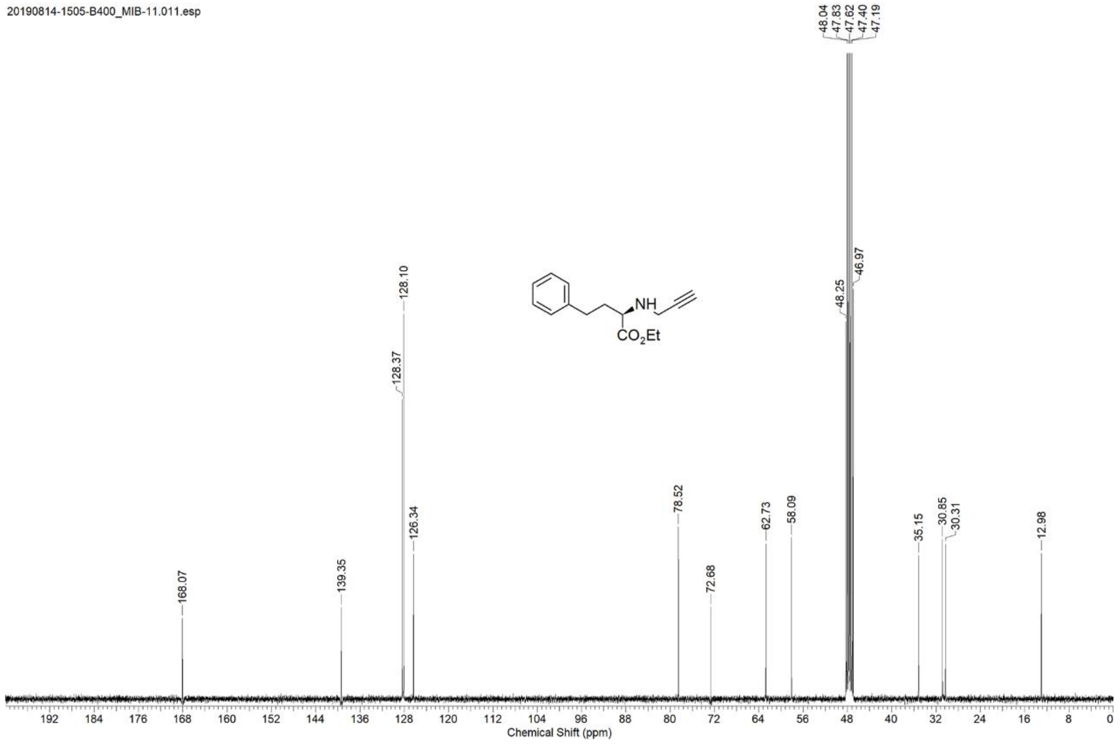

Figure S5. ^{1}H-NMR spectra of product ethyl (*S*)-4-phenyl-2-(propargylamino) butyrate hydrochloride ((*S*)-**1a**) of IR338.

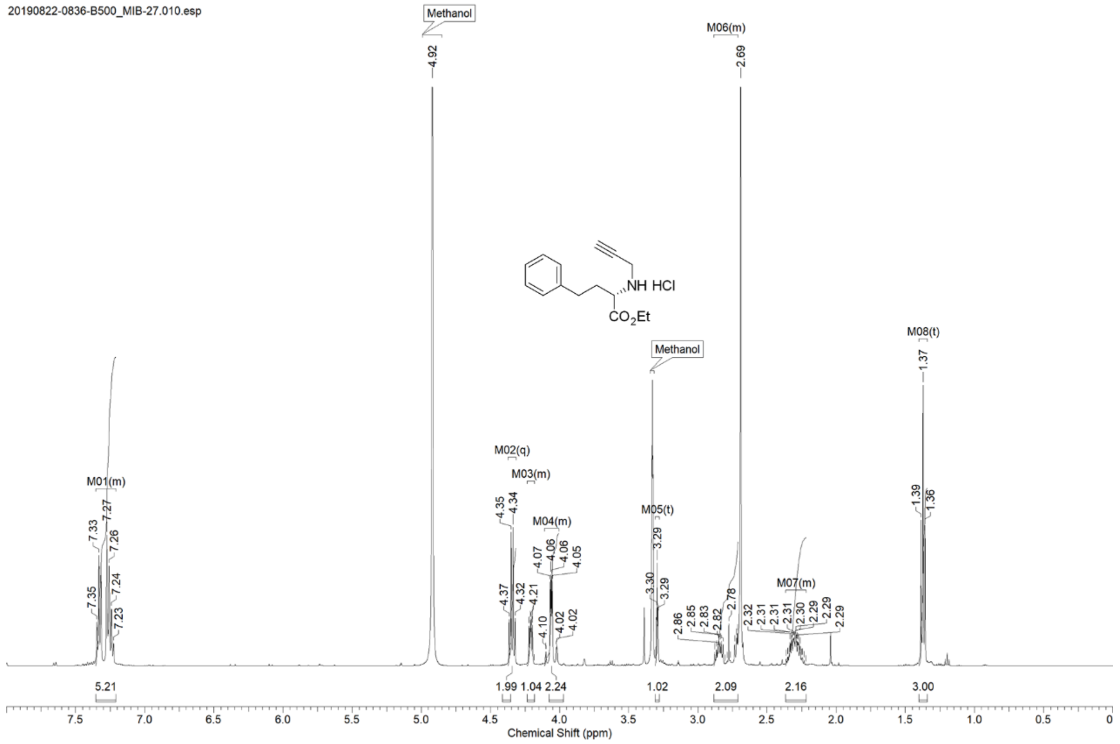

Figure S6. ^{13}C-NMR spectra of product ethyl (*S*)-4-phenyl-2-(propargylamino) butyrate hydrochloride ((*S*)-**1a**) of IR338.

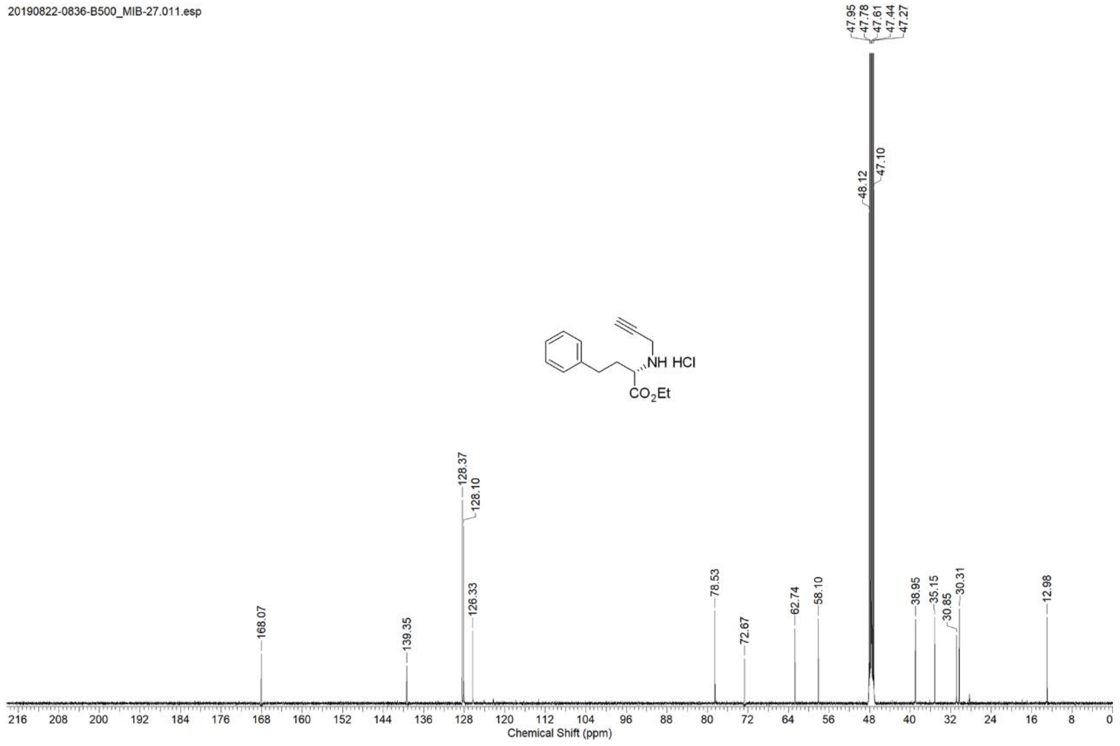

Figure S7. ^{1}H-NMR spectra of product ethyl (*R*)-4-phenyl-2-(methylamino) butyrate hydrochloride ((*R*)-**1b**) of IR271.

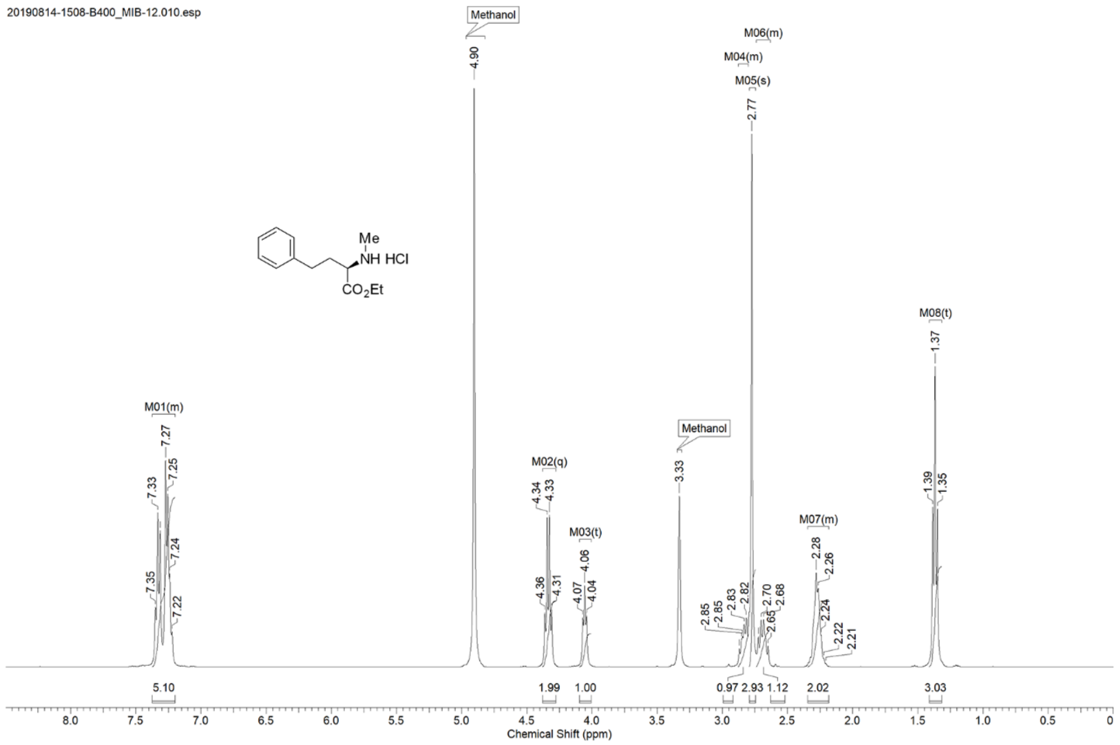

Figure S8. ^{13}C-NMR spectra of product ethyl (*R*)-4-phenyl-2-(methylamino) butyrate hydrochloride ((*R*)-**1b**) of IR271.

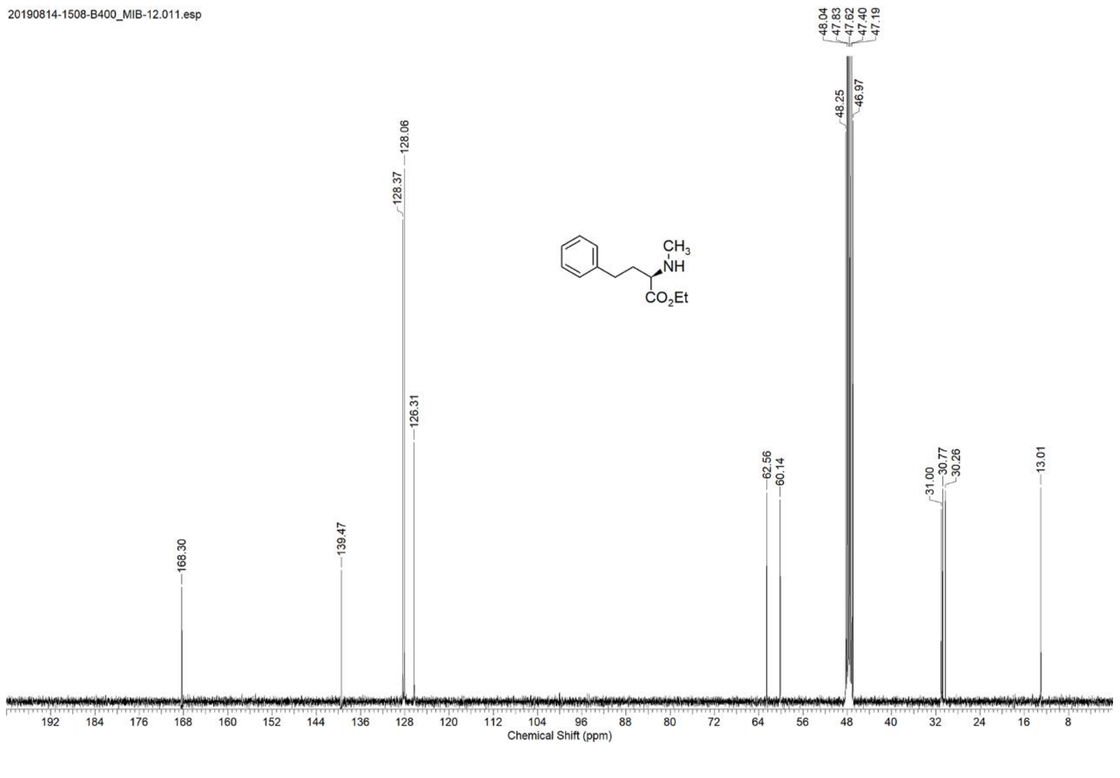

Figure S9. ^{1}H-NMR spectra of product ethyl (*S*)-4-phenyl-2-(methylamino) butyrate hydrochloride ((*S*)-**1b**) of IR338.

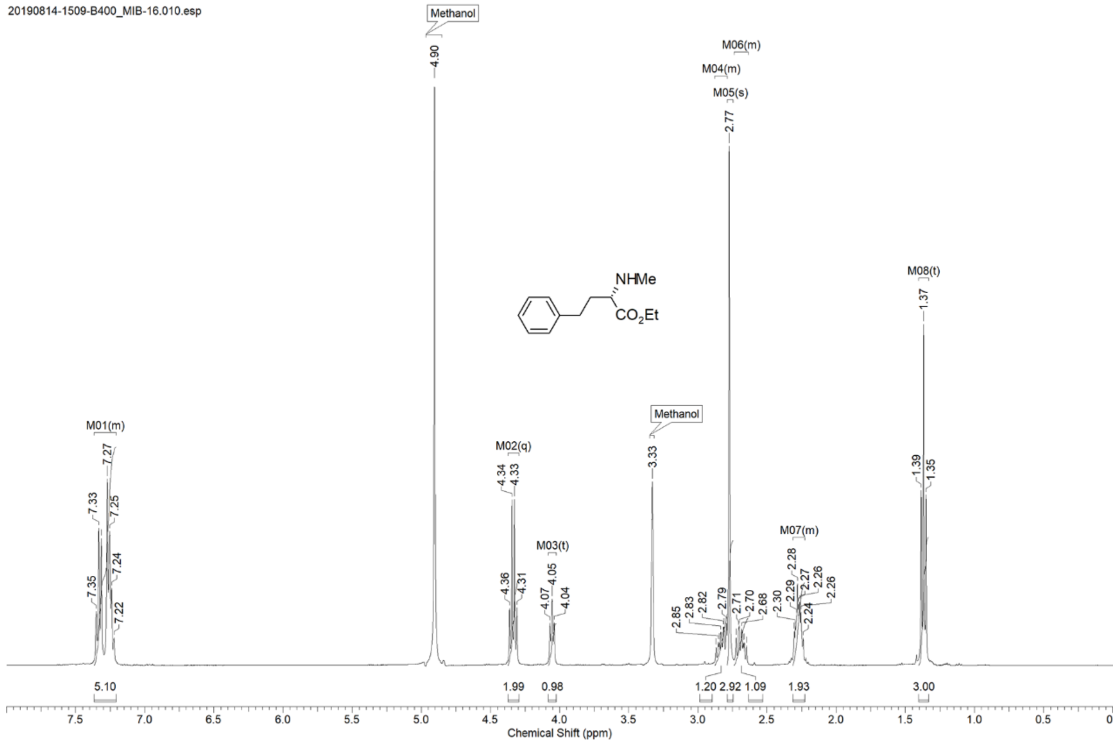

Figure S10. ^{13}C-NMR spectra of product ethyl (*S*)-4-phenyl-2-(methylamino) butyrate hydrochloride ((*S*)-**1b**) of IR338.

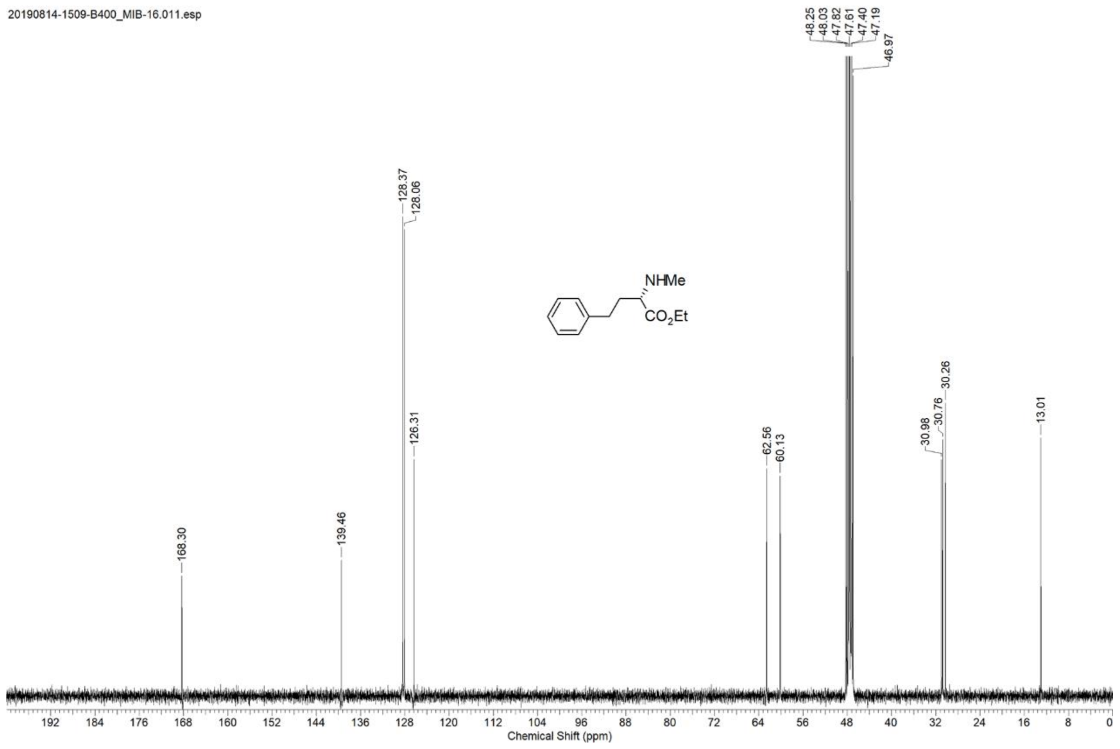

Figure S11. ^{1}H-NMR spectra of product ethyl (*R*)-4-phenyl-2-(propylamino) butyrate hydrochloride ((*R*)-**1c**) of IR271.

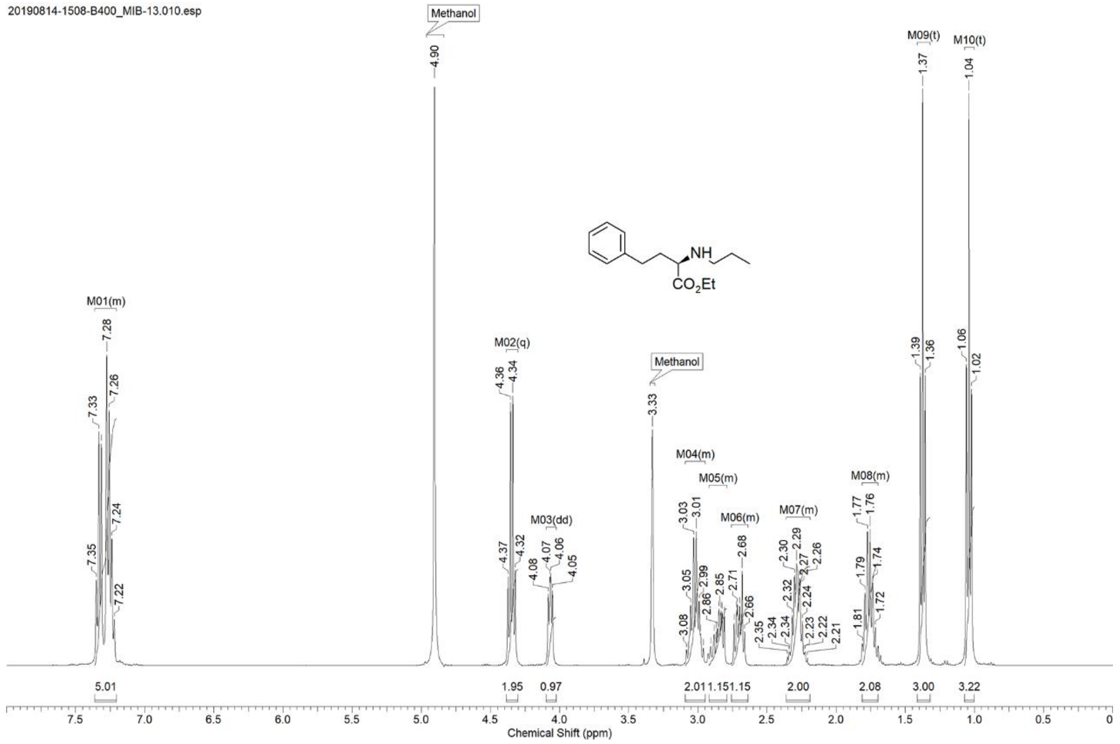

Figure S12. ^{13}C-NMR spectra of product ethyl (*R*)-4-phenyl-2-(propylamino) butyrate hydrochloride ((*R*)-**1c**) of IR271.

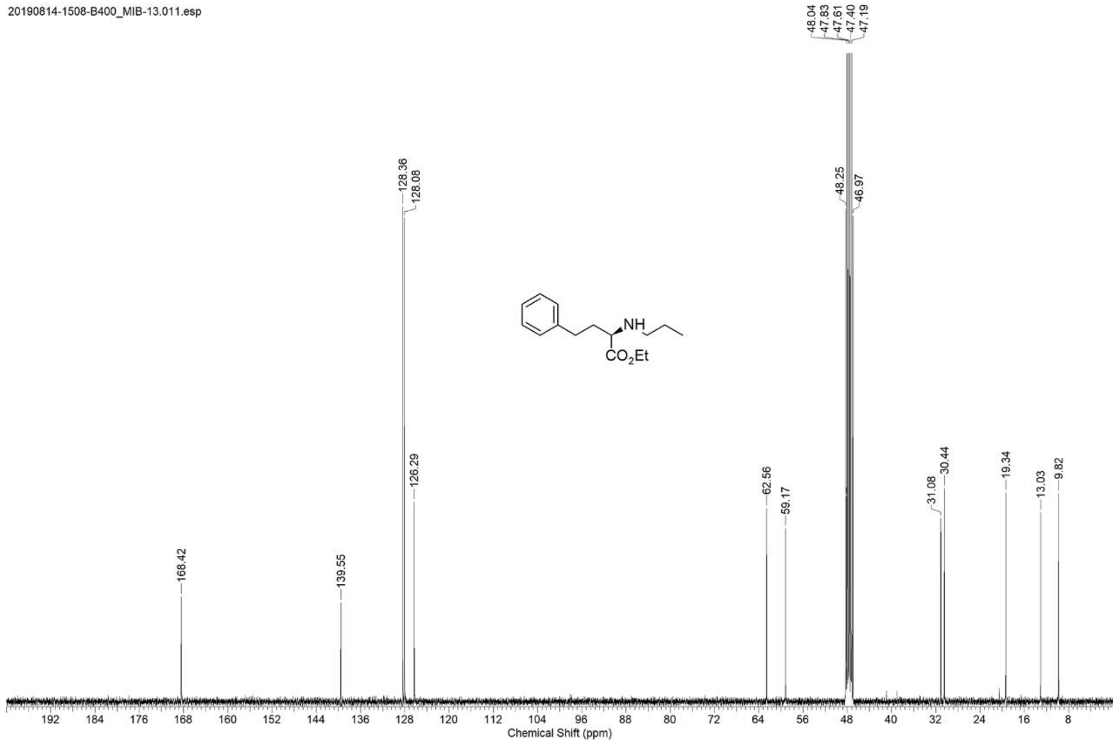

Figure S13. ^{1}H-NMR spectra of product ethyl (*S*)-4-phenyl-2-(propylamino) butyrate hydrochloride ((*S*)-**1c**) of IR338.

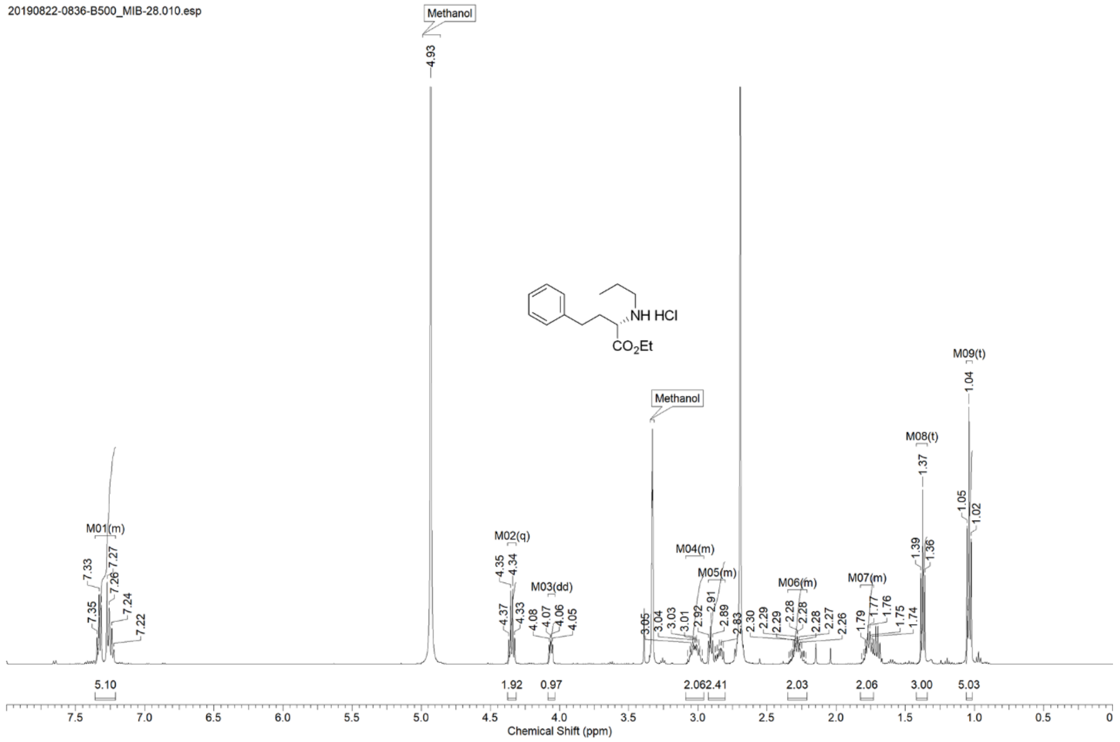

Figure S14. ^{13}C-NMR spectra of product ethyl (*S*)-4-phenyl-2-(propylamino) butyrate hydrochloride ((*S*)-**1c**) of IR338.

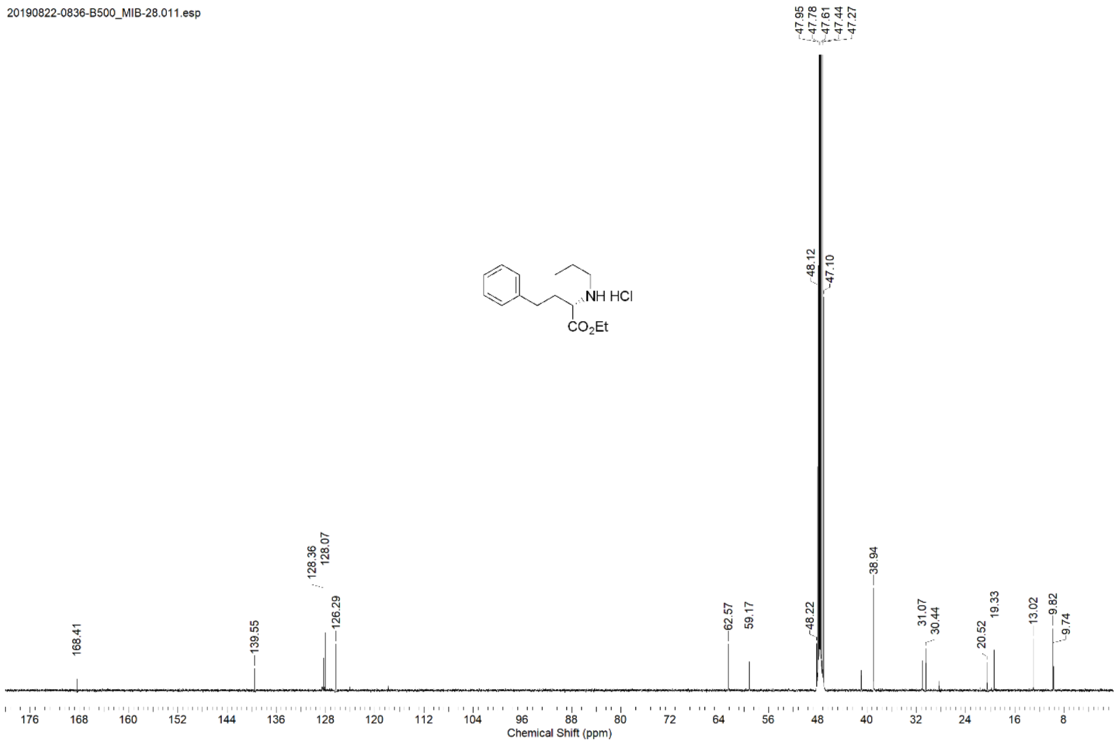

Figure S15. ^{1}H-NMR spectra of product ethyl (*R*)-4-phenyl-2-(allylamino) butyrate hydrochloride ((*R*)-**1d**) of IR271.

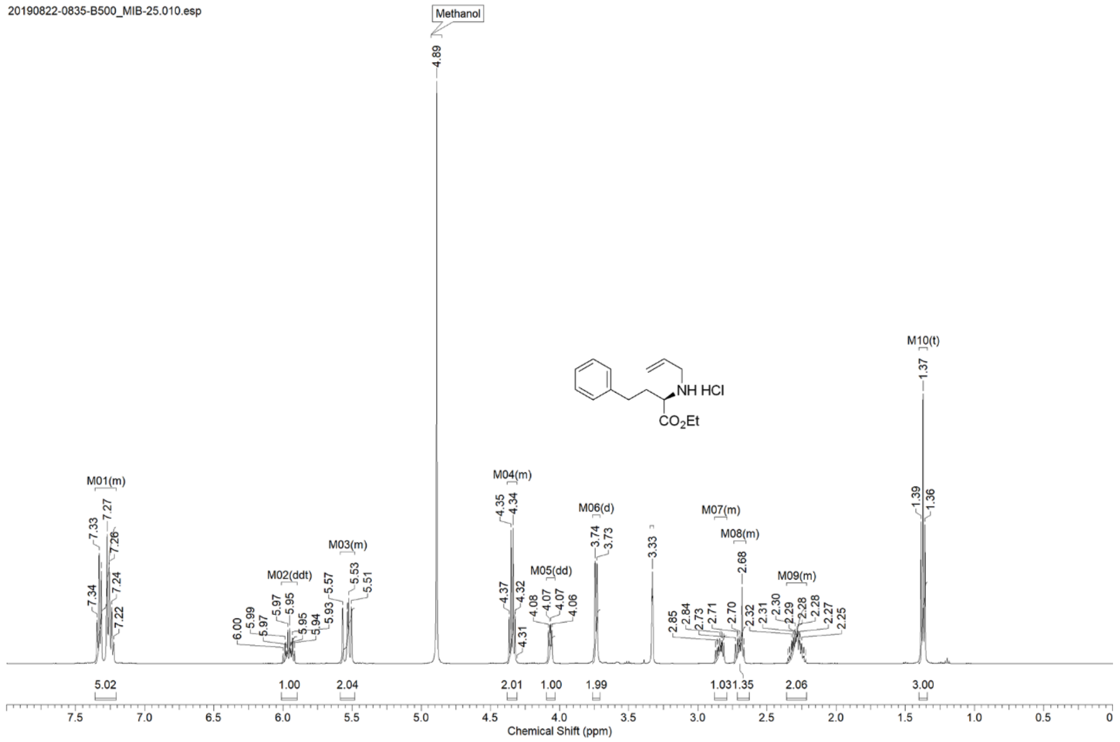

Figure S16. ^{13}C-NMR spectra of product ethyl (*R*)-4-phenyl-2-(allylamino) butyrate hydrochloride ((*R*)-**1d**) of IR271.

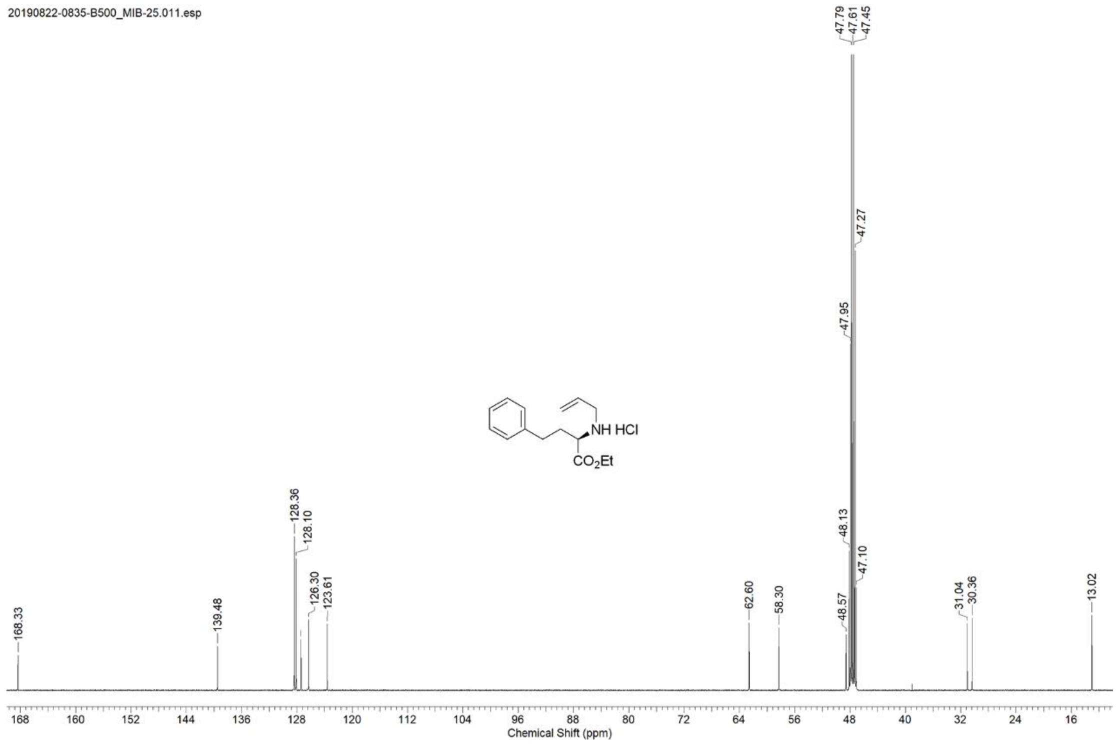

Figure S17. ^{1}H-NMR spectra of product ethyl (*S*)-4-phenyl-2-(allylamino) butyrate hydrochloride ((*S*)-**1d**) of IR338.

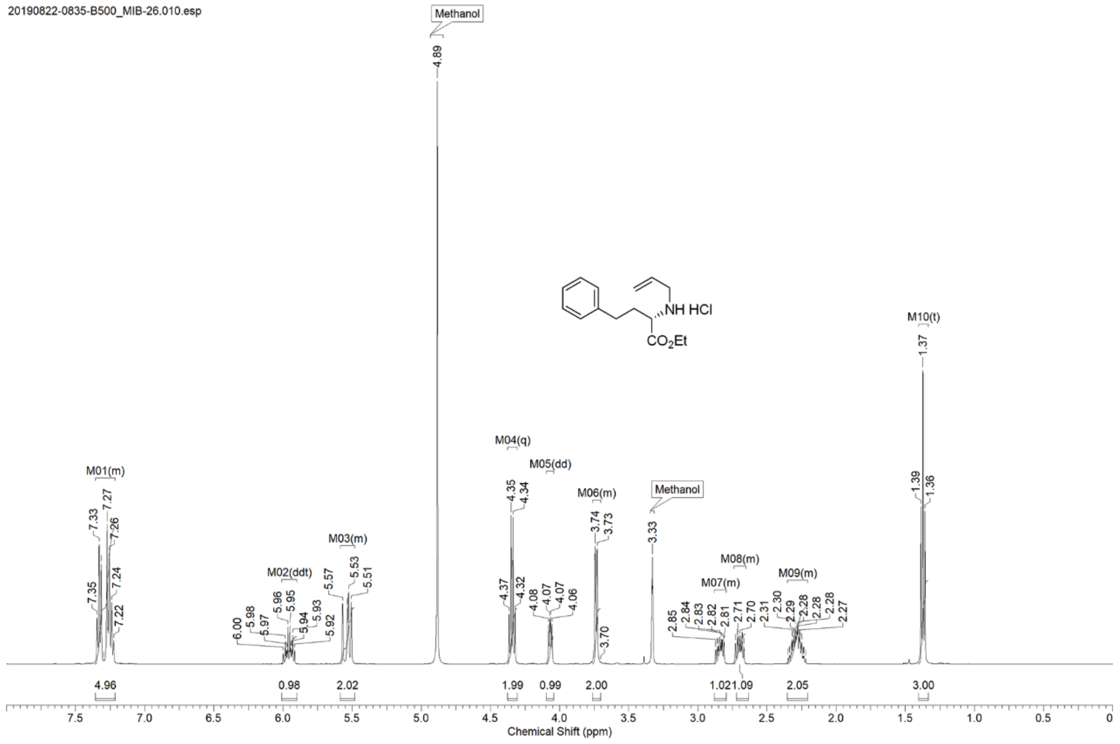

Figure S18. ^{13}C-NMR spectra of product ethyl (*S*)-4-phenyl-2-(allylamino) butyrate hydrochloride ((*S*)-**1d**) of IR338.

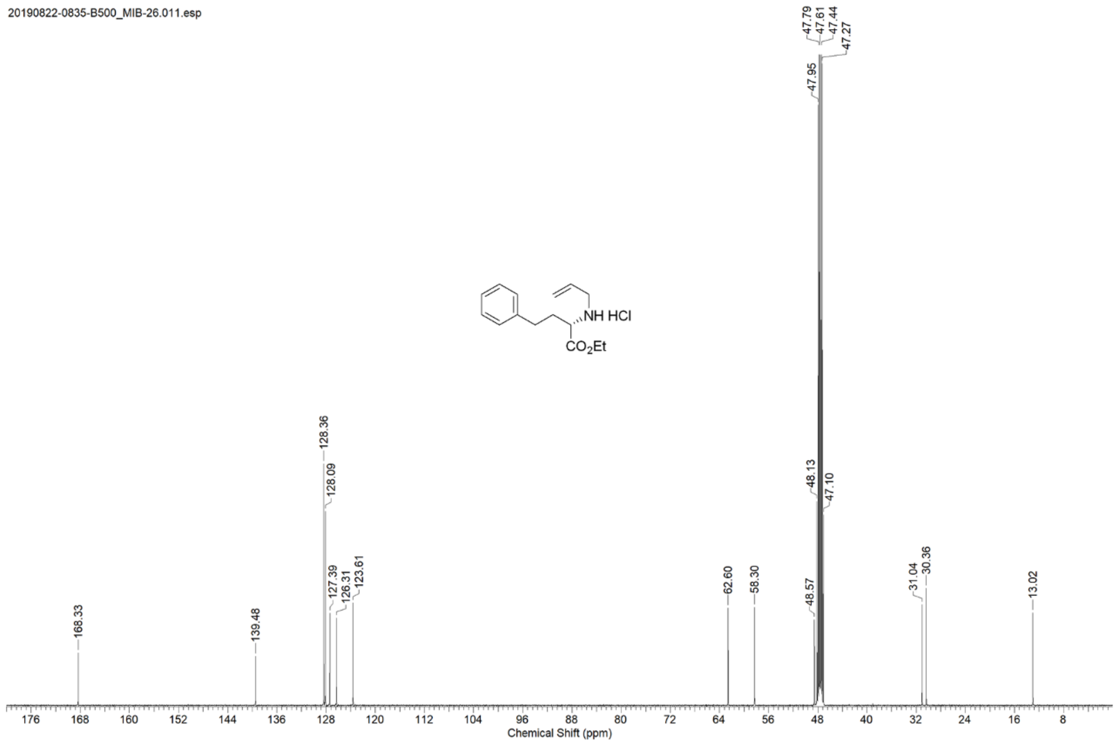

Figure S19. ^{1}H-NMR spectra of product ethyl (*R*)-4-phenyl-2-(cyclopropylamino) butyrate hydrochloride ((*R*)-**1e**) of IR271.

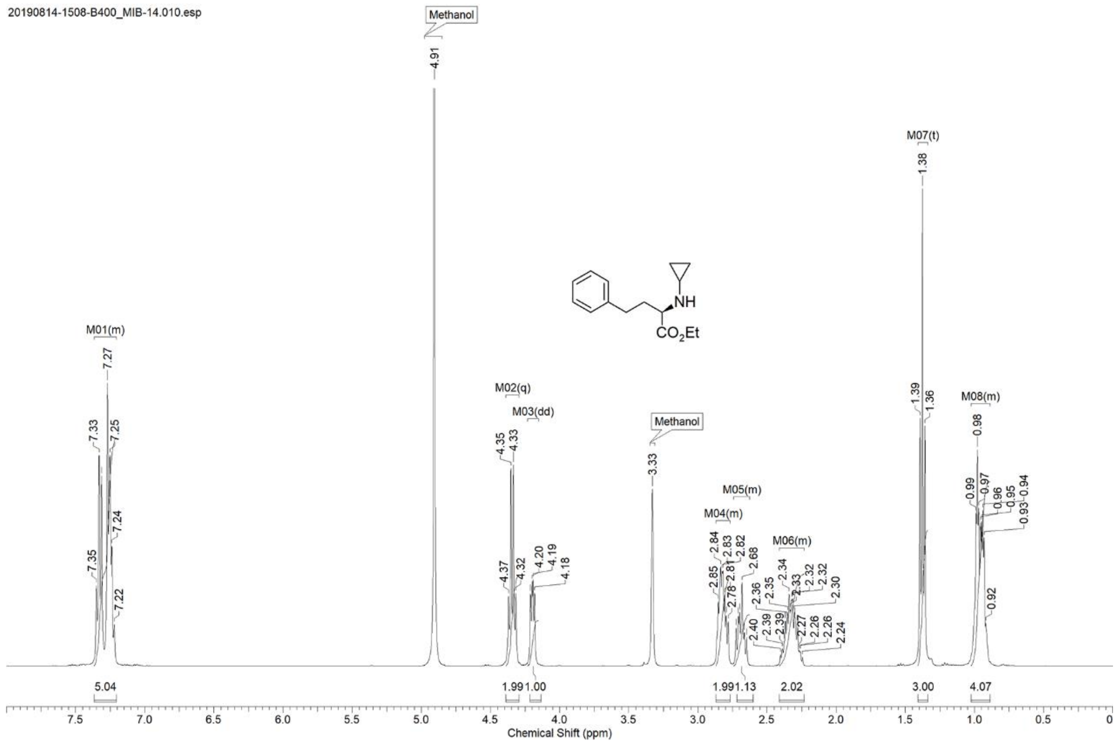

Figure S20. ^{13}C-NMR spectra of product ethyl (*R*)-4-phenyl-2-(cyclopropylamino) butyrate hydrochloride ((*R*)-**1e**) of IR271.

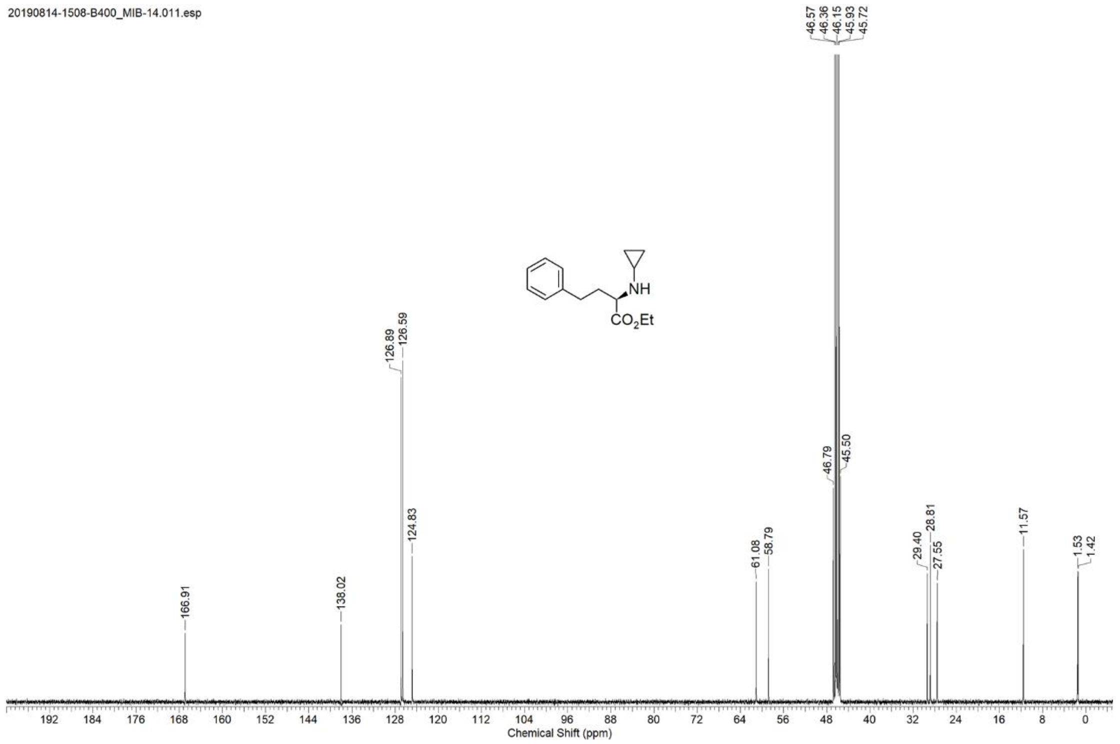

Figure S21. ^{1}H-NMR spectra of product ethyl (*S*)-4-phenyl-2-(cyclopropylamino) butyrate hydrochloride ((*S*)-**1e**) of IR338.

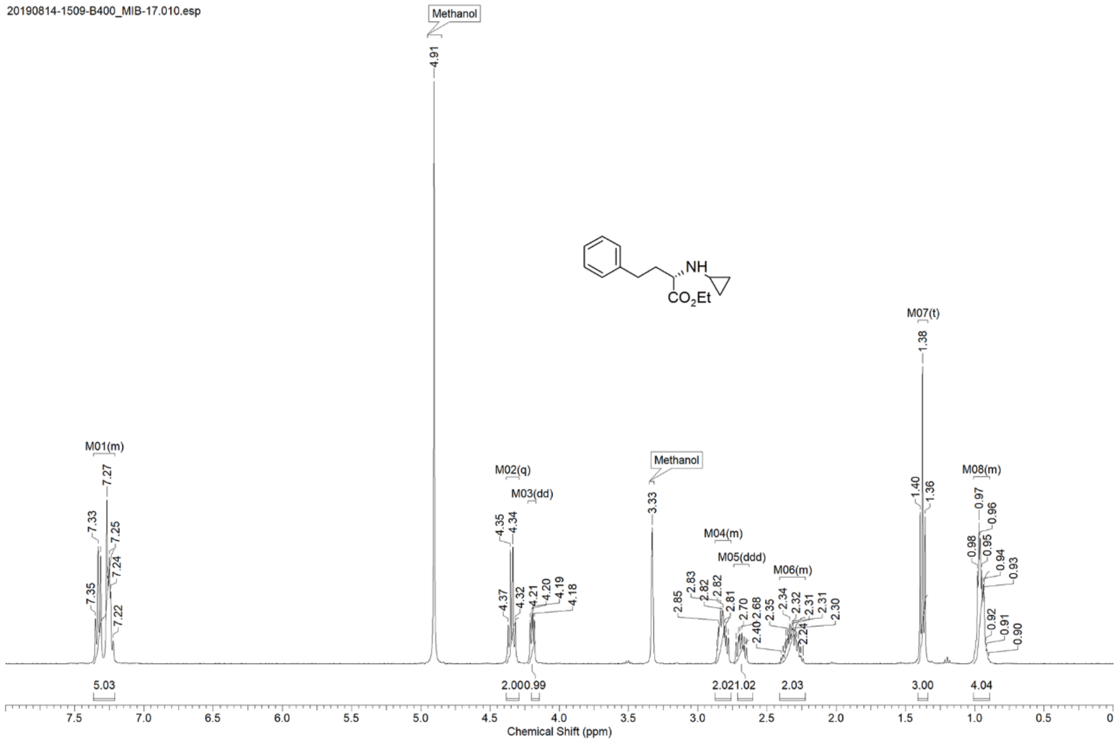

Figure S22. ^{13}C-NMR spectra of product ethyl (*S*)-4-phenyl-2-(cyclopropylamino) butyrate hydrochloride ((*S*)-**1e**) of IR338.

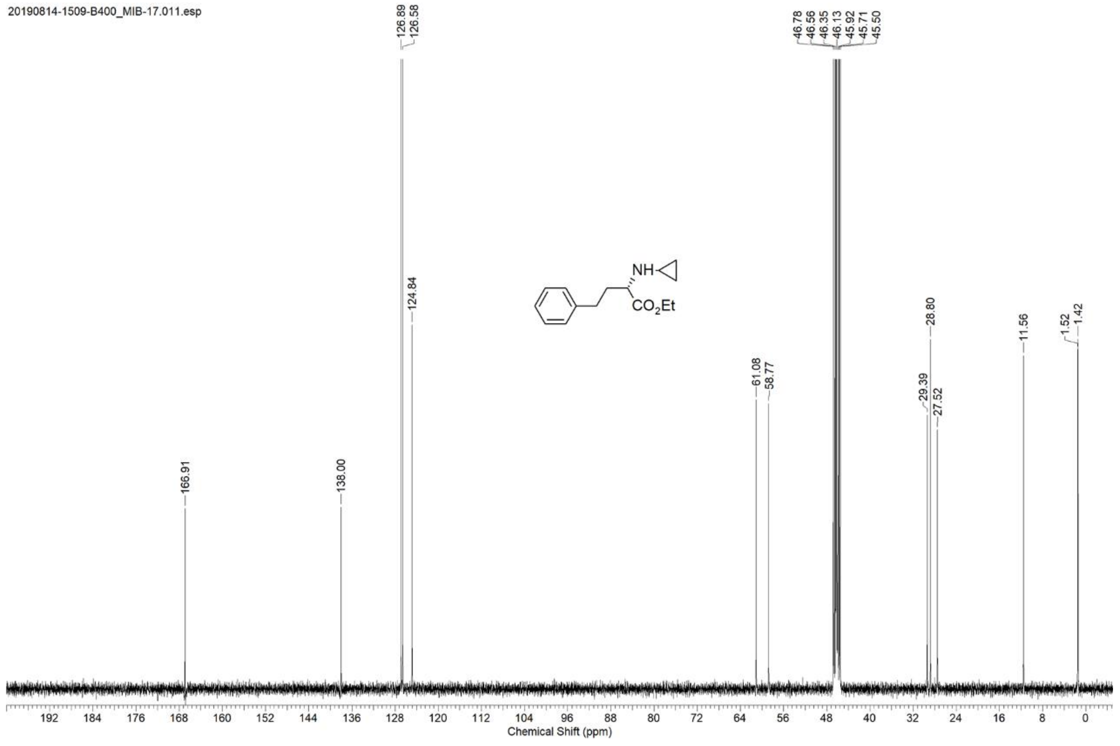

Figure S23. ^{1}H-NMR spectra of product ethyl (*R*)-3-phenyl-2-(propargylamino)propionate hydrochloride ((*R*)-**2a**) of IR271 .

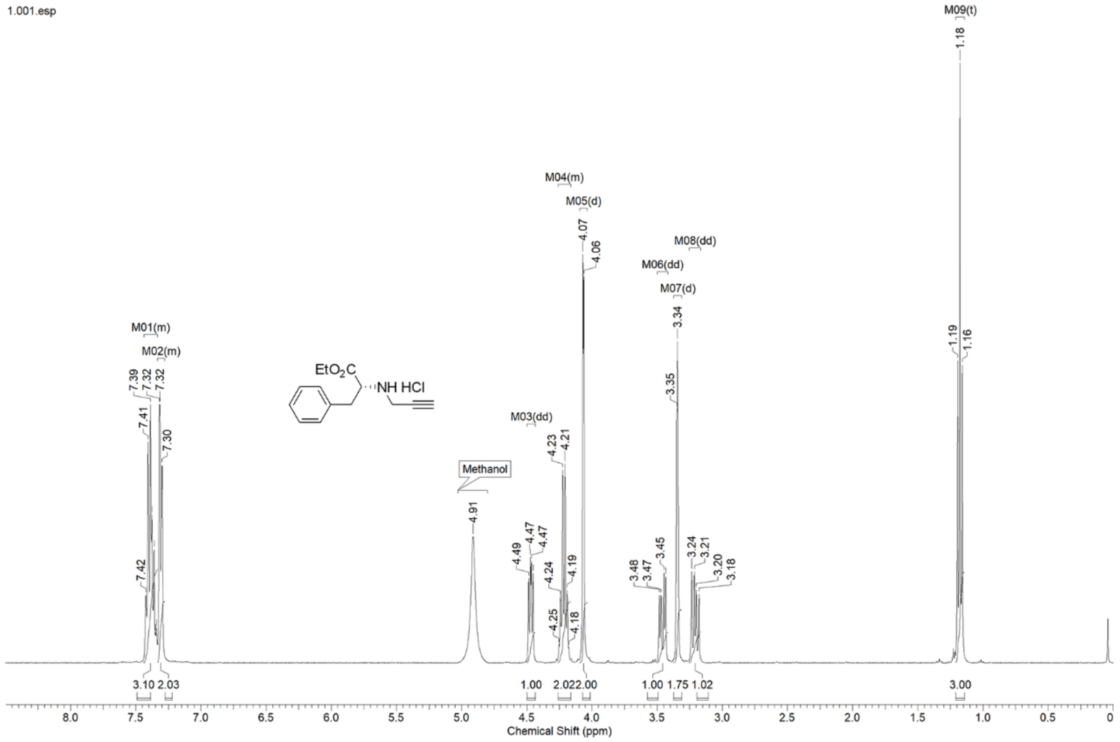

Figure S24. ^{13}C-NMR spectra of product ethyl (*R*)-3-phenyl-2-(propargylamino)propionate hydrochloride ((*R*)-**2a**) of IR271.

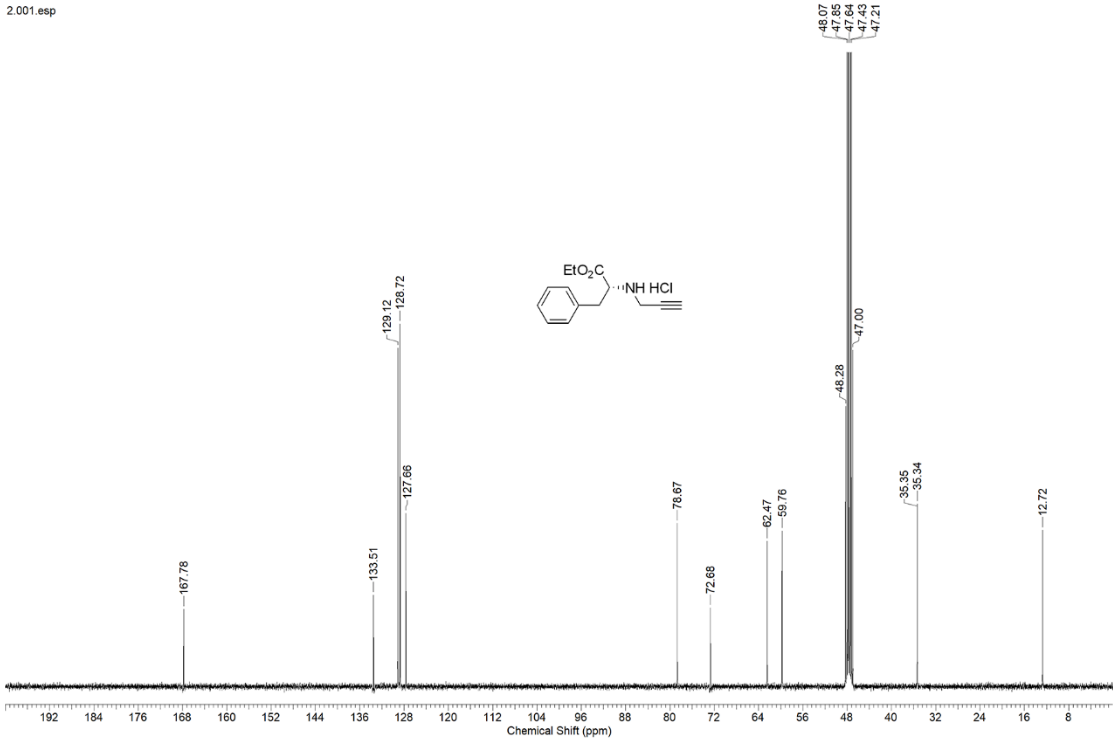

Figure S25. ^{1}H-NMR spectra of product ethyl (*S*)-3-phenyl-2-(propargylamino)propionate hydrochloride ((*S*)-**2a**) of IR355.

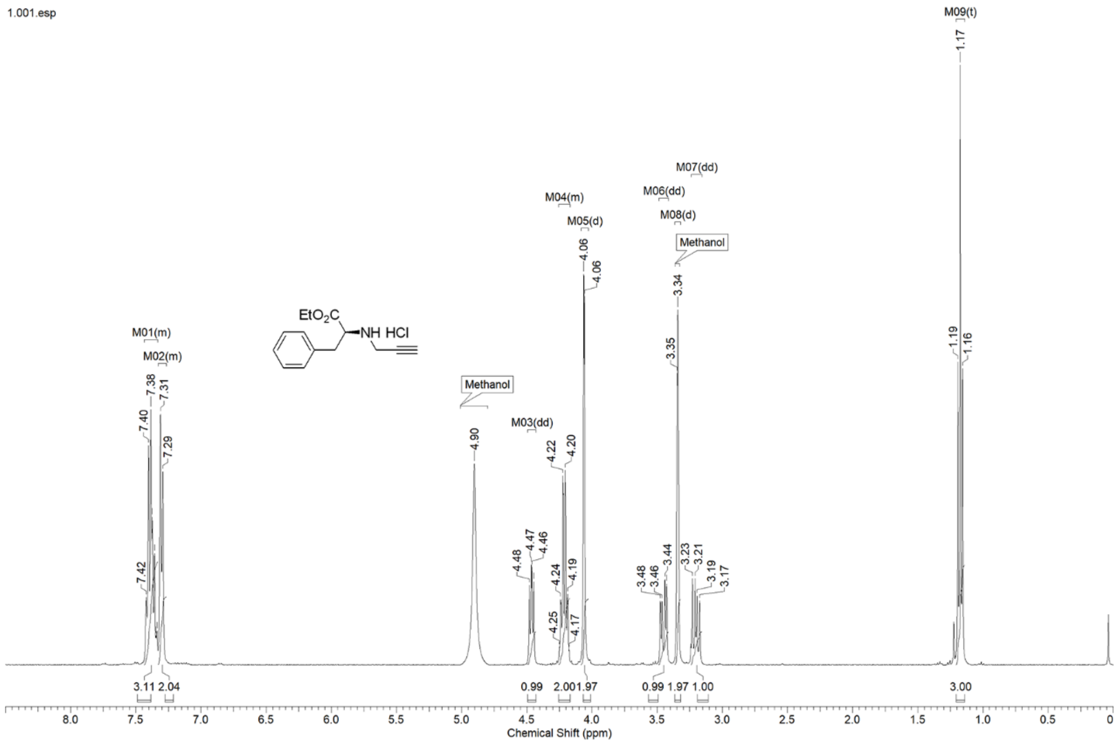

Figure S26. ^{13}C-NMR spectra of product ethyl (*S*)-3-phenyl-2-(propargylamino)propionate hydrochloride ((*S*)-**2a**) of IR355.

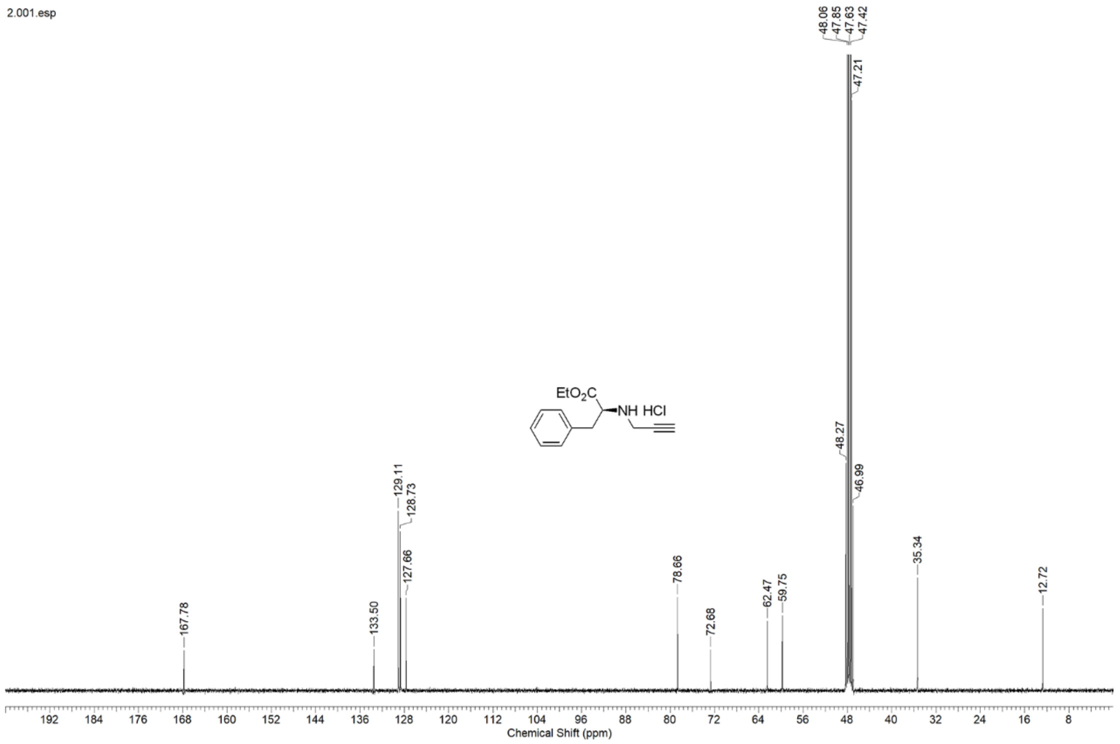

Figure S27. ^{1}H-NMR spectra of product ethyl (*R*)-2-(propargylamino)propionate hydrochloride ((*R*)-**3a**) of IR23.

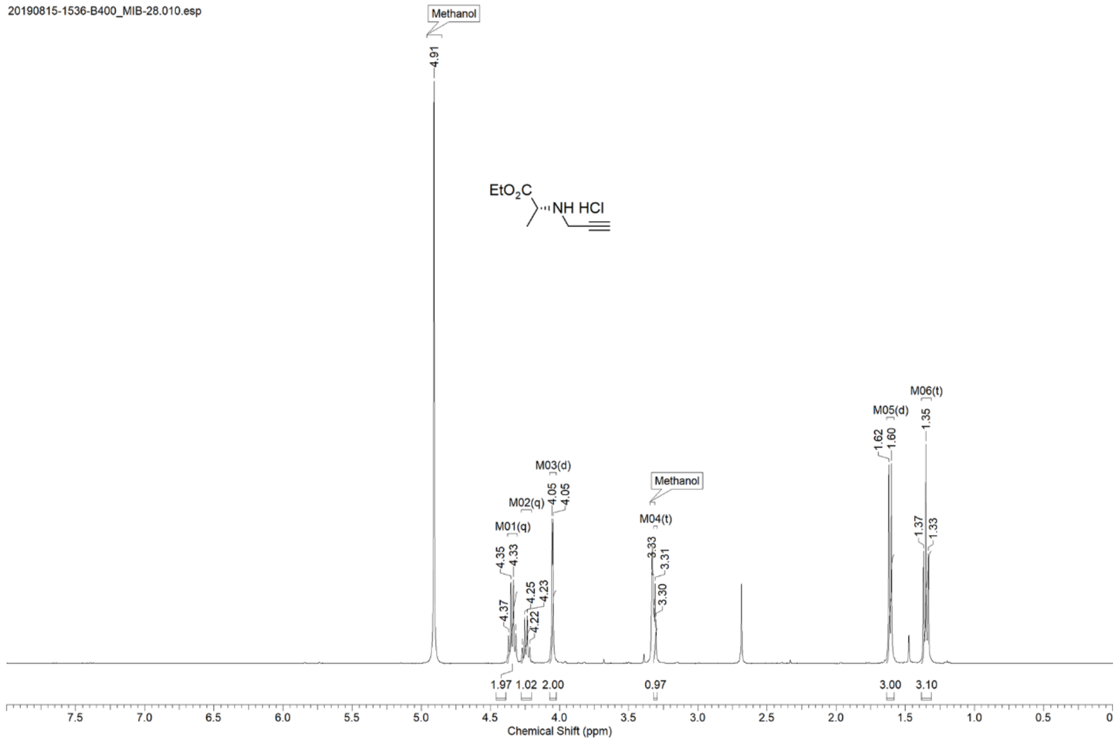

Figure S28. ^{13}C-NMR spectra of product ethyl (*R*)-2-(propargylamino)propionate hydrochloride ((*R*)-**3a**) of IR23.

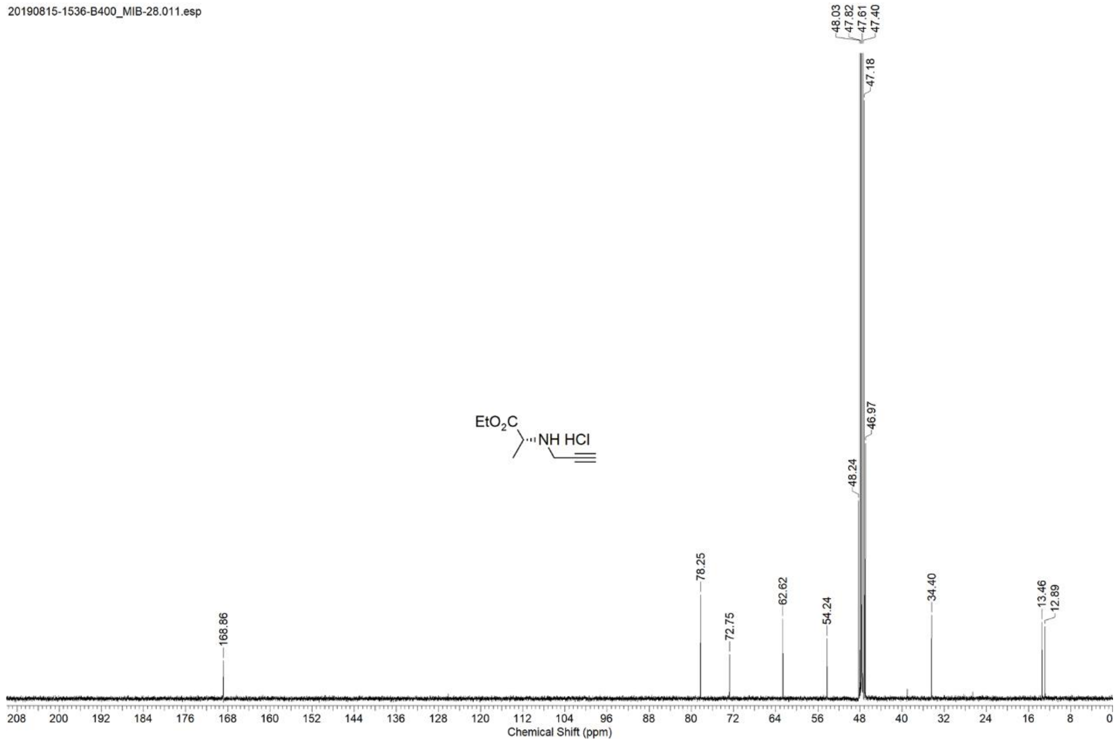

SUPPORTING INFORMATION

Figure S29. ^{1}H-NMR spectra of product ethyl (*S*)-2-(propargylamino)propionate hydrochloride ((*S*)-**3a**) of IR125.

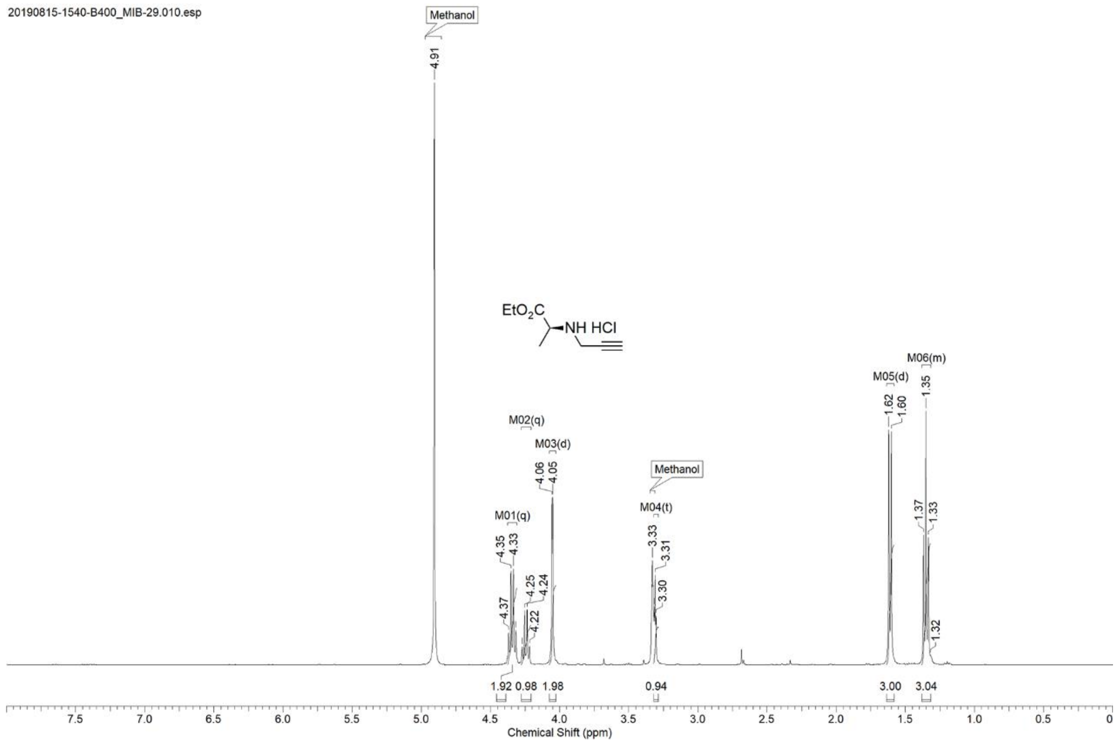

Figure S30. ^{13}C-NMR spectra of product ethyl (*S*)-2-(propargylamino)propionate hydrochloride ((*S*)-**3a**) of IR125.

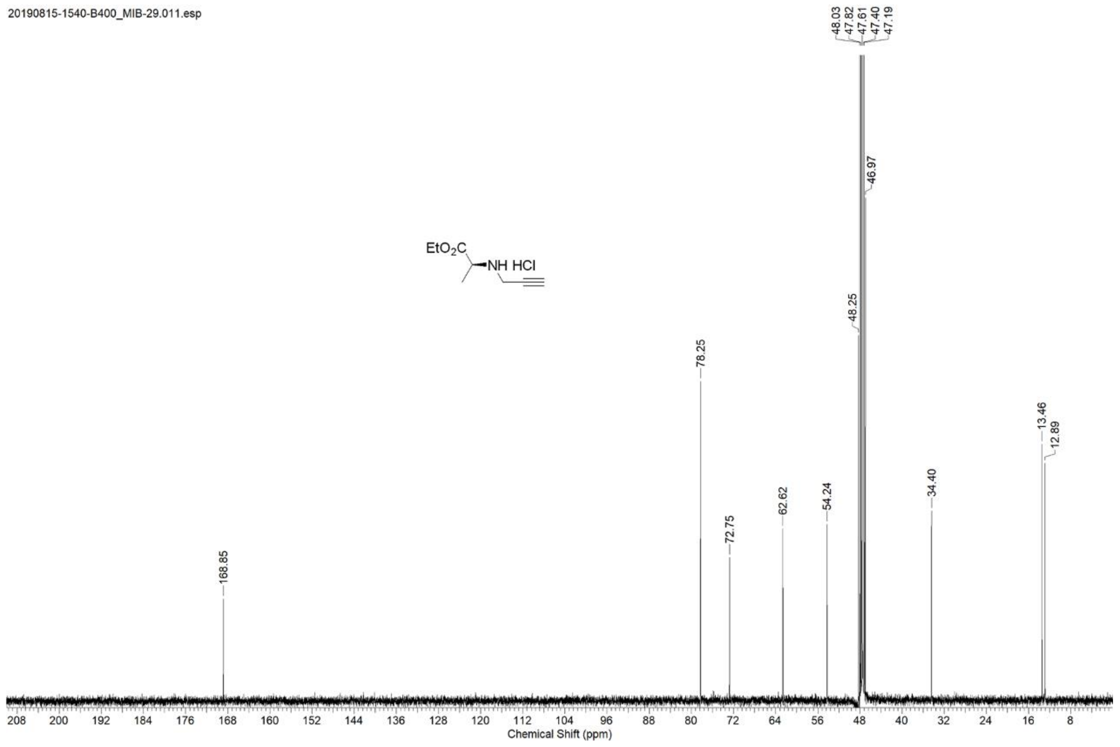

Figure S31. ^{1}H-NMR spectra of product ethyl (*R*)-2-(propargylamino)valerate hydrochloride ((*R*)-**4a**) of IR271.

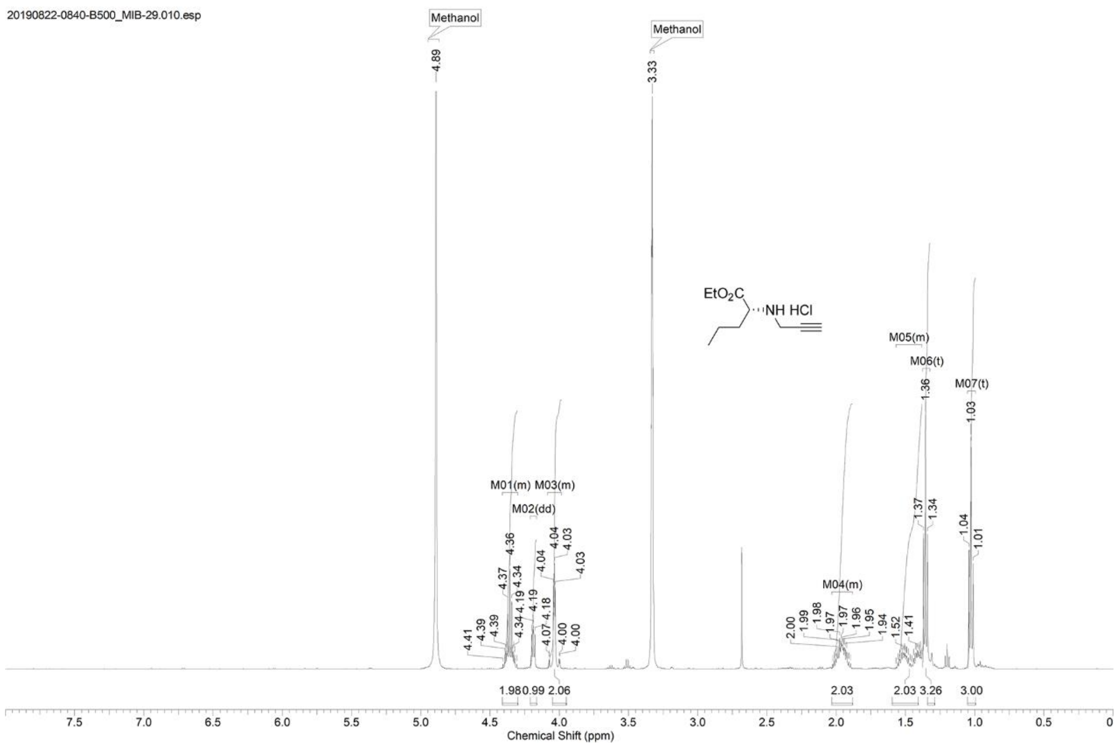

Figure S32. ^{13}C-NMR spectra of product ethyl (*R*)-2-(propargylamino)valerate hydrochloride ((*R*)-**4a**) of IR271.

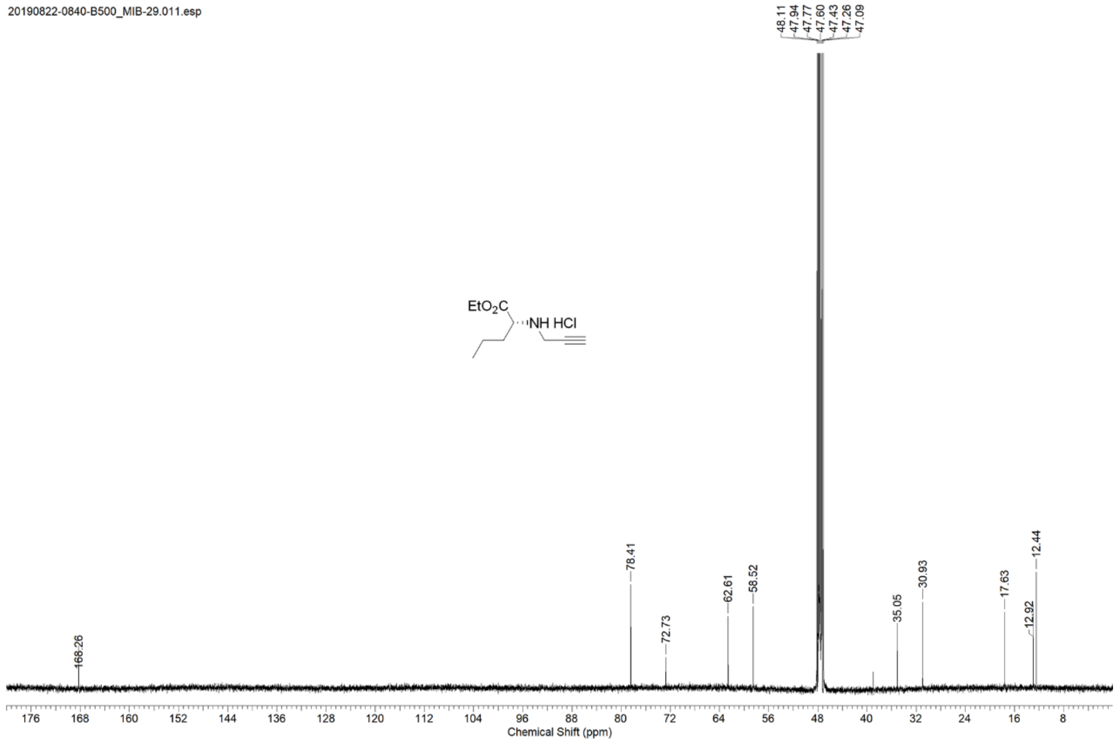

Figure S33. ^{1}H-NMR spectra of product ethyl (*S*)-2-(propargylamino)valerate hydrochloride ((*S*)-**4a**) of IR358.

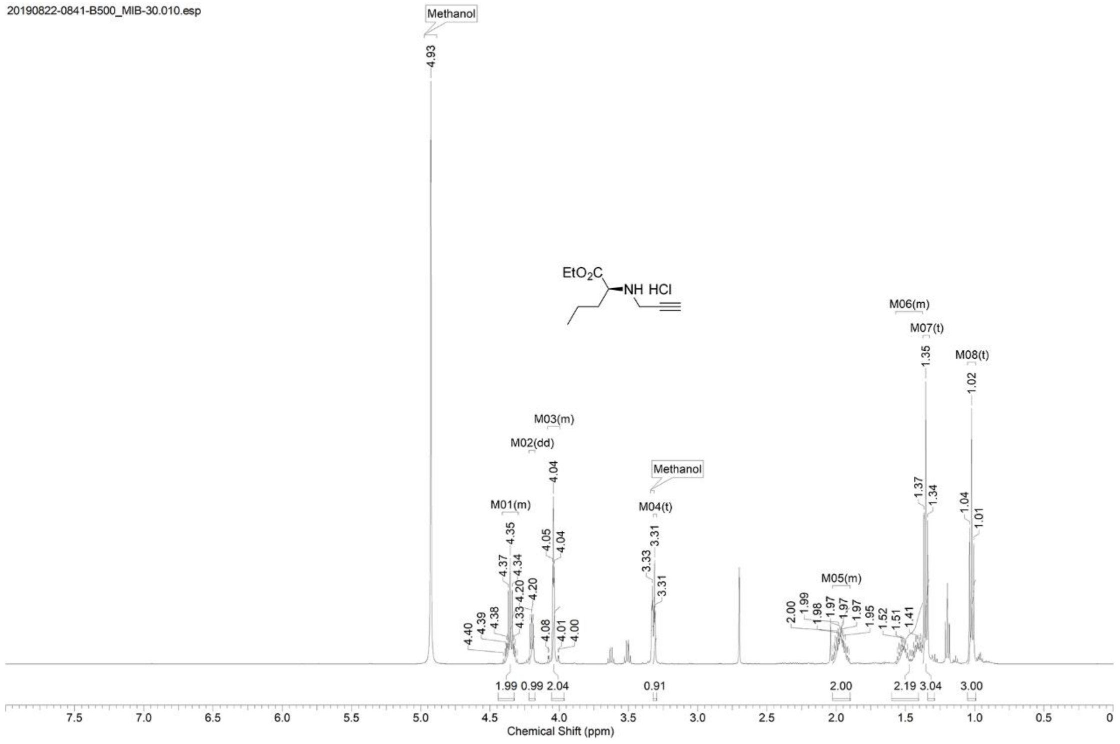

Figure S34. ^{13}C-NMR spectra of product ethyl (*S*)-2-(propargylamino)valerate hydrochloride ((*S*)-**4a**) of IR358.

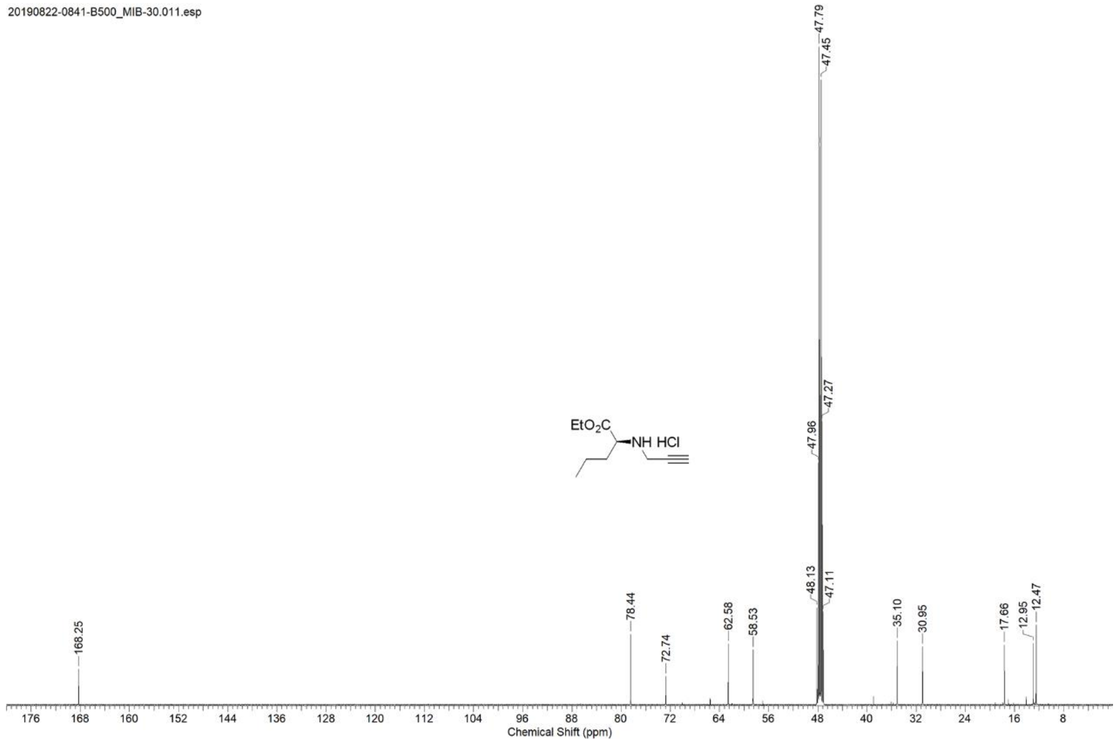

Figure S35. ^{1}H-NMR spectra of product ethyl (*R*)-4-methyl-2-(propargylamino) valerate hydrochloride ((*R*)-**5a**) of IR271.

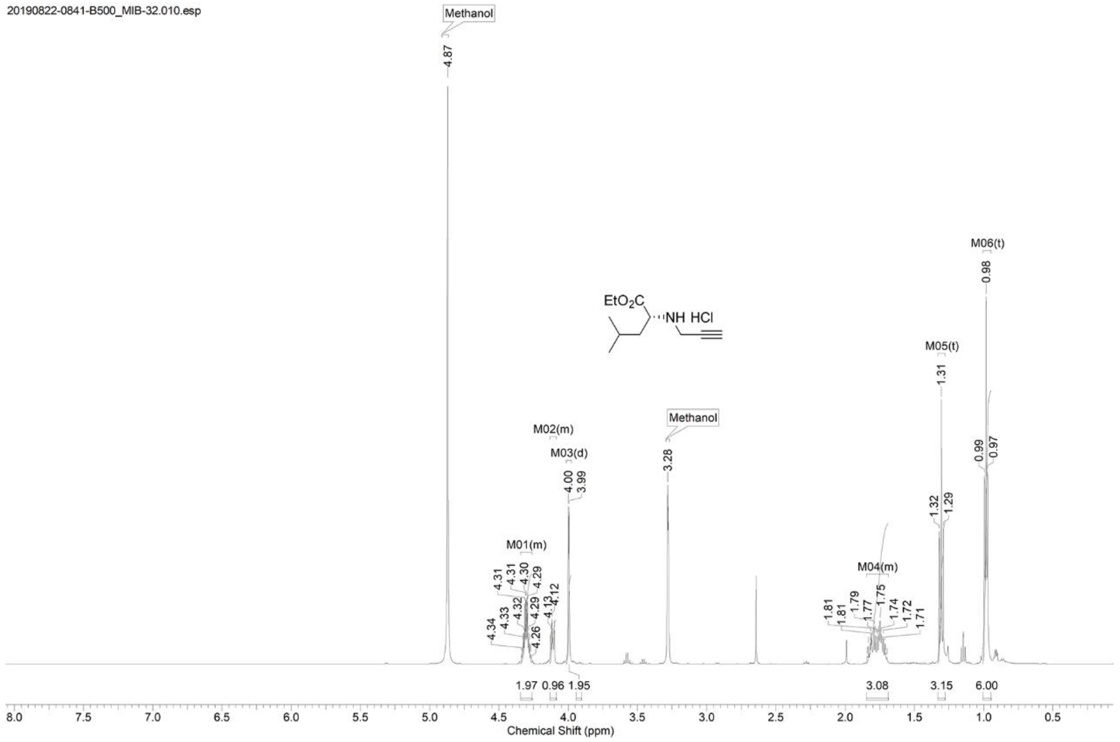

Figure S36. ^{13}C-NMR spectra of product ethyl (*R*)-4-methyl-2-(propargylamino) valerate hydrochloride ((*R*)-**5a**) of IR271.

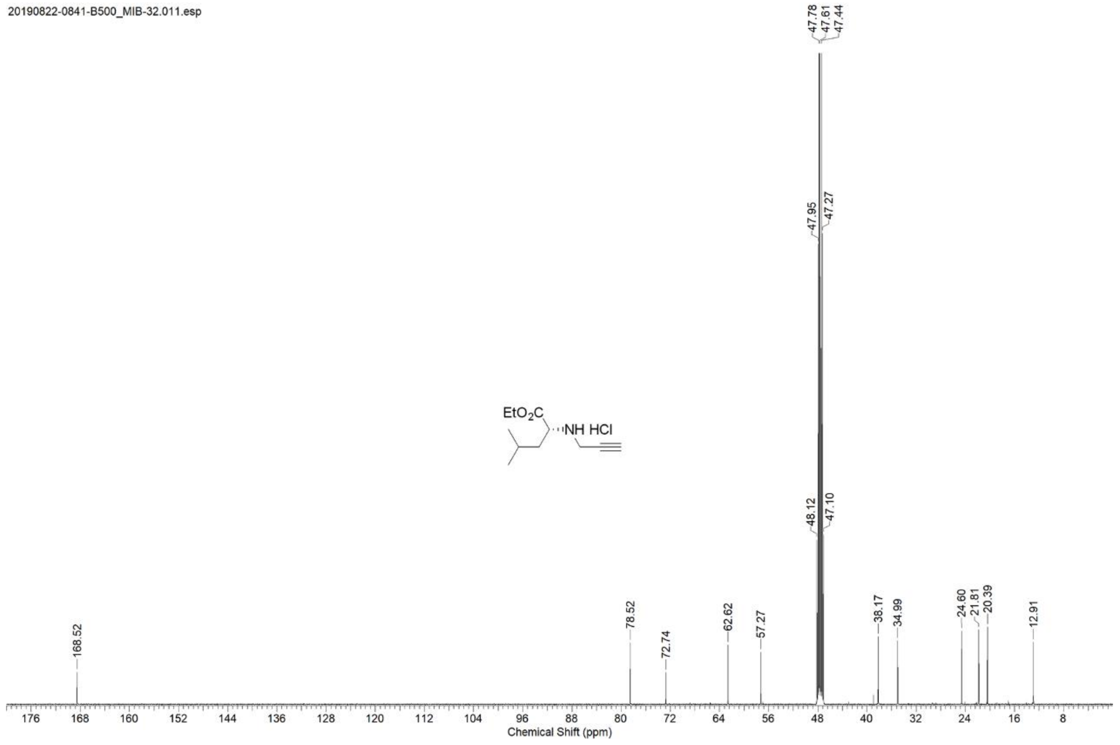

Figure S37. ^{1}H-NMR spectra of product ethyl (*S*)-4-methyl-2-(propargylamino) valerate hydrochloride ((*S*)-**5a**) of IR358.

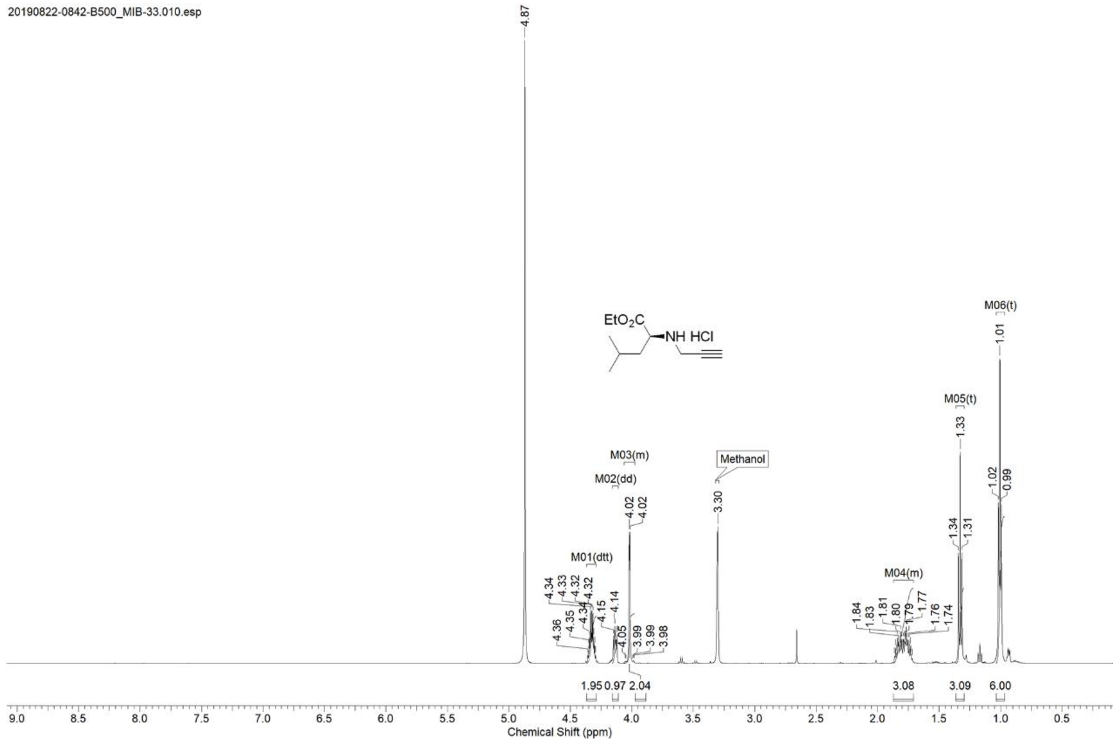

Figure S38. ^{13}C-NMR spectra of product ethyl (*S*)-4-methyl-2-(propargylamino) valerate hydrochloride ((*S*)-**5a**) of IR358.

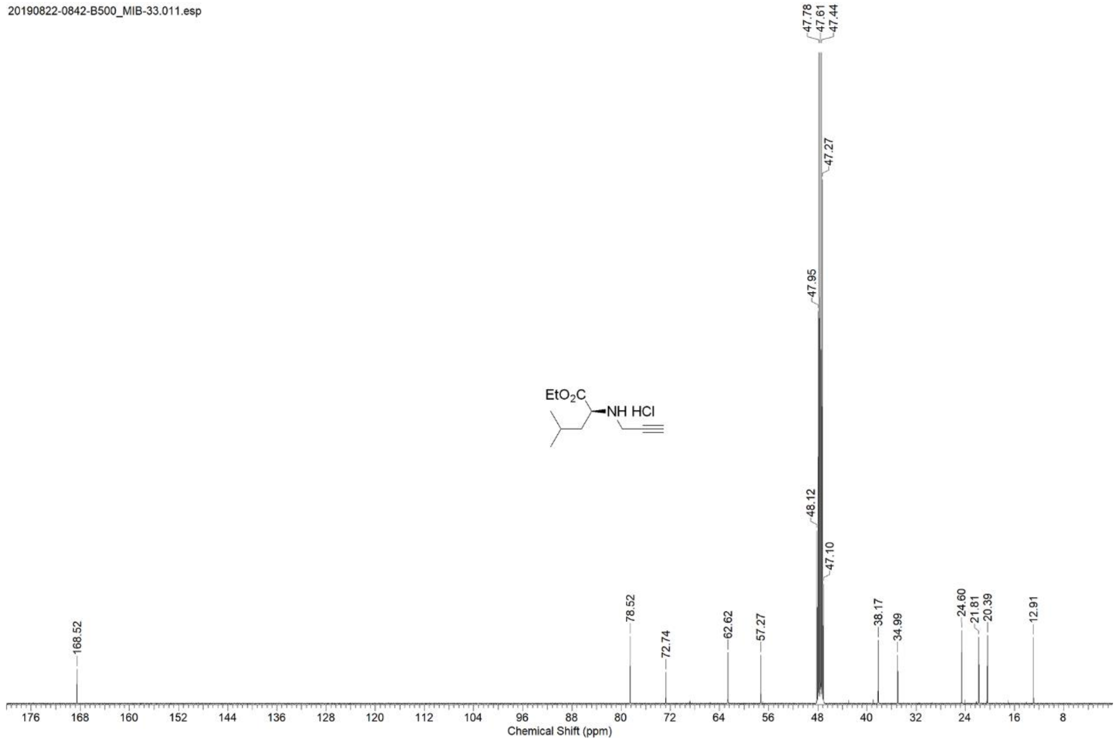

Figure S39. ^{1}H-NMR spectra of product ethyl (*R*)-2-(propargylamino)heptanoate hydrochloride ((*R*)-**6a**) of IR271.

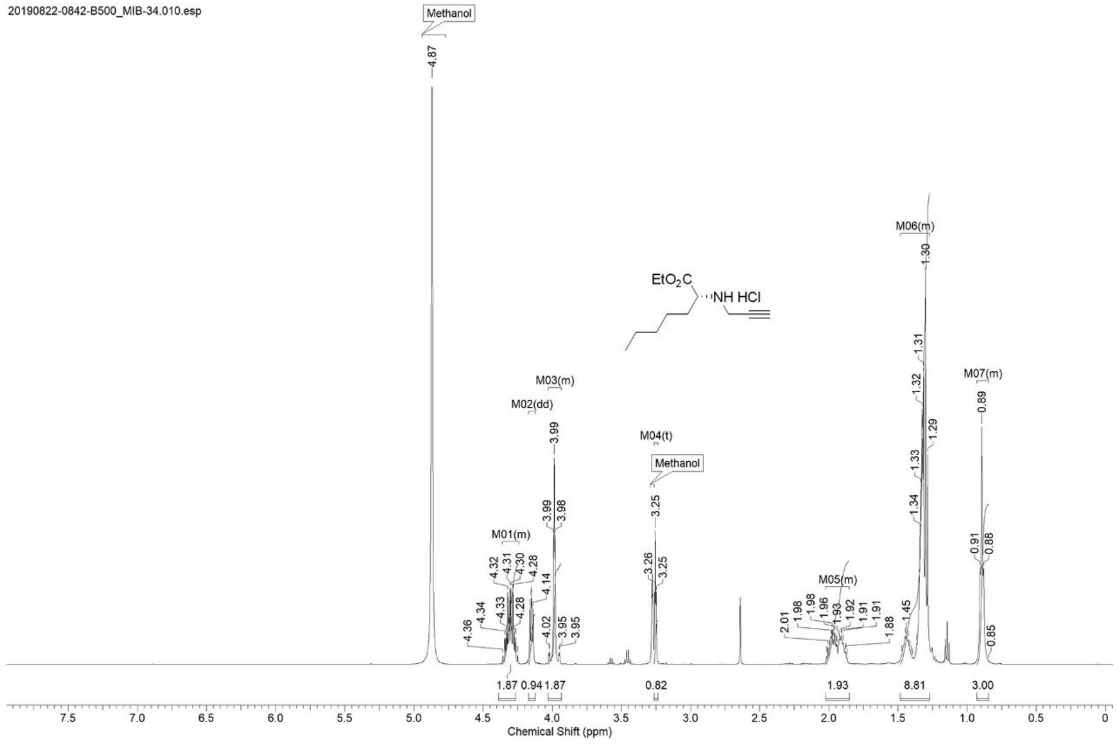

Figure S40. ^{13}C-NMR spectra of product ethyl (*R*)-2-(propargylamino)heptanoate hydrochloride ((*R*)-**6a**) of IR271.

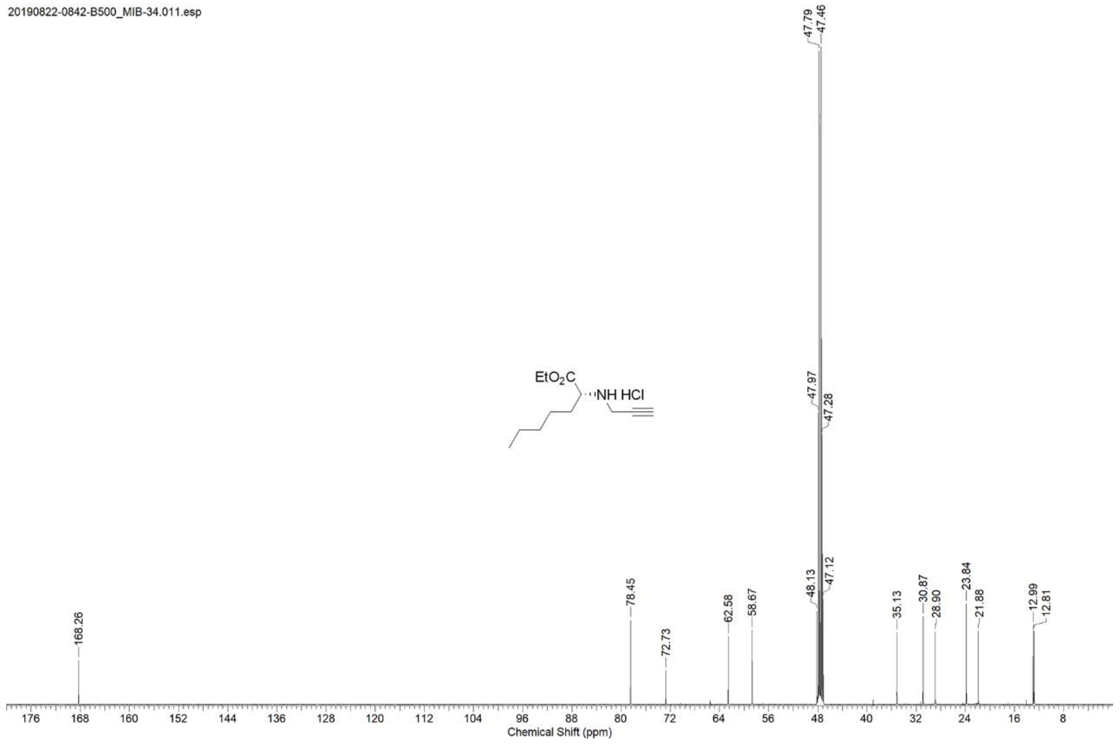

Figure S41. ^{1}H-NMR spectra of product ethyl (*S*)-2-(propargylamino)heptanoate hydrochloride ((*S*)-**6a**) of IR355.

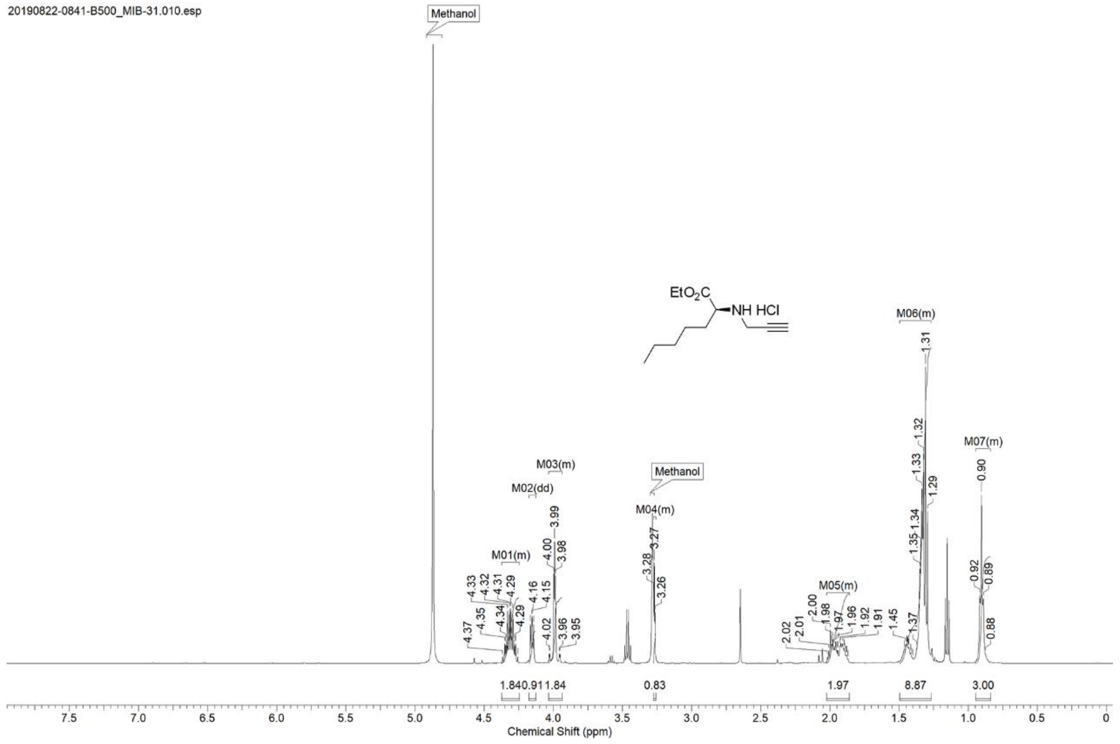

Figure S42. ^{13}C-NMR spectra of product ethyl (*S*)-2-(propargylamino)heptanoate hydrochloride ((*S*)-**6a**) of IR355.

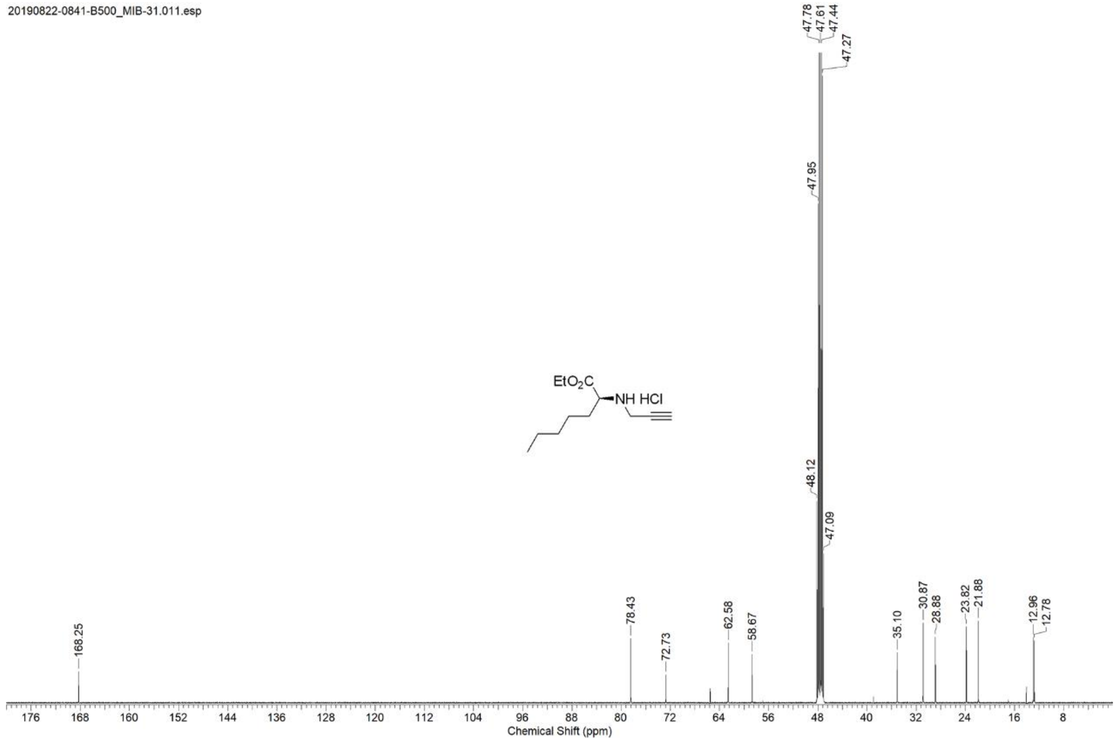

Figure S43. ^{1}H-NMR spectra of product ethyl (*R*)-2-(methylamino)heptanoate hydrochloride ((*R*)-**6b**) of IR271.

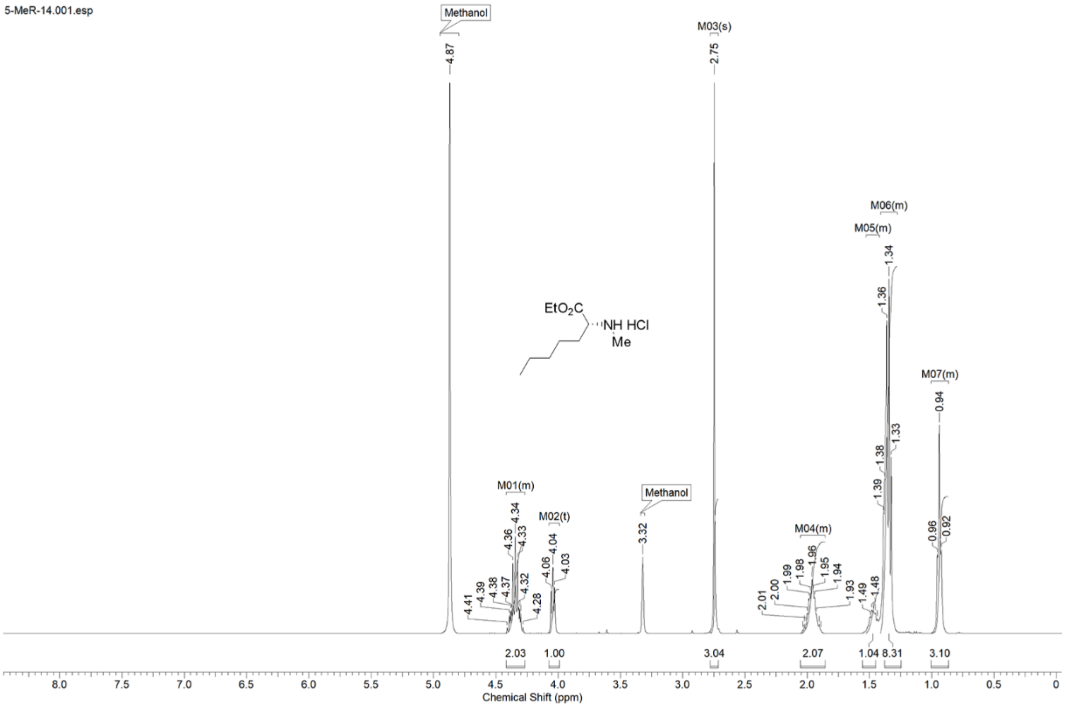

Figure S44. ^{13}C-NMR spectra of product ethyl (*R*)-2-(methylamino)heptanoate hydrochloride ((*R*)-**6b**) of IR271.

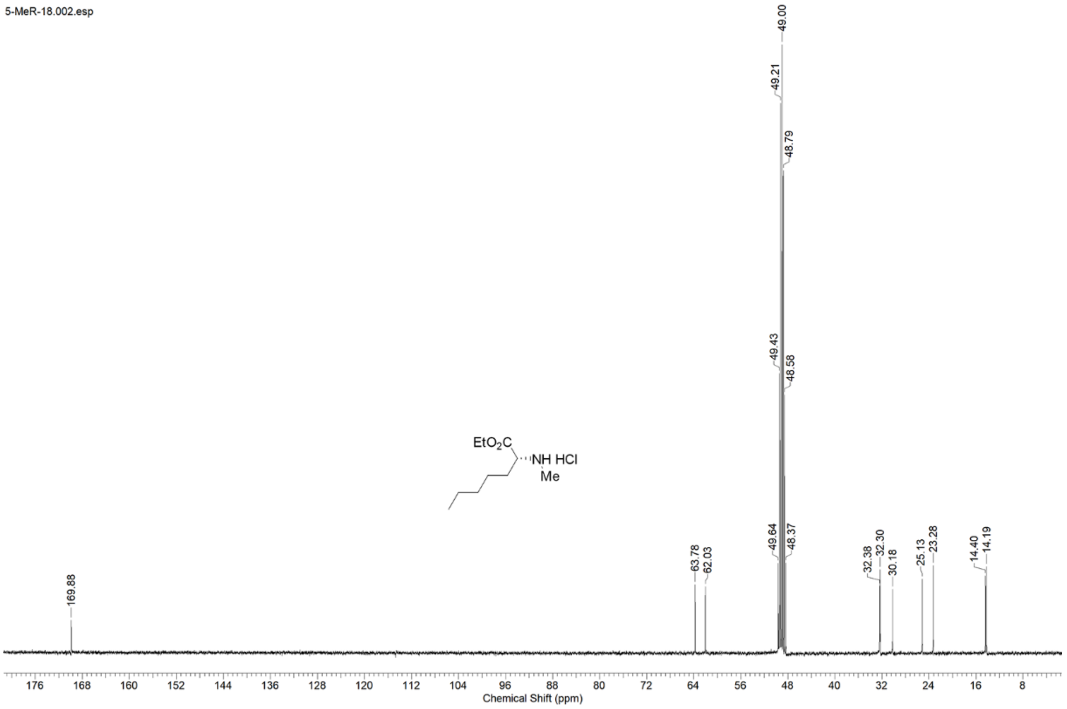

Figure S45. ^{1}H-NMR spectra of product ethyl (*S*)-2-(methylamino)heptanoate hydrochloride ((*S*)-**6b**) of IR358.

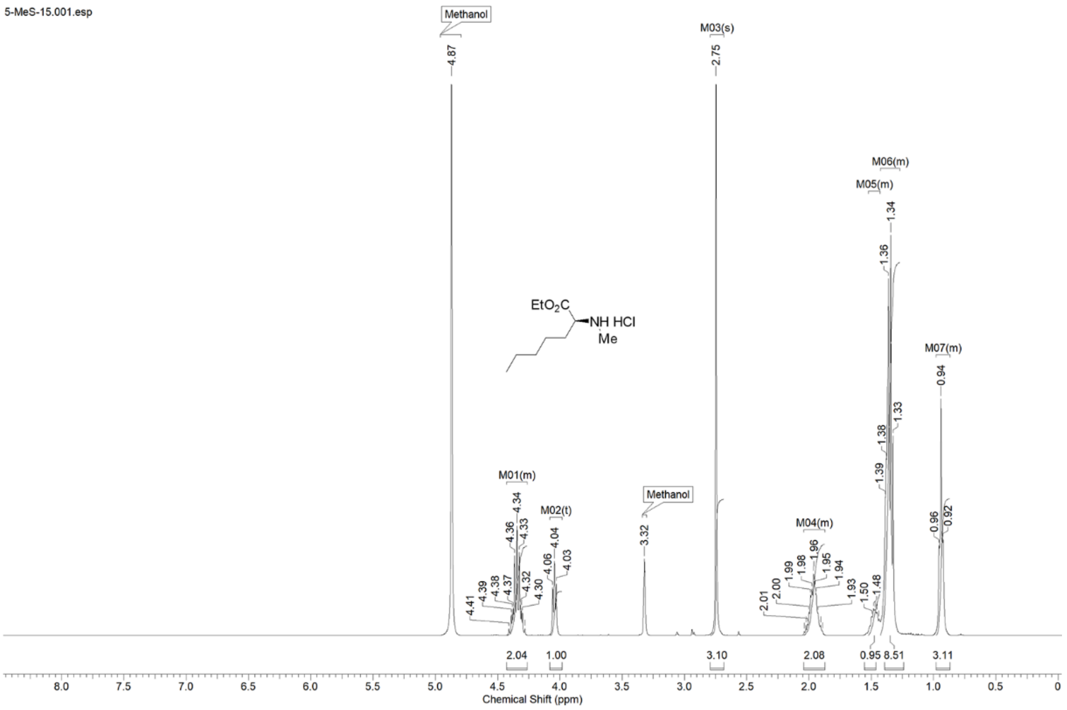

Figure S46. ^{13}C-NMR spectra of product ethyl (*S*)-2-(methylamino)heptanoate hydrochloride ((*S*)-**6b**) of IR358.

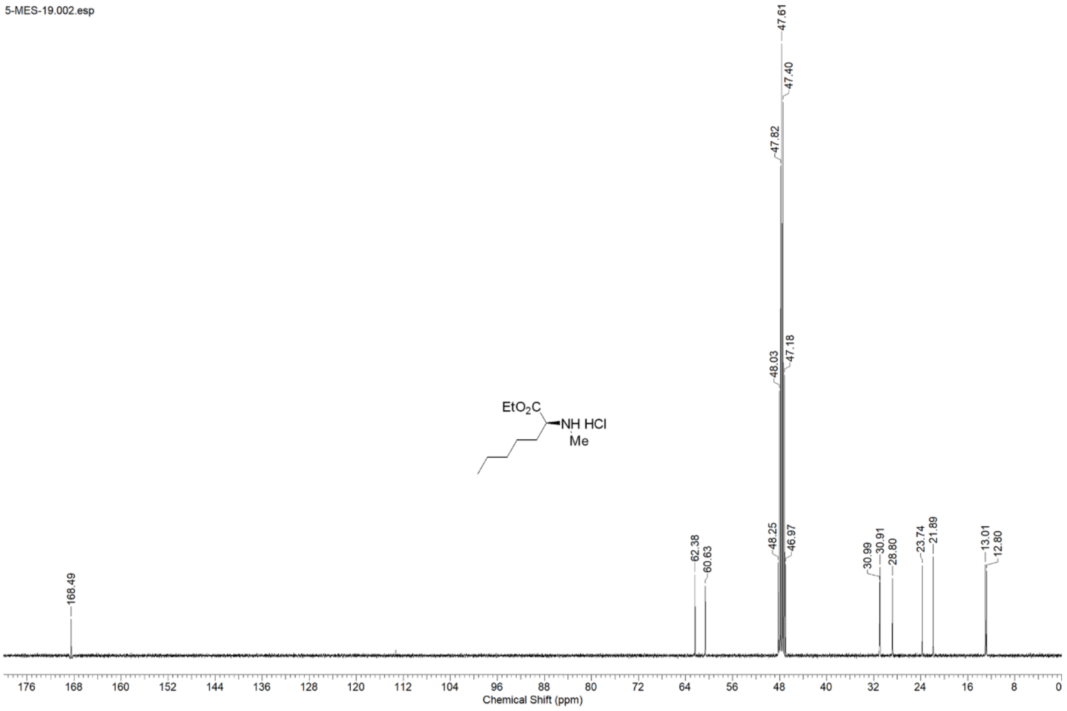

Figure S47. ^{1}H-NMR spectra of product ethyl (*R*)-4-(2-fluorophenyl)-2-(propargylamino)butanoate hydrochloride ((*R*)-**7a**) of IR271.

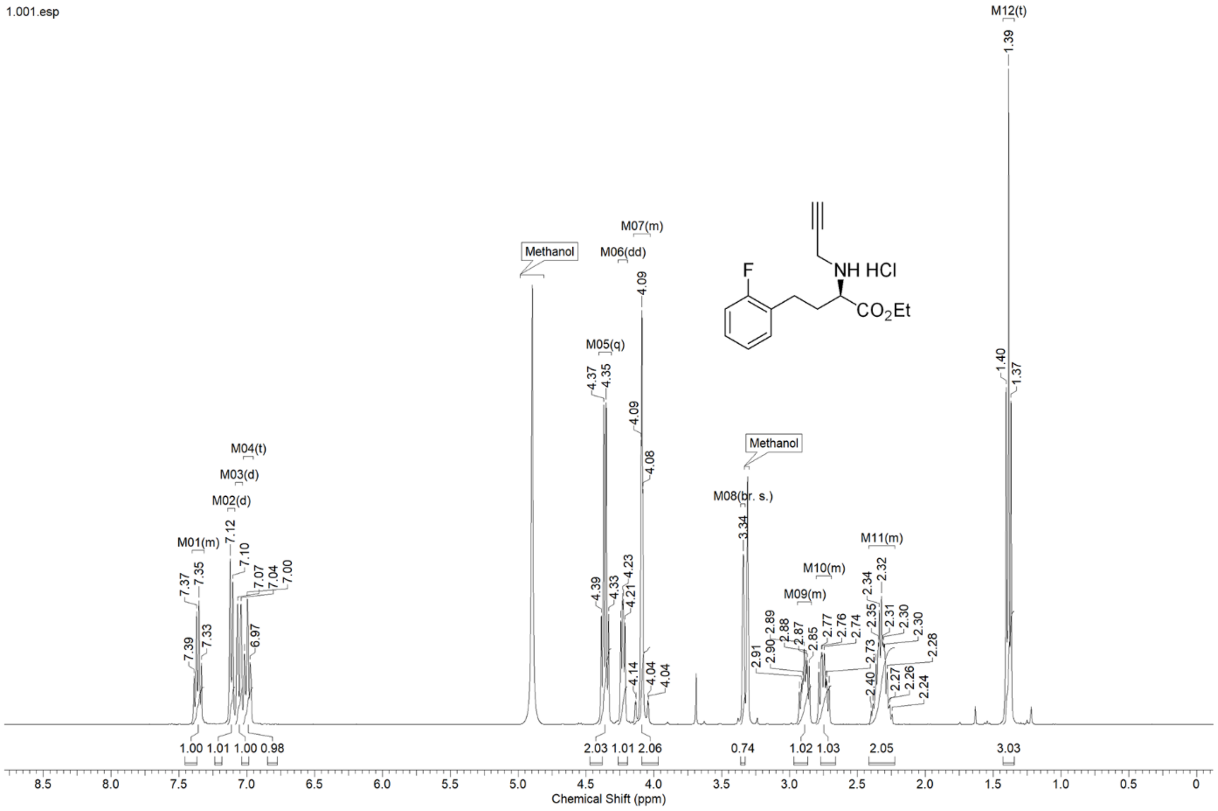

Figure S48. ^{13}C-NMR spectra of product ethyl (*R*)-4-(2-fluorophenyl)-2-(propargylamino)butanoate hydrochloride ((*R*)-**7a**) of IR271

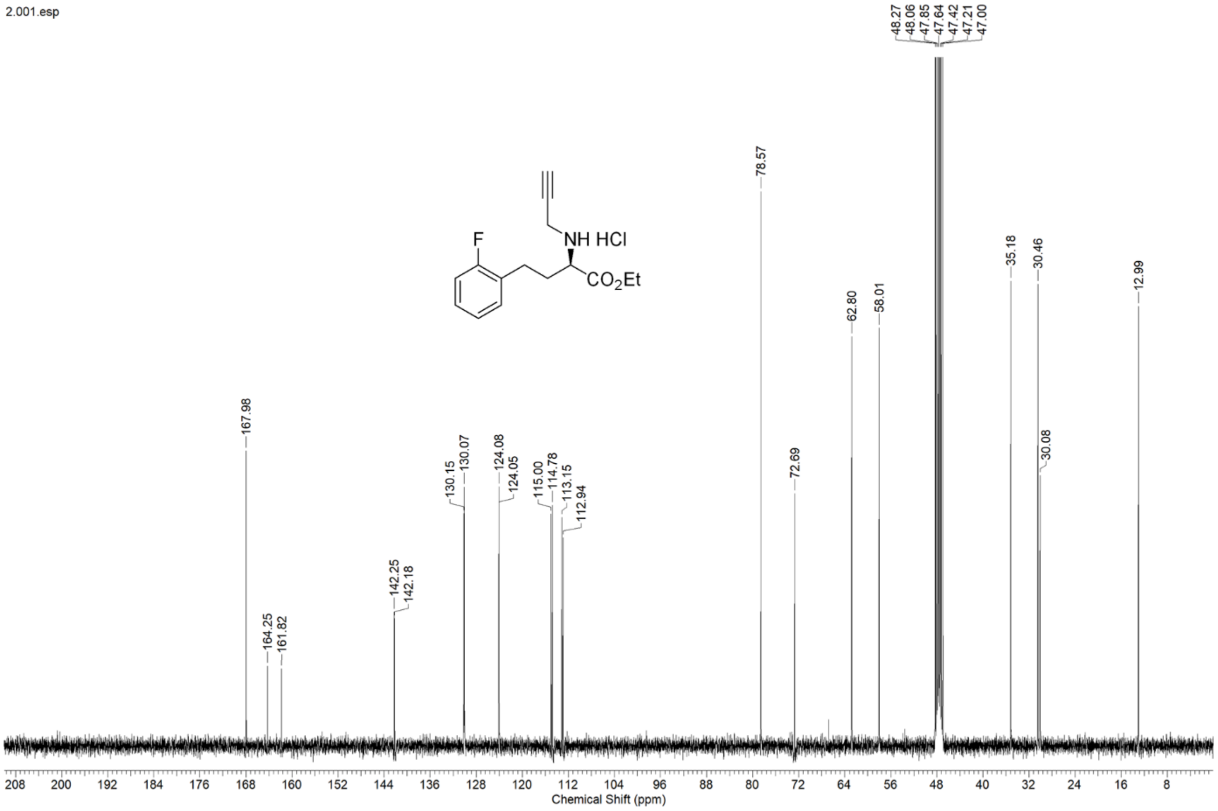

Figure S49. ^{1}H-NMR spectra of product ethyl (*S*)-4-(2-fluorophenyl)-2-(propargylamino)butanoate hydrochloride ((*S*)-**7a**) of IR338.

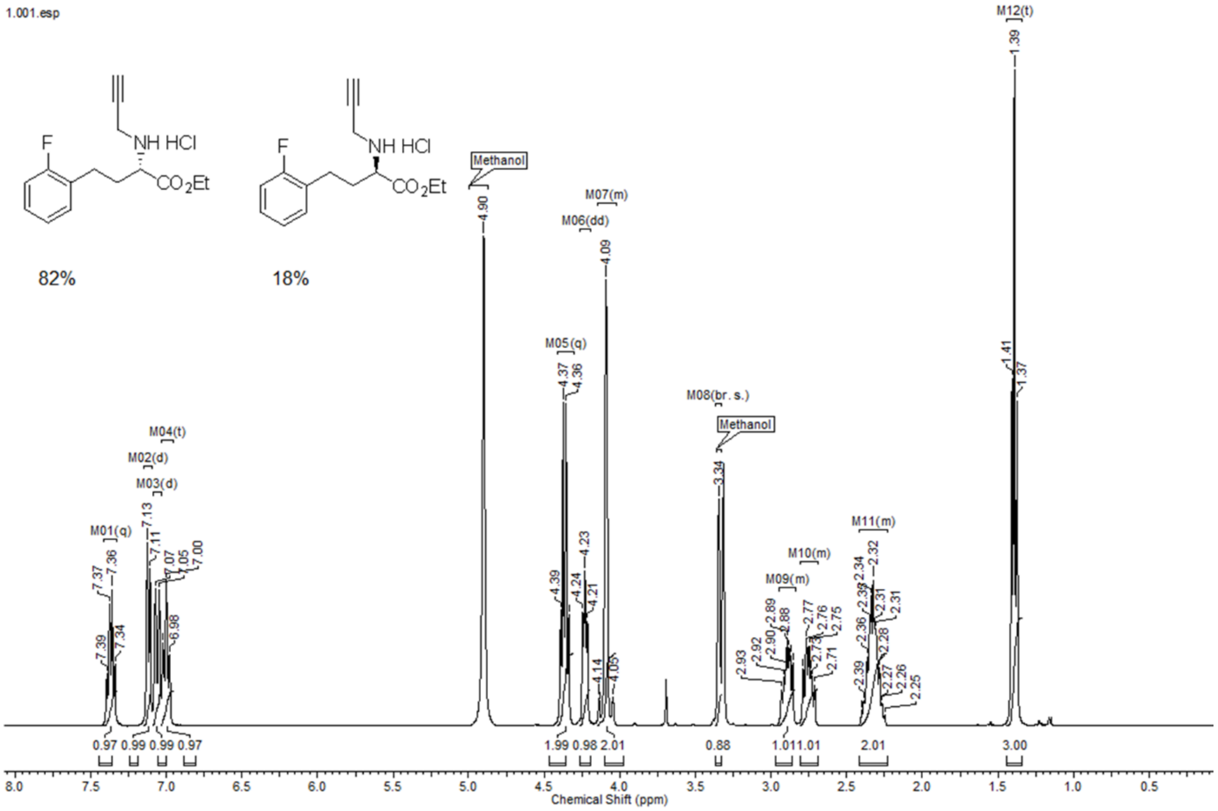

Figure S50. ^{13}C-NMR spectra of product ethyl (*S*)-4-(2-fluorophenyl)-2-(propargylamino)butanoate hydrochloride ((*S*)-**7a**) of IR338

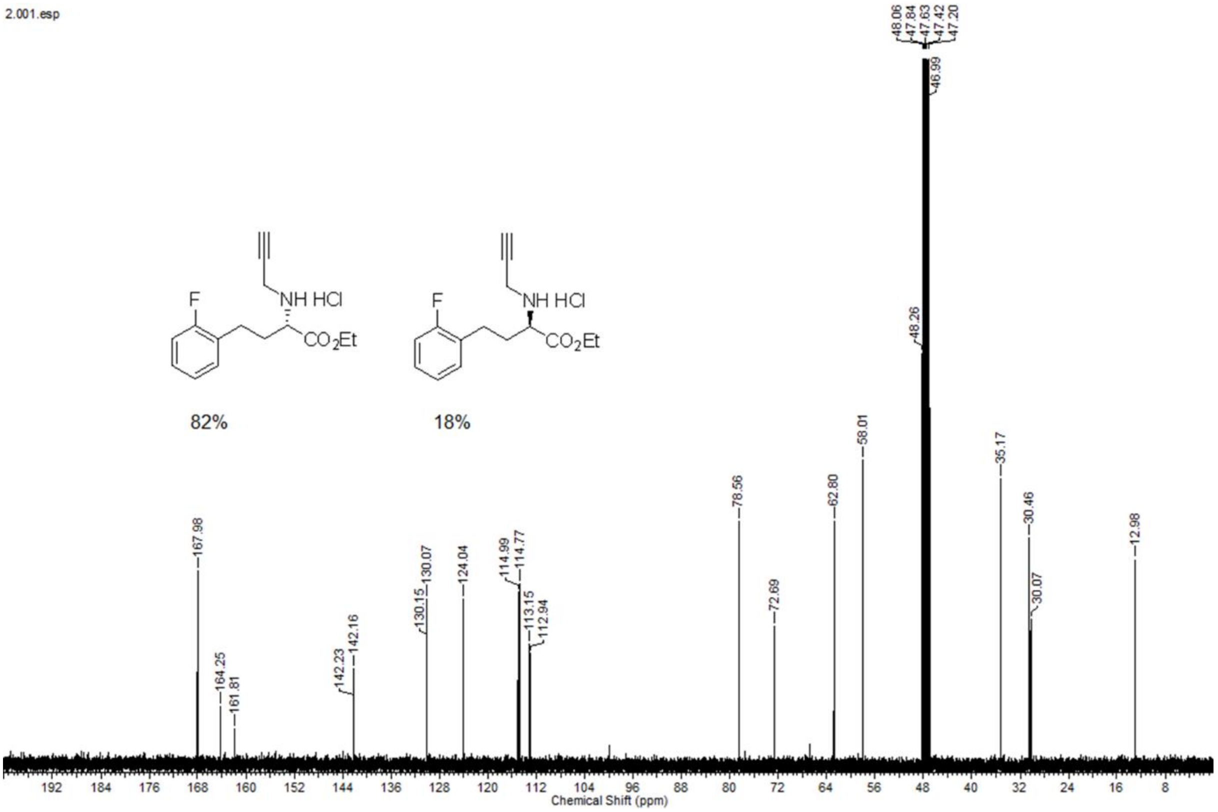

Figure S51. ^{1}H-NMR spectra of product ethyl (*R*)-4-(3-fluorophenyl)-2-(propargylamino)butanoate hydrochloride ((*R*)-**8a**) of IR271.

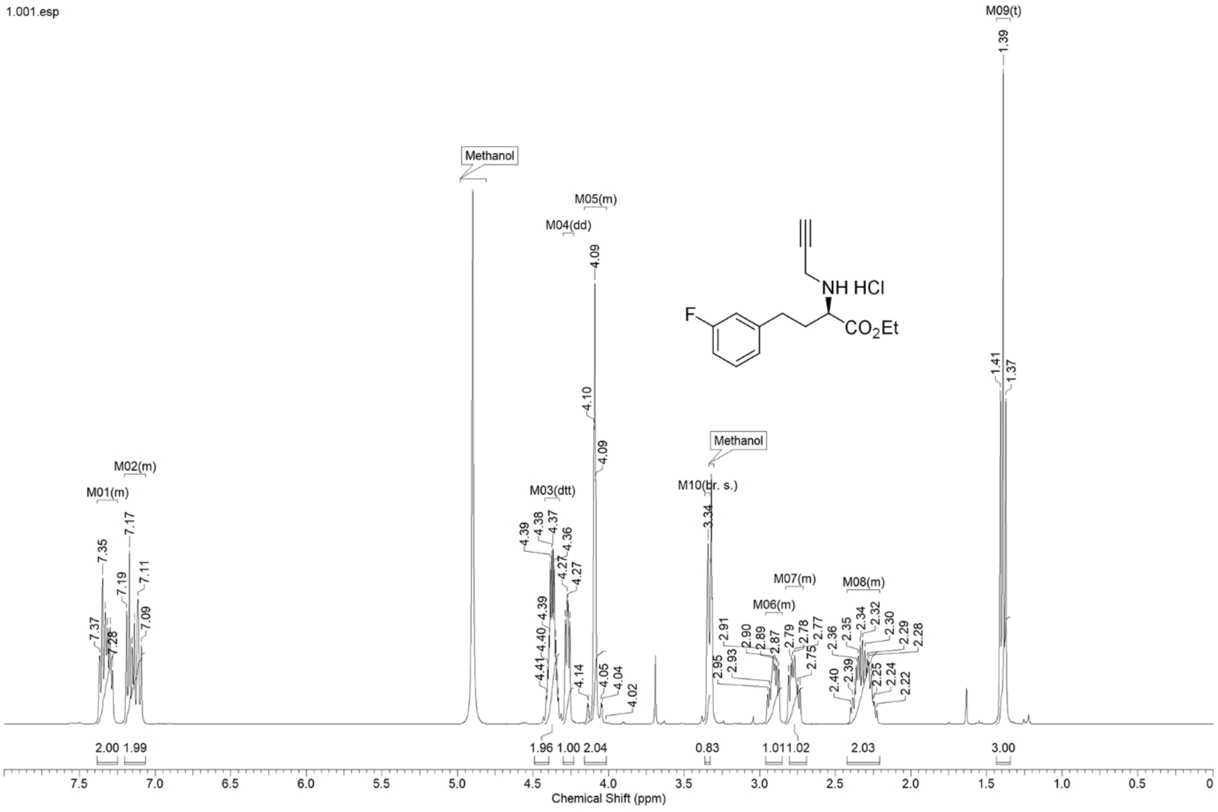

Figure S52. ^{13}C-NMR spectra of product ethyl (*R*)-4-(3-fluorophenyl)-2-(propargylamino)butanoate hydrochloride ((*R*)-**8a**) of IR271

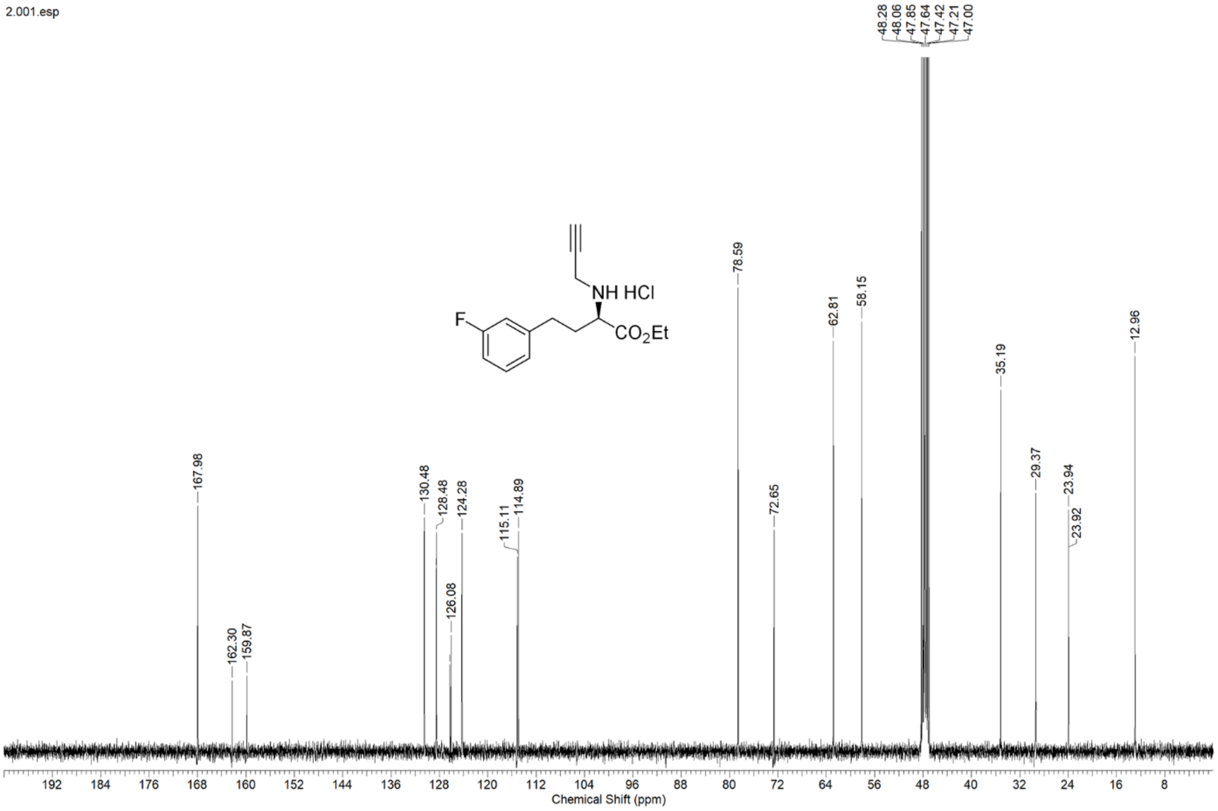

Figure S53. ^{1}H-NMR spectra of product ethyl (*S*)-4-(3-fluorophenyl)-2-(propargylamino)butanoate hydrochloride ((*S*)-**8a**) of IR338.

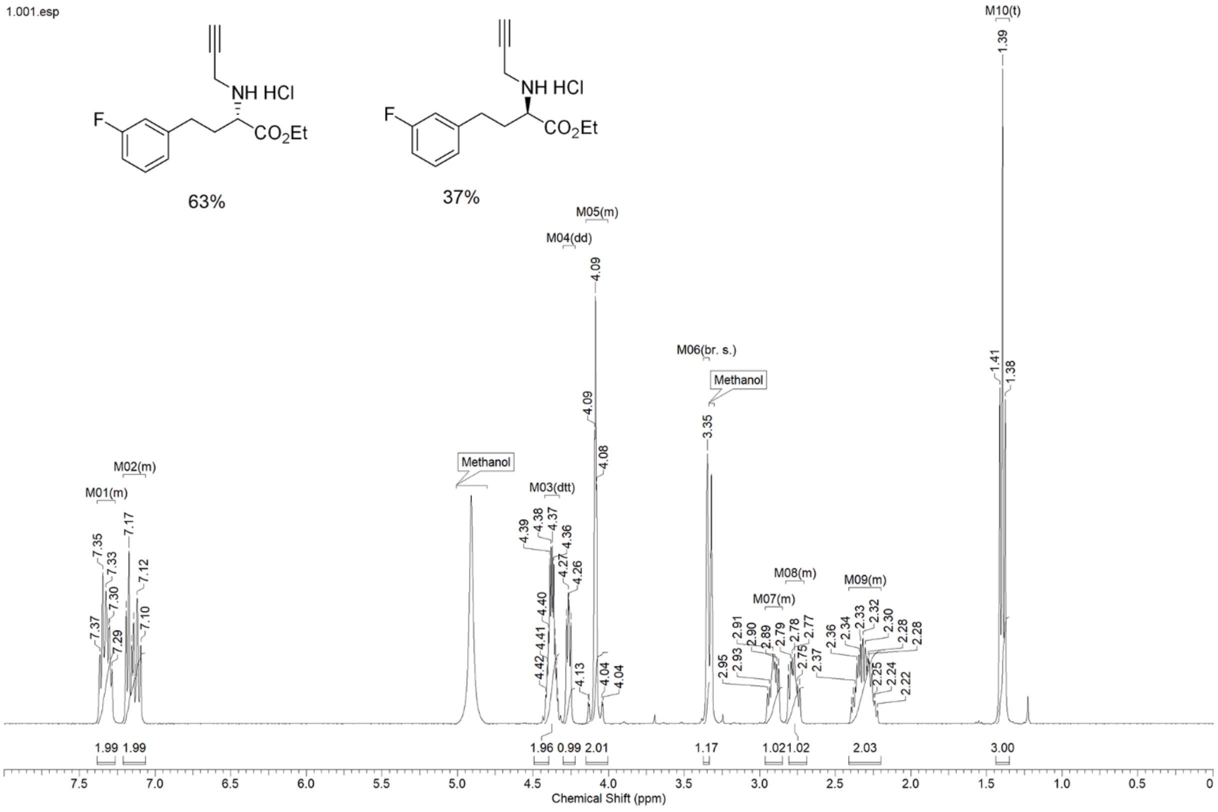

Figure S54. ^{13}C-NMR spectra of product ethyl (*S*)-4-(3-fluorophenyl)-2-(propargylamino)butanoate hydrochloride ((*S*)-**8a**) of IR338

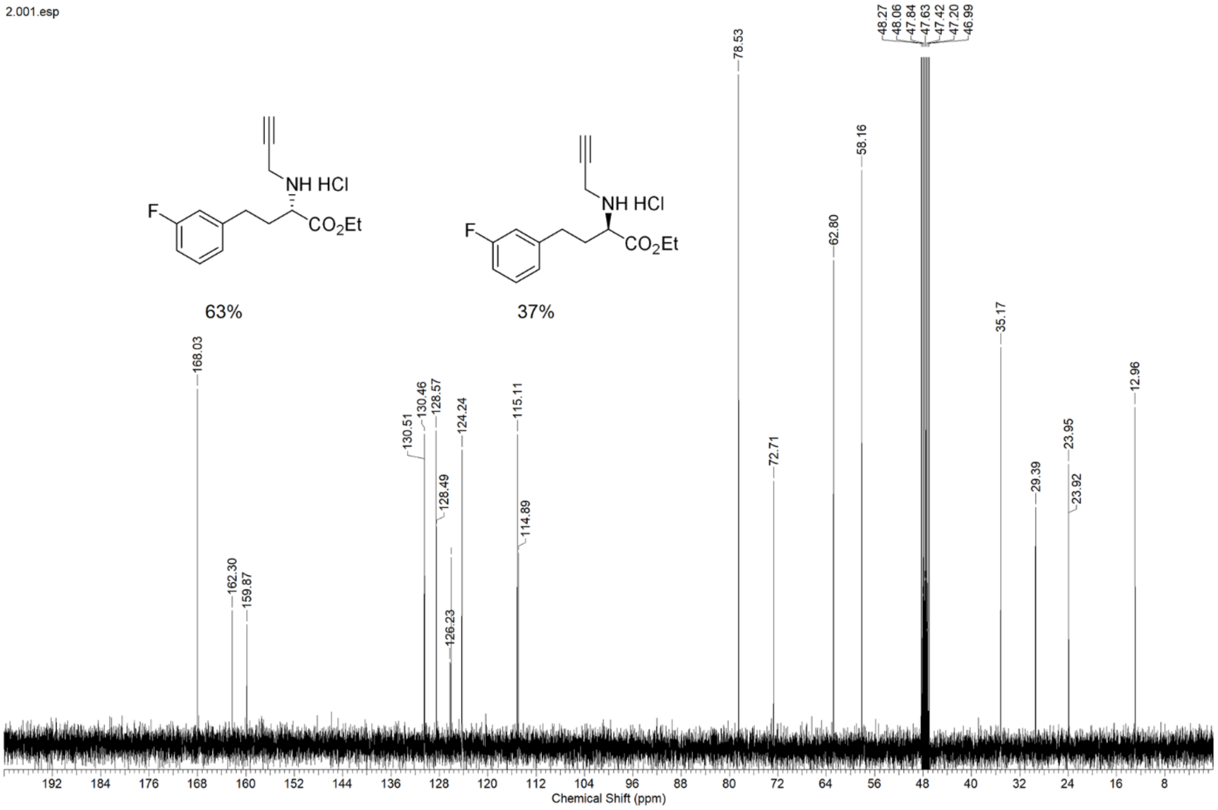

Figure S55. ^{1}H-NMR spectra of product ethyl (*R*)-4-(4-fluorophenyl)-2-(propargylamino)butanoate hydrochloride ((*R*)-**8a**) of IR271.

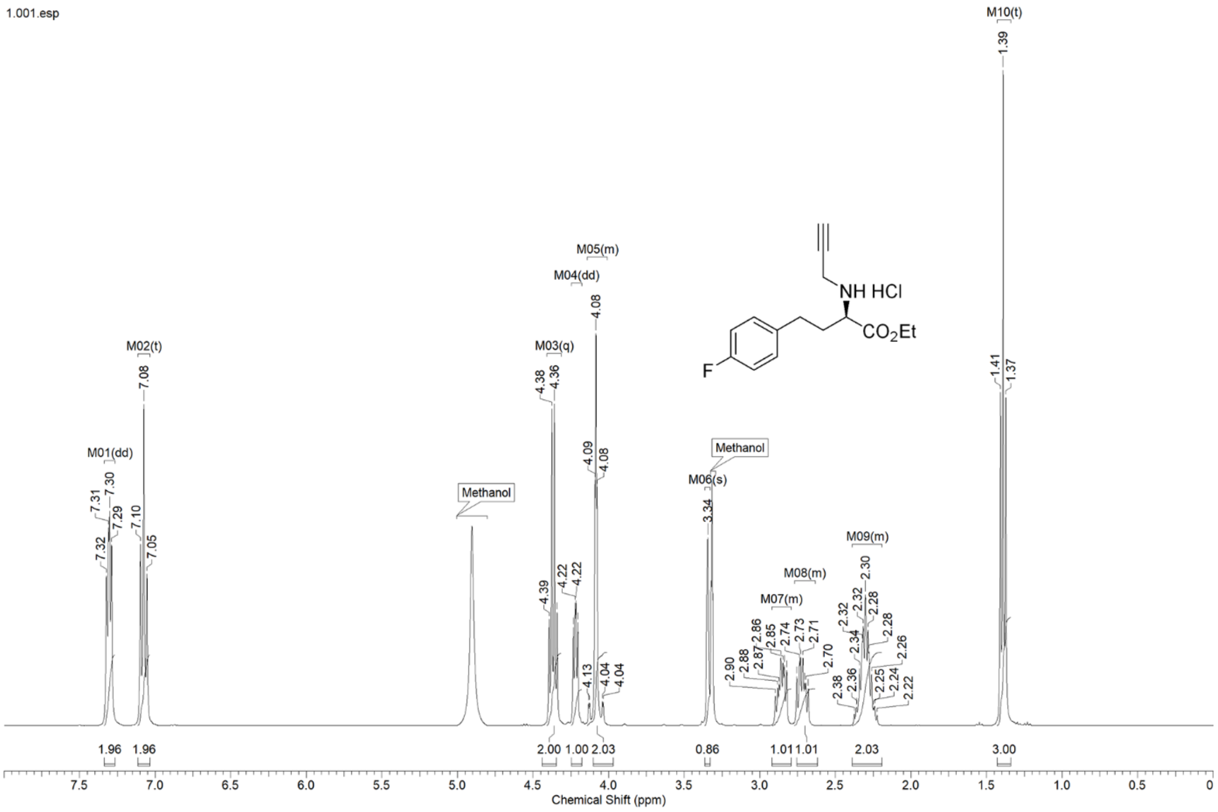

Figure S56. ^{13}C-NMR spectra of product ethyl (*R*)-4-(4-fluorophenyl)-2-(propargylamino)butanoate hydrochloride ((*R*)-**8a**) of IR271

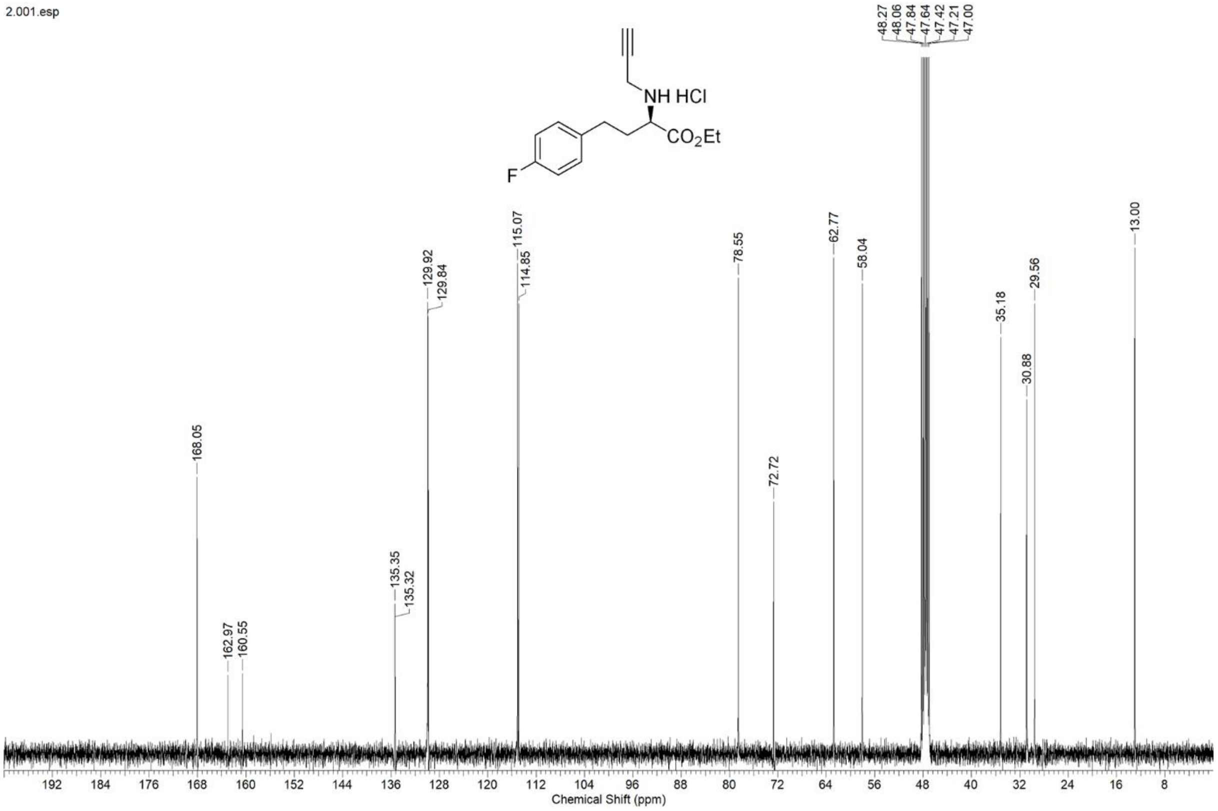

Figure S57. ^{1}H-NMR spectra of product ethyl (*S*)-4-(4-fluorophenyl)-2-(propargylamino)butanoate hydrochloride ((*S*)-**8a**) of IR338.

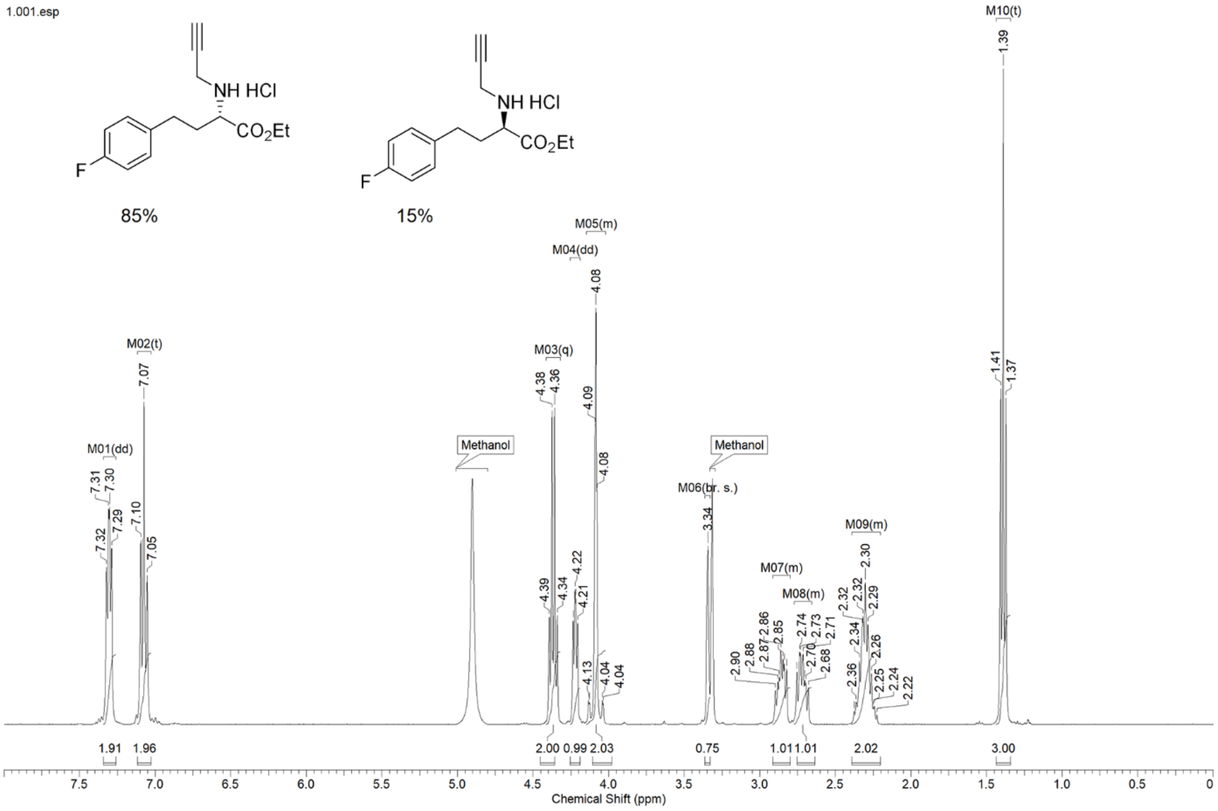

Figure S58. ^{13}C-NMR spectra of product ethyl (*S*)-4-(4-fluorophenyl)-2-(propargylamino)butanoate hydrochloride ((*S*)-**8a**) of IR338

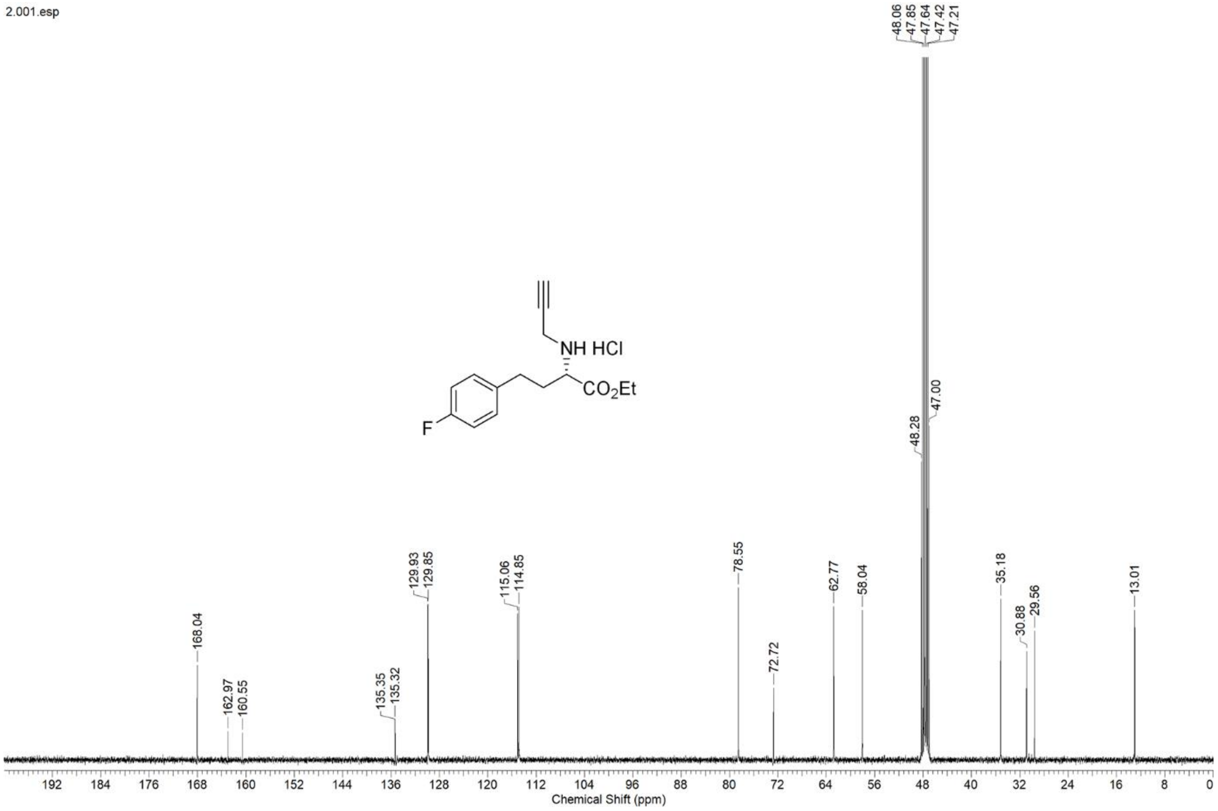

3.4. ^{1}H-NMR and ^{13}C-NMR spectra of standard products

Figure S59. ^{1}H-NMR spectra of standard product ethyl (*R*)-4-phenyl-2-(propargylamino) butyrate hydrochloride ((*R*)-**1a**).

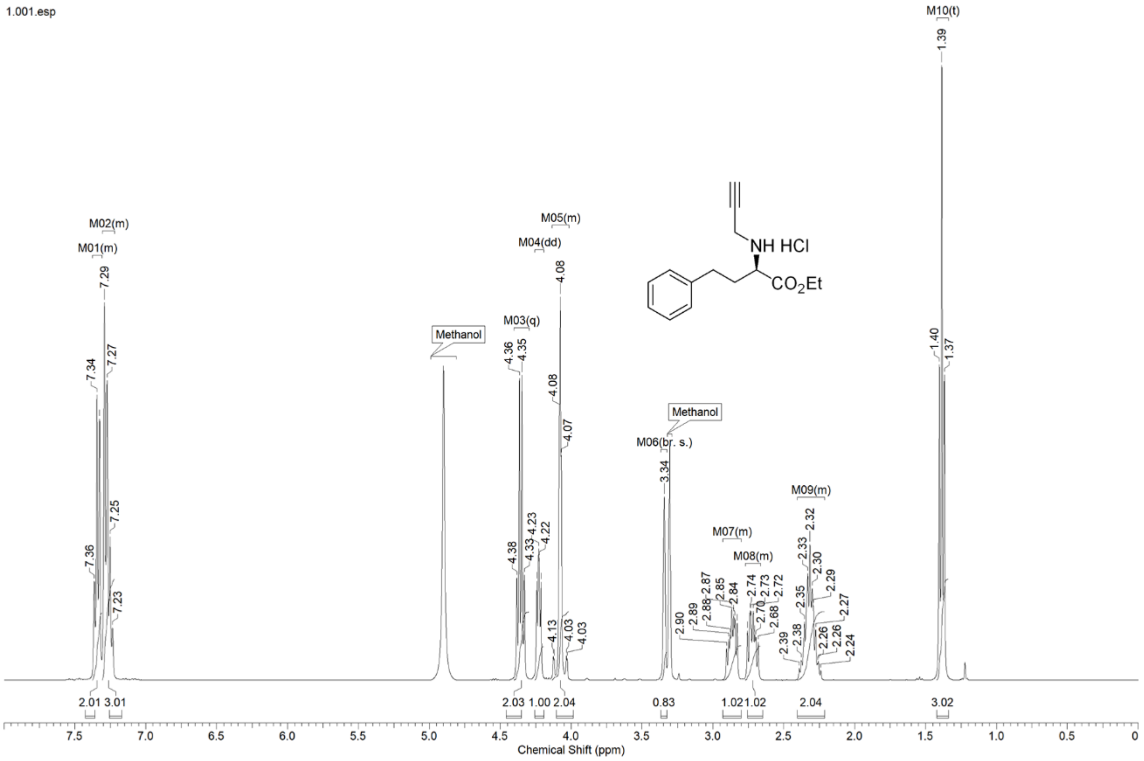

Figure S60. ^{13}C-NMR spectra of standard product ethyl (*R*)-4-phenyl-2-(propargylamino) butyrate hydrochloride ((*R*)-**1a**).

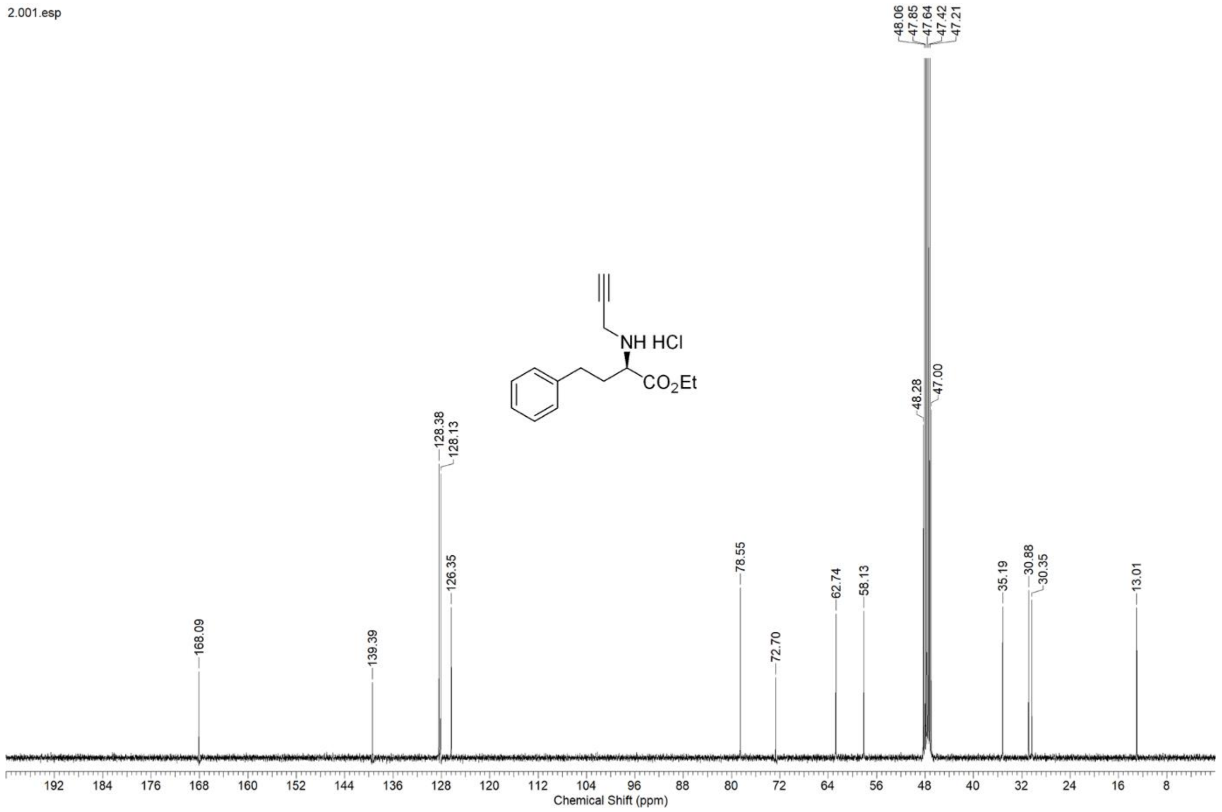

Figure S61. ^{1}H-NMR spectra of standard product ethyl (*R*)-4-phenyl-2-((4-methylbenzyl)amino) butyrate hydrochloride ((*R*)-**1g**).

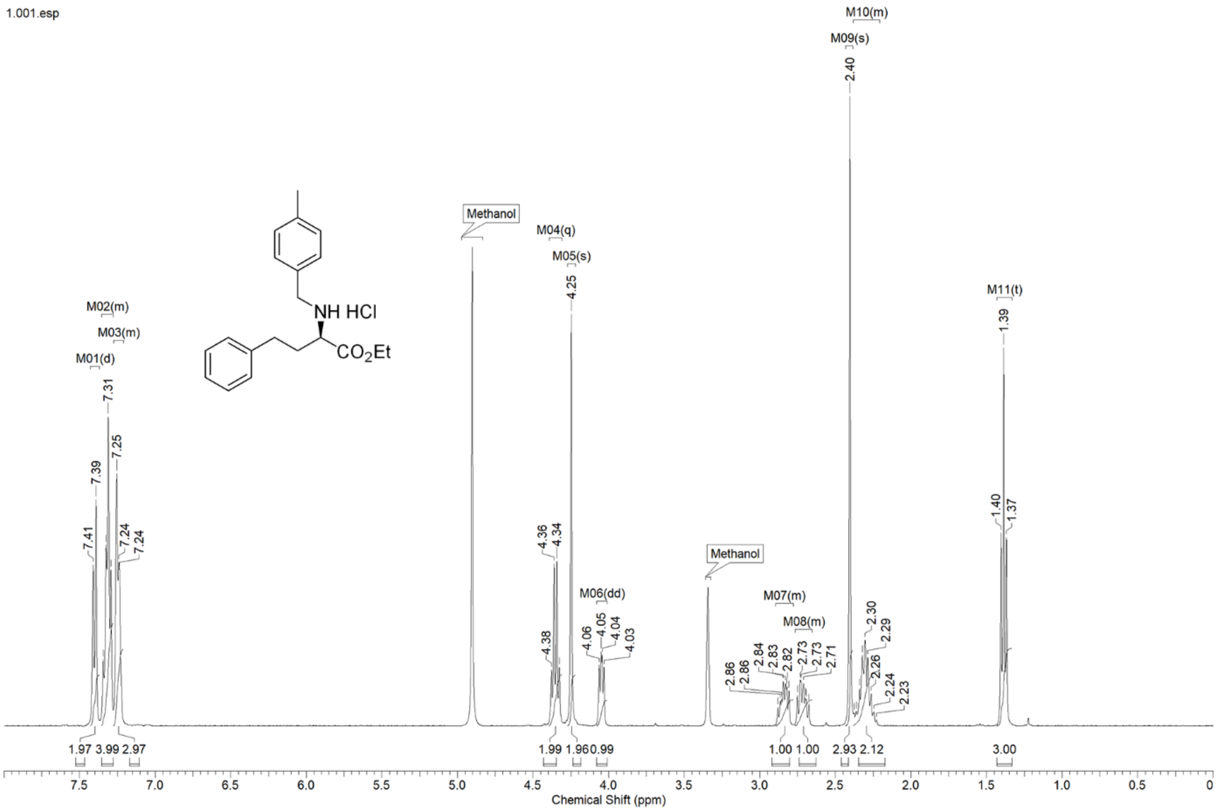

Figure S62. ^{13}C-NMR spectra of standard product ethyl (*R*)-4-phenyl-2-((4-methylbenzyl)amino) butyrate hydrochloride ((*R*)-**1g**).

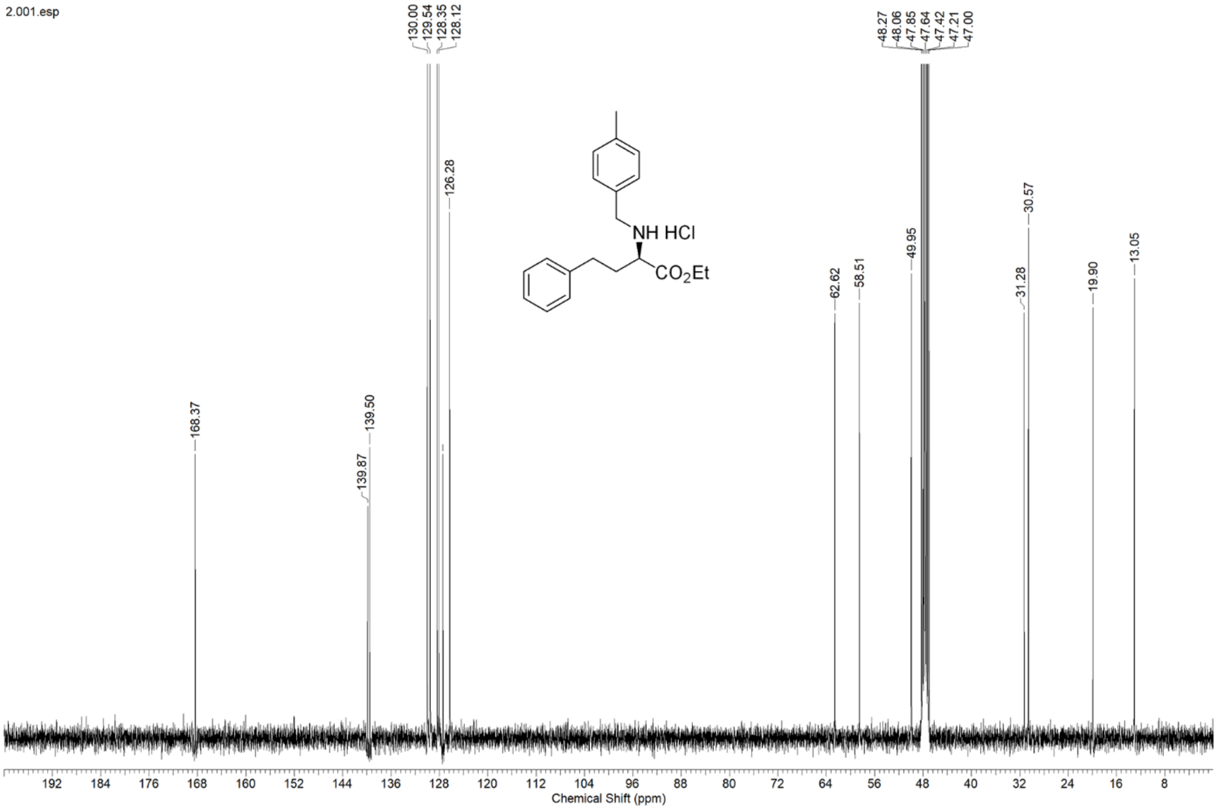

Figure S63. ^{1}H-NMR spectra of standard product ethyl (*R*)-3-phenyl-2-(propargylamino)propionate ((*R*)-**2a**).

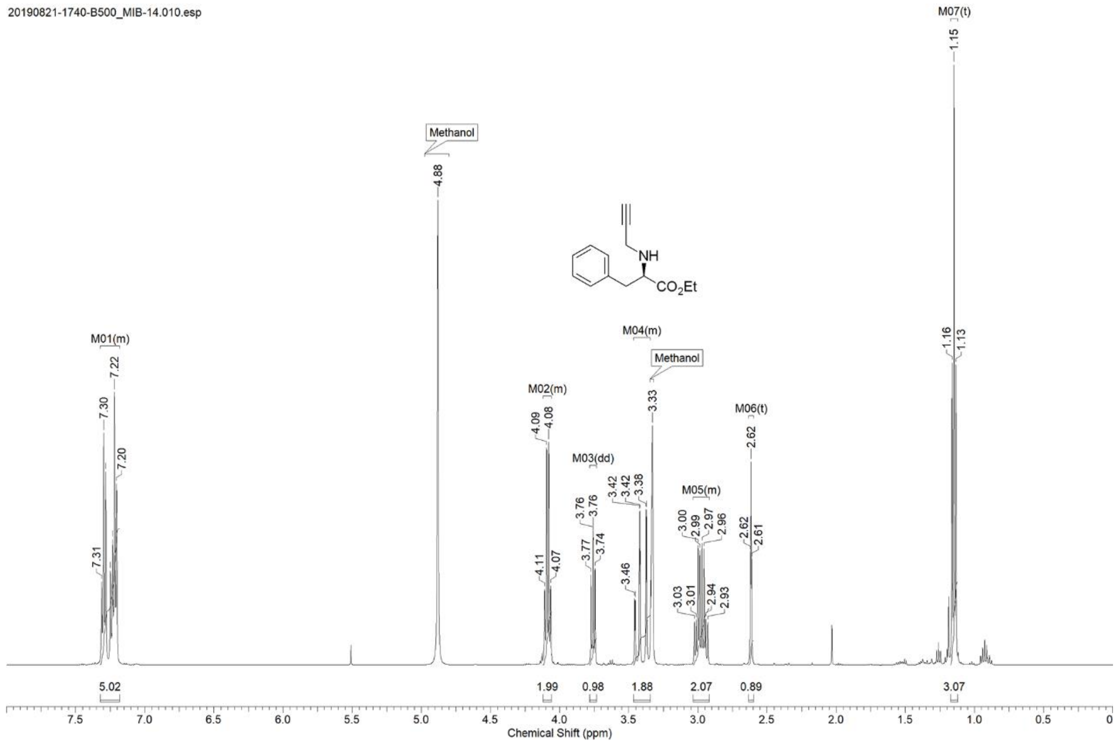

Figure S64. ^{13}C-NMR spectra of standard product ethyl (*R*)-3-phenyl-2-(propargylamino)propionate ((*R*)-**2a**).

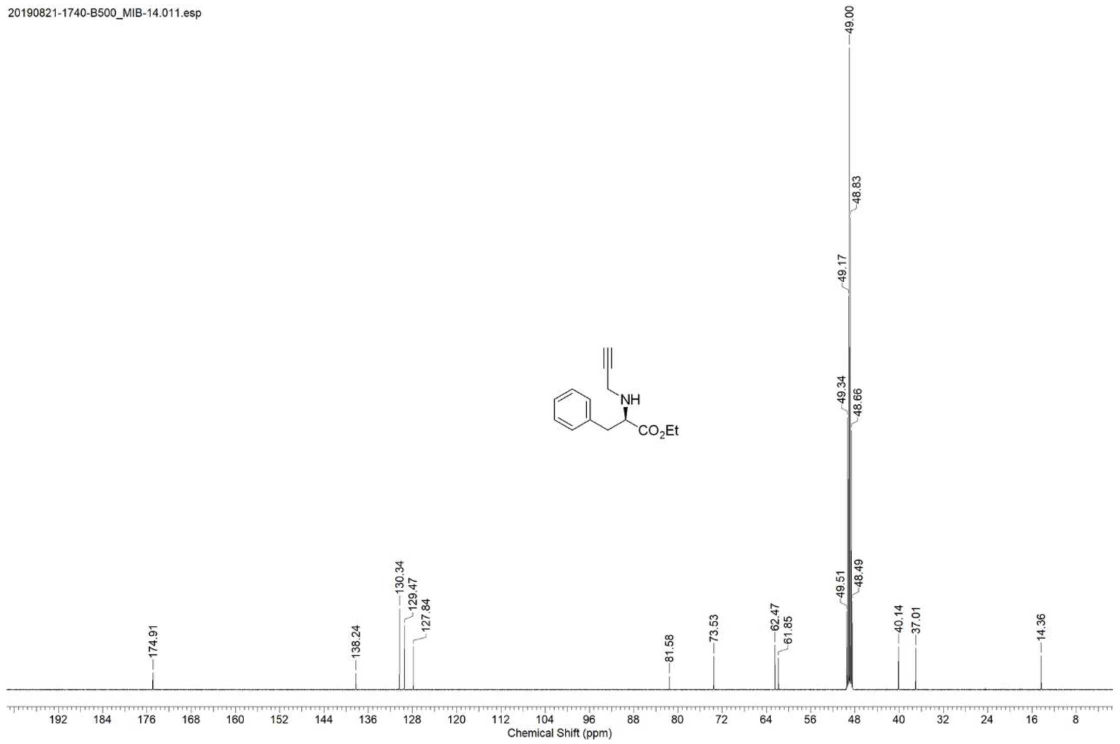

Figure S65. ^{1}H-NMR spectra of standard product ethyl (*S*)-2-(propargylamino) propionate ((*S*)-**3a**).

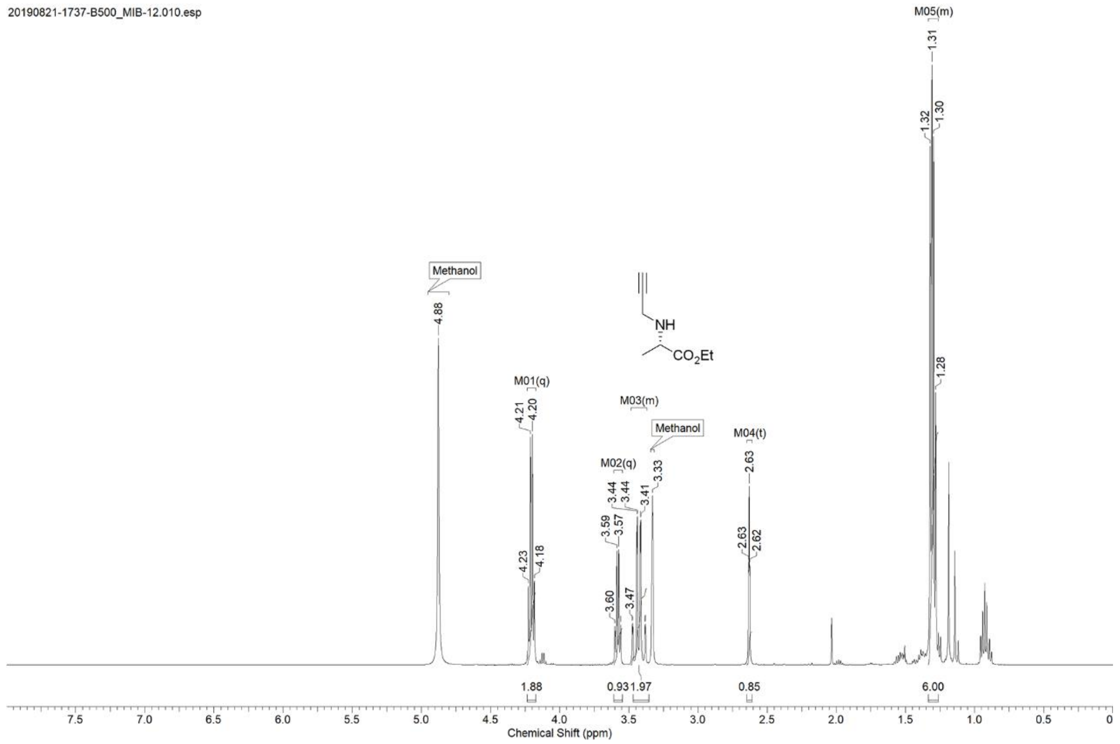

Figure S66. ^{13}C-NMR spectra of standard product ethyl (*S*)-2-(propargylamino) propionate ((*S*)-**3a**).

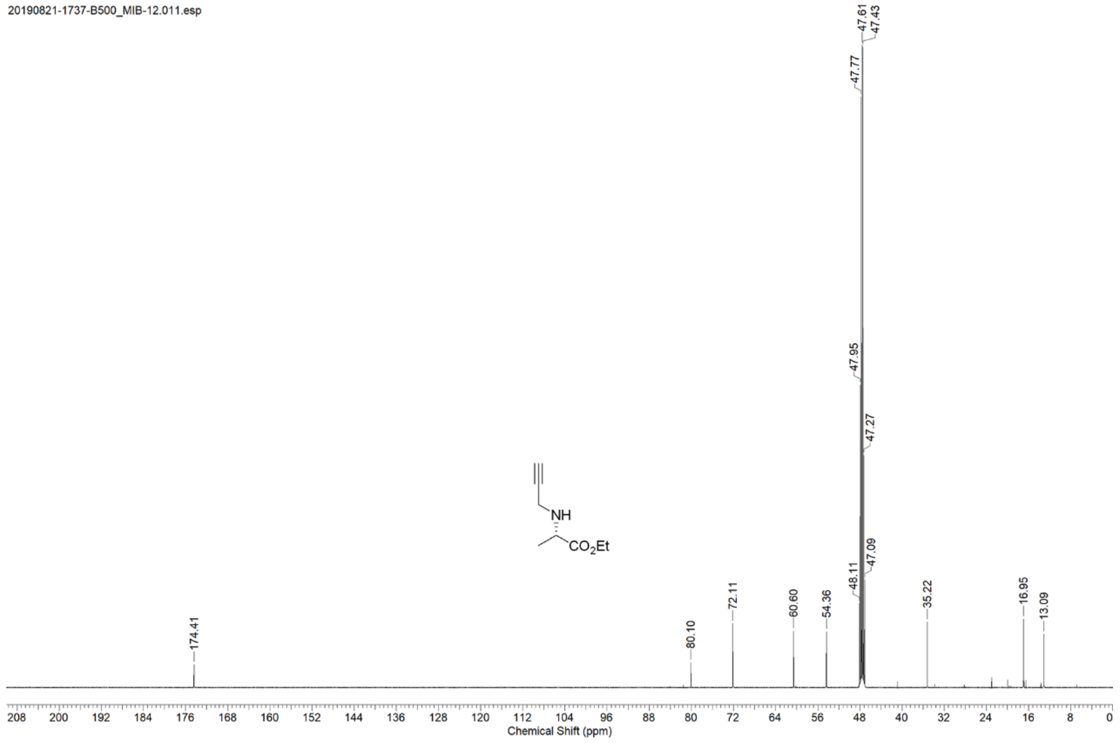

Figure S67. ^{1}H-NMR spectra of standard product ethyl (*R*)-2-(propargylamino) valerate ((*R*)-**4a**).

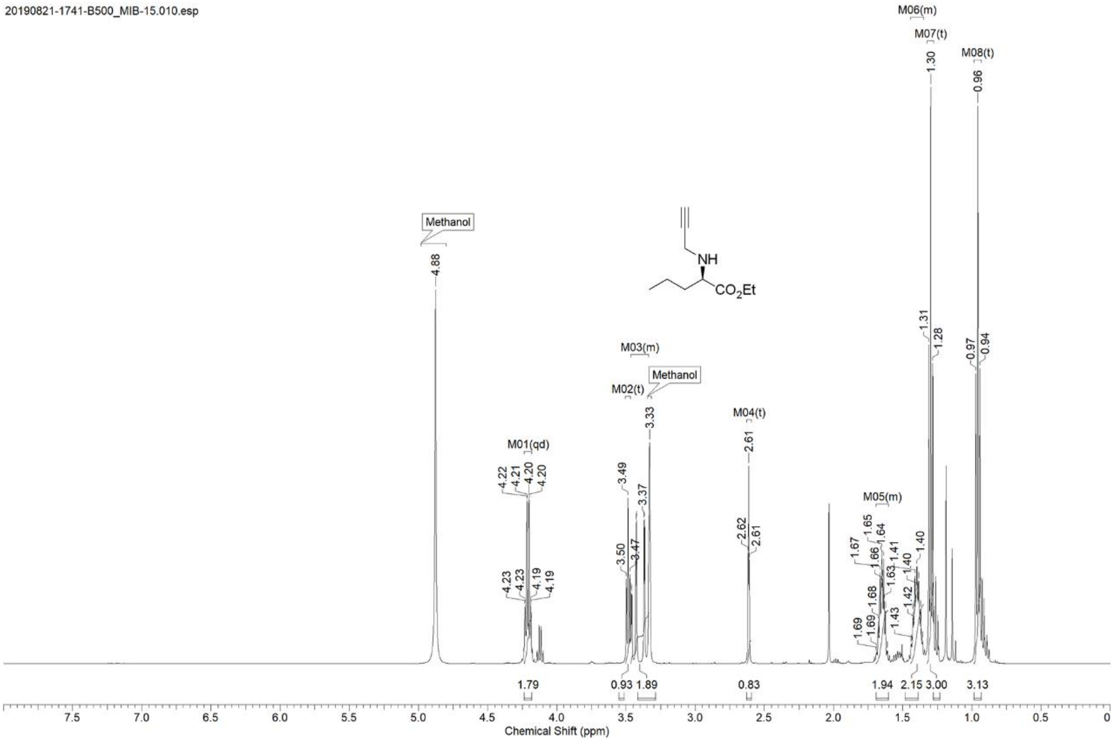

Figure S68. ^{13}C-NMR spectra of standard product ethyl (*R*)-2-(propargylamino) valerate ((*R*)-**4a**).

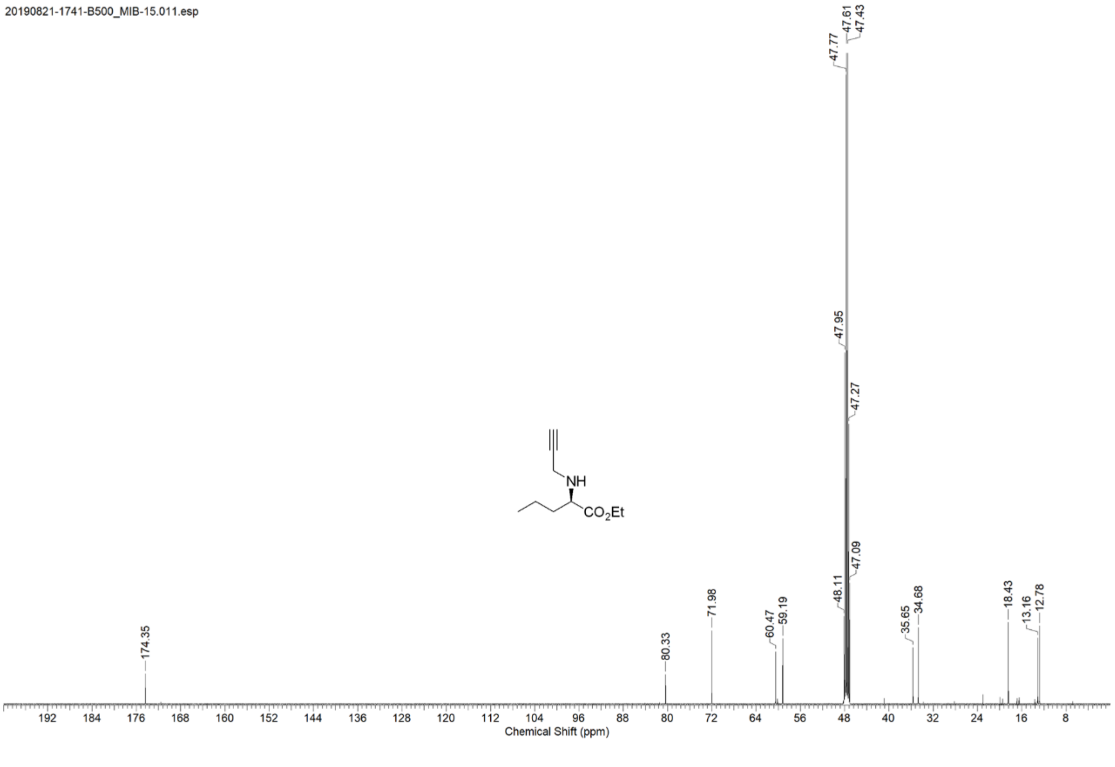

Figure S69. ^{1}H-NMR spectra of standard product ethyl (*S*)-4-methyl-2-(propargylamino) valerate ((*S*)-**5a**).

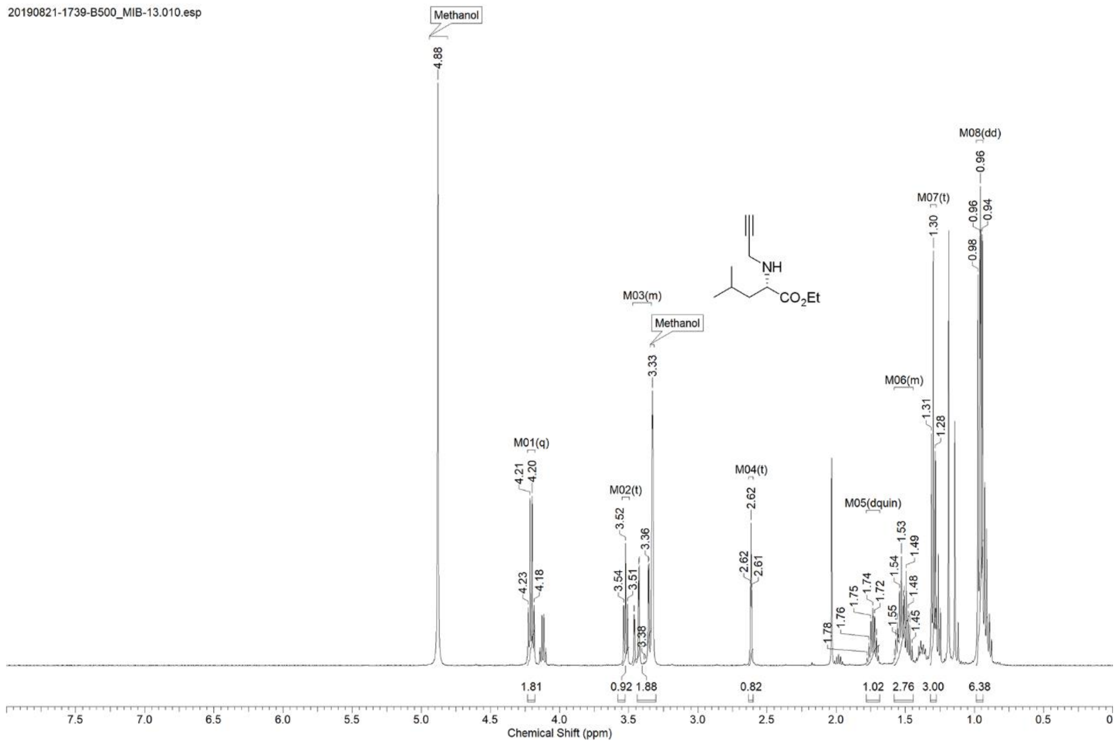

Figure S70. ^{13}C-NMR spectra of standard product ethyl (*S*)-4-methyl-2-(propargylamino) valerate ((*S*)-**5a**).

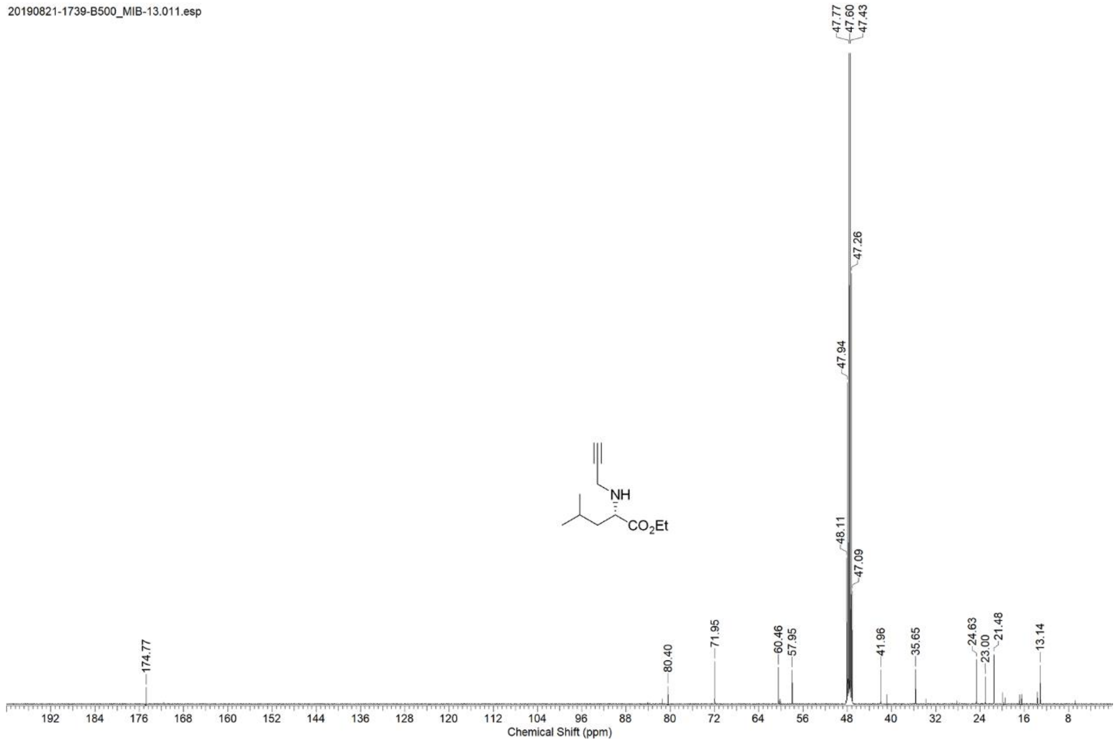

Figure S71. ^{1}H-NMR spectra of standard product ethyl (*S*)-2-phenyl-2-(propargylamino) acetate hydrochloride ((*S*)-**10a**).

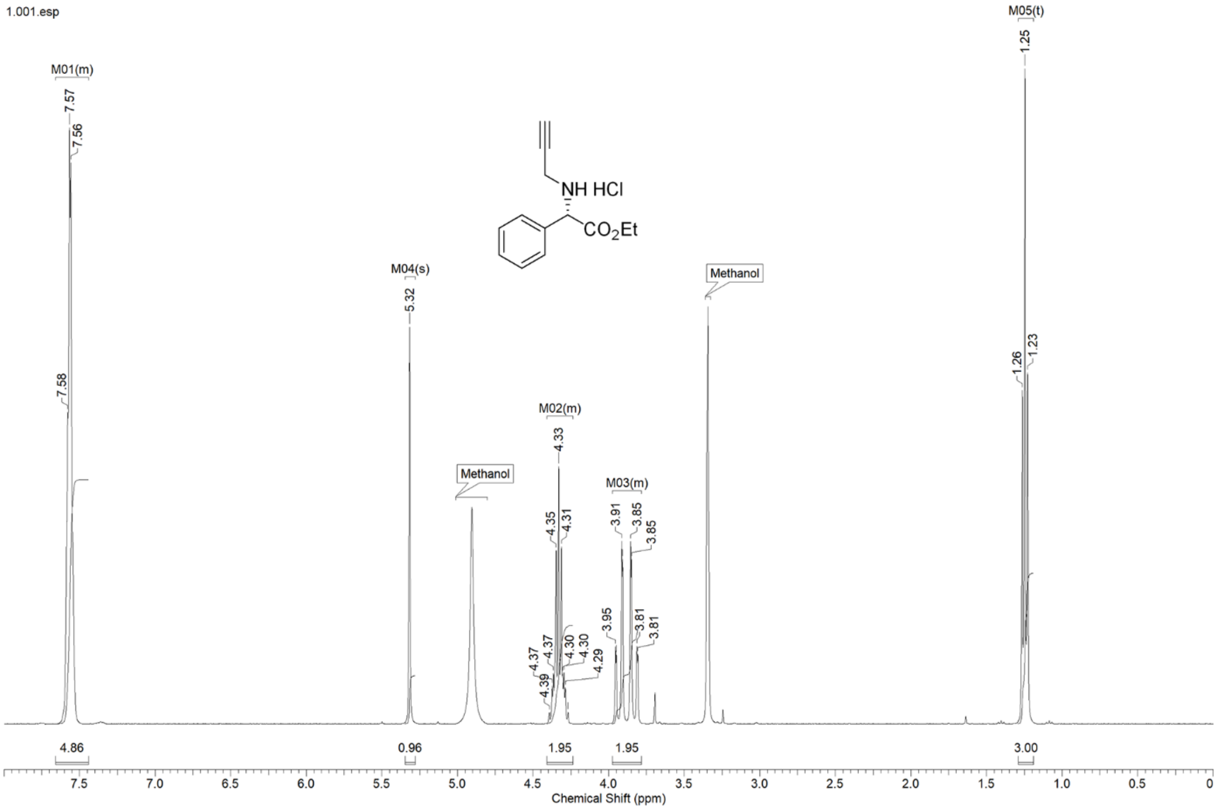

Figure S72. ^{13}C-NMR spectra of standard product ethyl (*S*)-2-phenyl-2-(propargylamino) acetate hydrochloride ((*S*)-**10a**).

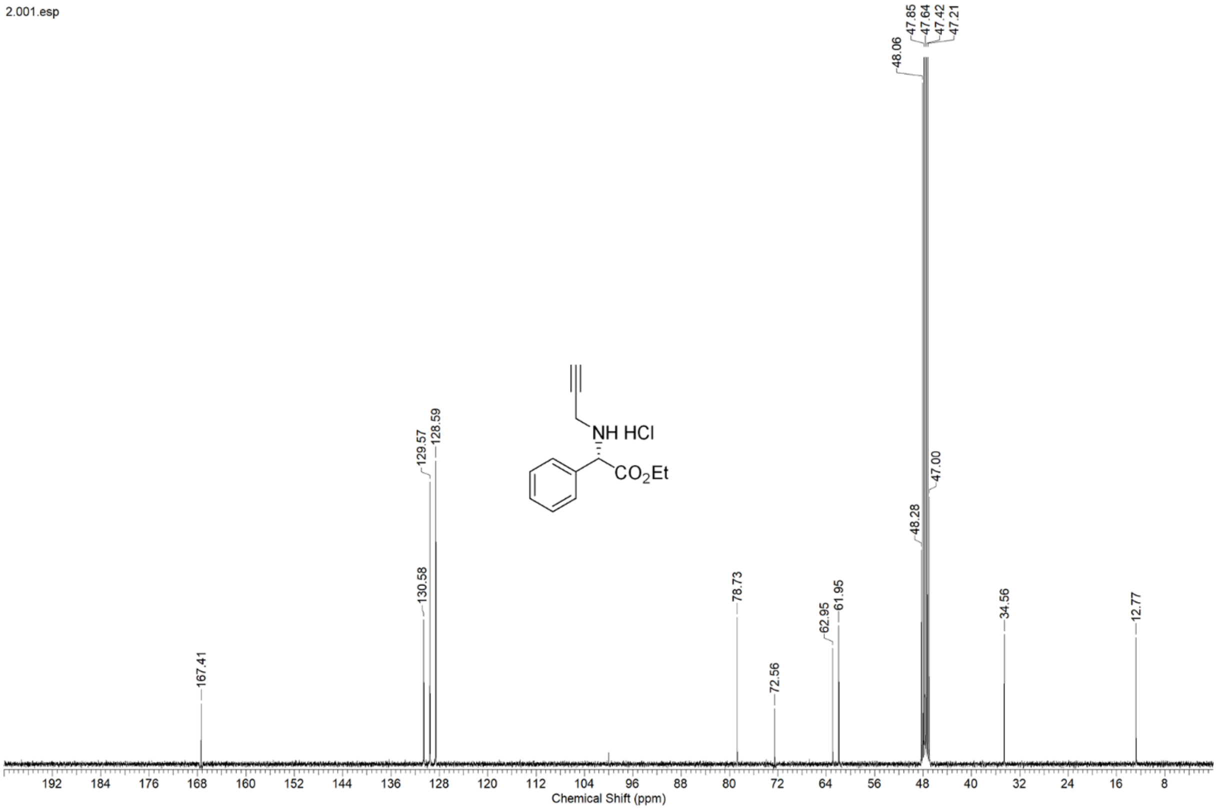

Figure S73. ^{1}H-NMR spectra of standard product ethyl *N*-propargyl-D-serinate hydrochloride ((*R*)-**11a**).

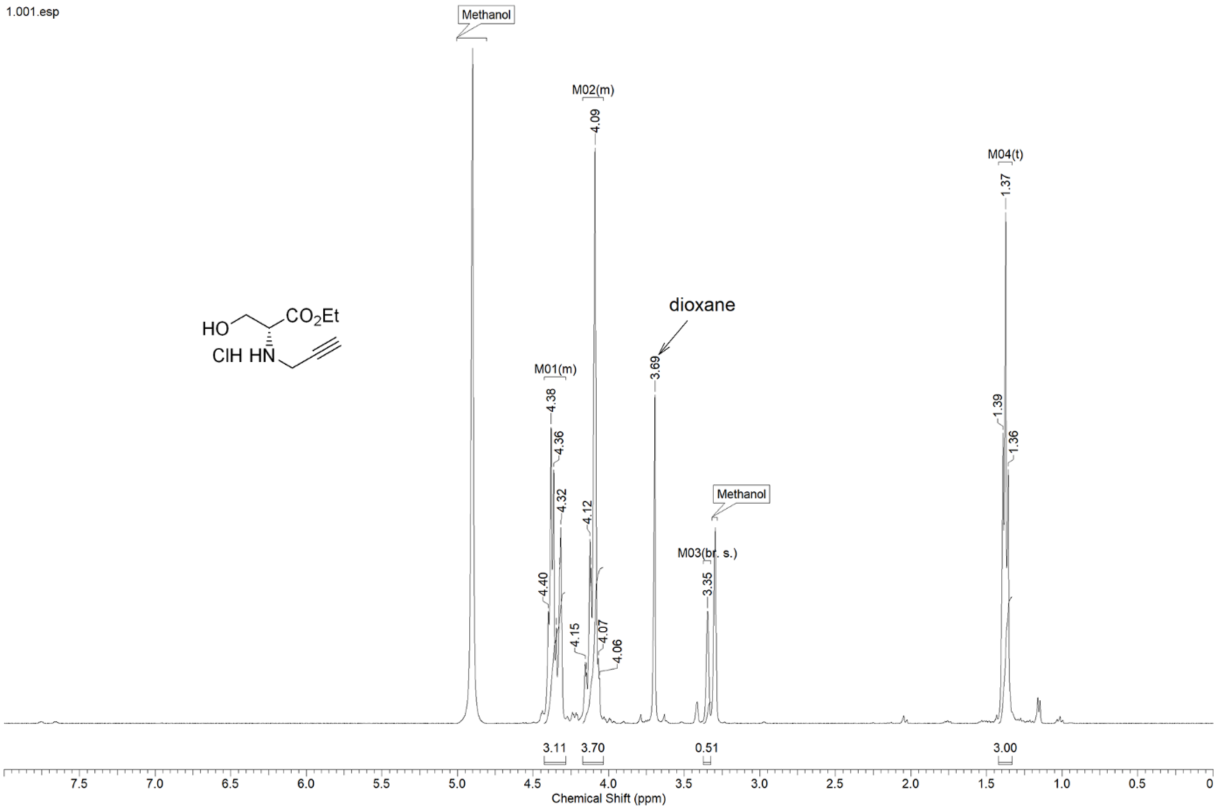

Figure S74. ^{13}C-NMR spectra of standard product ethyl *N*-propargyl-D-serinate hydrochloride ((*R*)-**11a**).

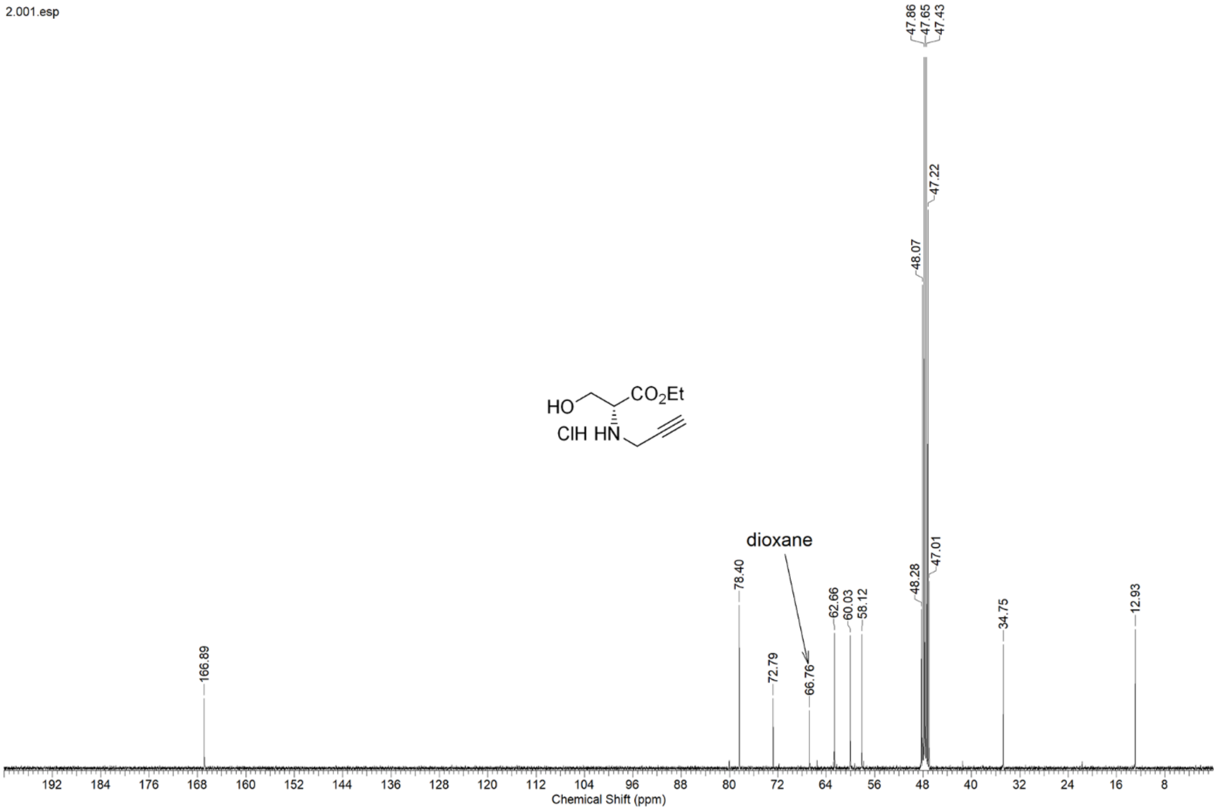

3.5. GC and HPLC spectra

Figure S74. HPLC spectra of racemic ethyl 4-phenyl-2-(propargylamino)butyrate (**1a**), products of IR271 ((*R*)-**1a**) and IR338 ((*S*)-**1a**), and standard of (*R*)-**1a**. HPLC condition: CHIRALPAK® AD-H column, n-hexane /2-propanol (0.1% diethylamine) = 97:3, flow rate = 1.0 mL/min, 254 nm UV detector, tR = 10.24 min (*R*) and tR = 11.33 min (*S*), column temperature = 25°C.

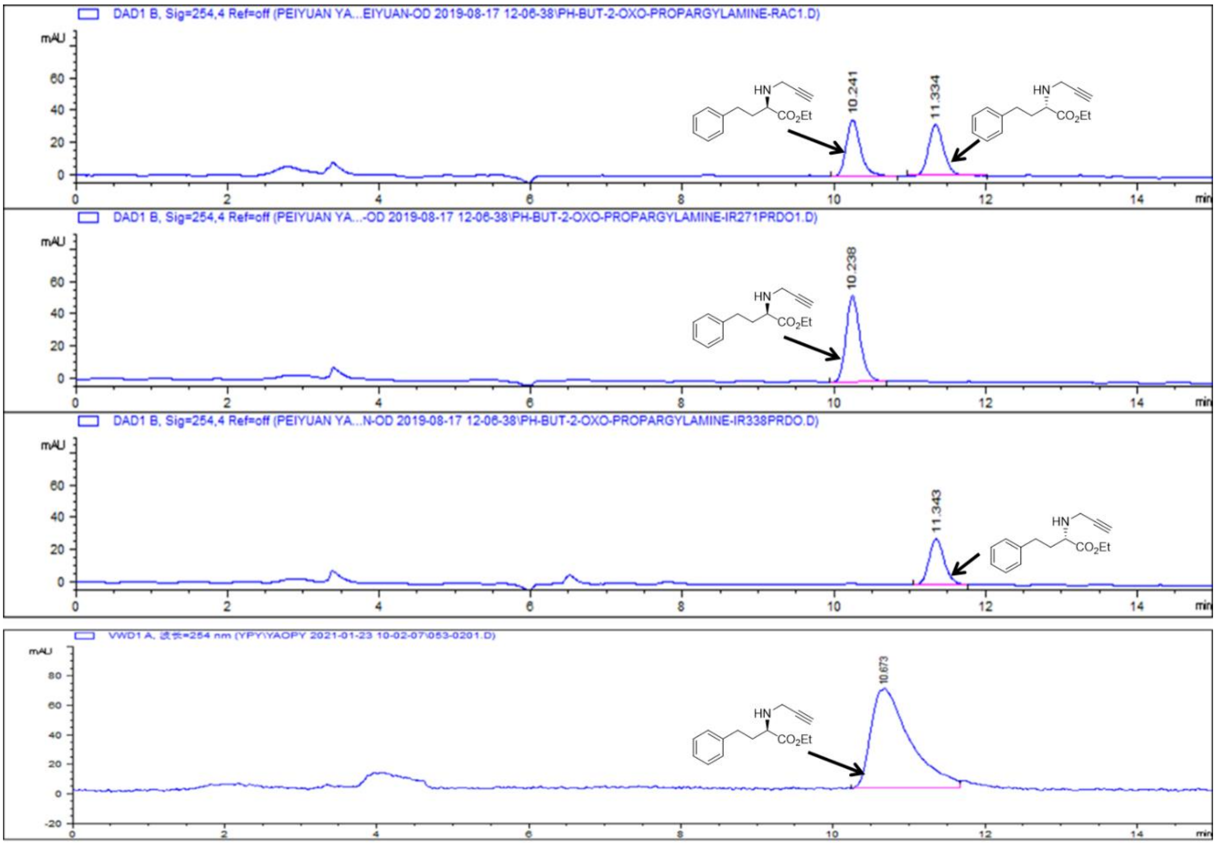

Figure S75. HPLC spectra of racemic ethyl 4-phenyl-2-(methylamino)butyrate (**1b**), products of IR271 ((*R*)-**1b**) and IR338 ((*S*)-**1b**), HPLC condition: CHIRALPAK® IC column, n-hexane /2-propanol (0.1% diethylamine) = 95:5, flow rate = 1.0 mL/min, 254 nm UV detector, tR = 8.47 min (*R*) and tR = 8.85 min (*S*), column temperature = 25°C.

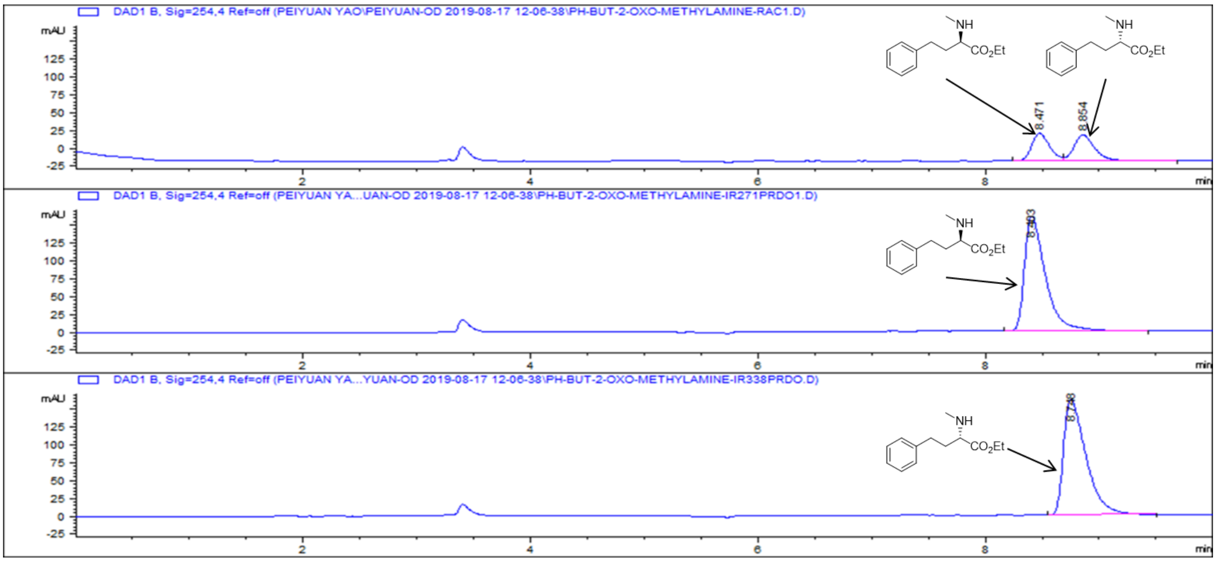

Figure S76. GC spectra of ethyl 4-phenyl-2-oxobutyrate(**1**), ethyl 2-hydroxy-4-phenylbutyrate, crude products of IR271 ((*R*)-**1c**) and IR338 ((*S*)-**1c**).

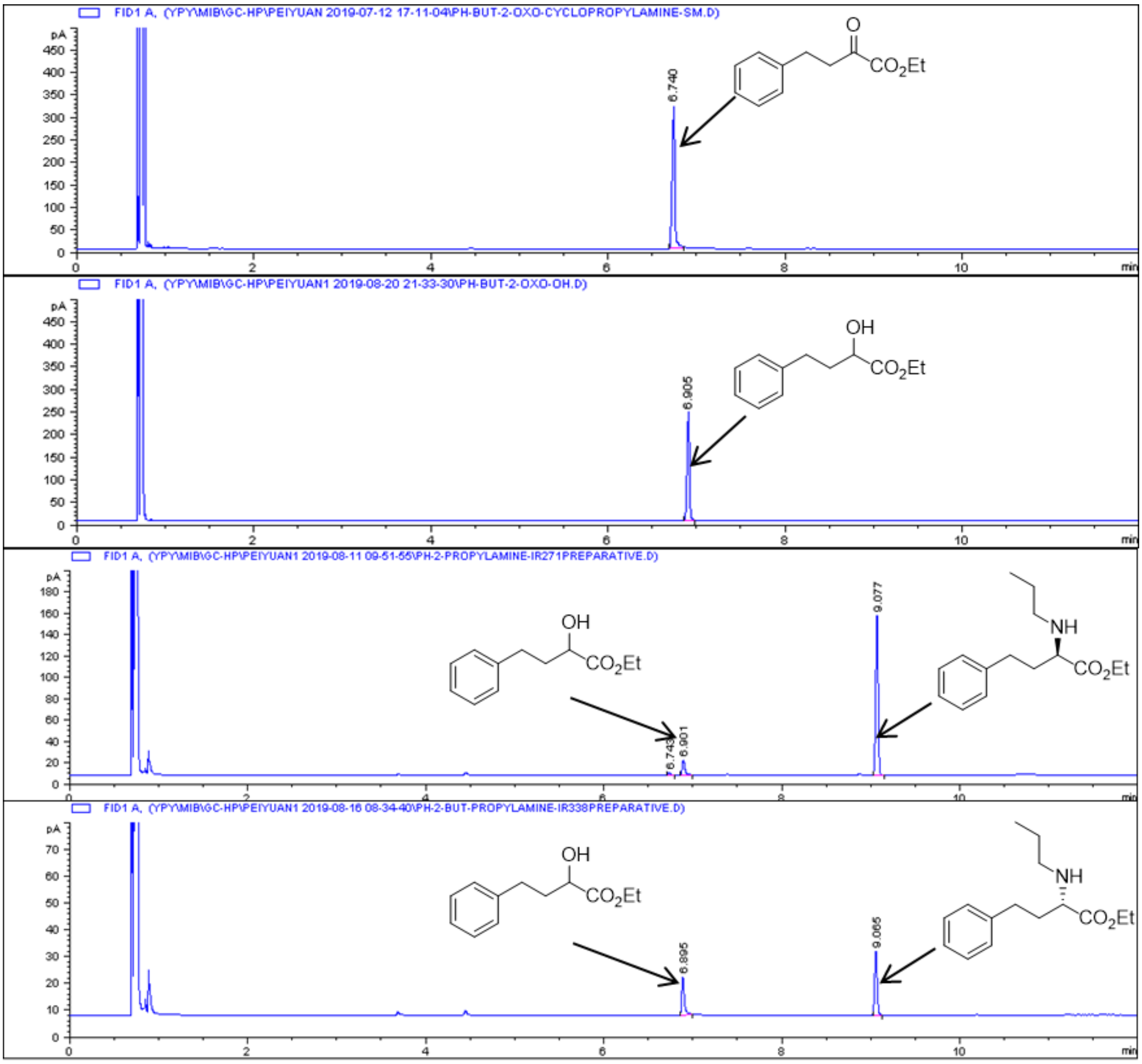

Figure S77. HPLC spectra of racemic ethyl 4-phenyl-2-(propylamino)butyrate (**1c**), products of IR271 ((*R*)-**1c**) and IR338 ((*S*)-**1c**), HPLC condition: CHIRALPAK® AD-H column, n-hexane /2-propanol (0.1% diethylamine) = 97:3, flow rate = 1.0 mL/min, 254 nm UV detector, tR = 5.12 min (*R*) and tR = 5.47 min (*S*), column temperature = 25°C.

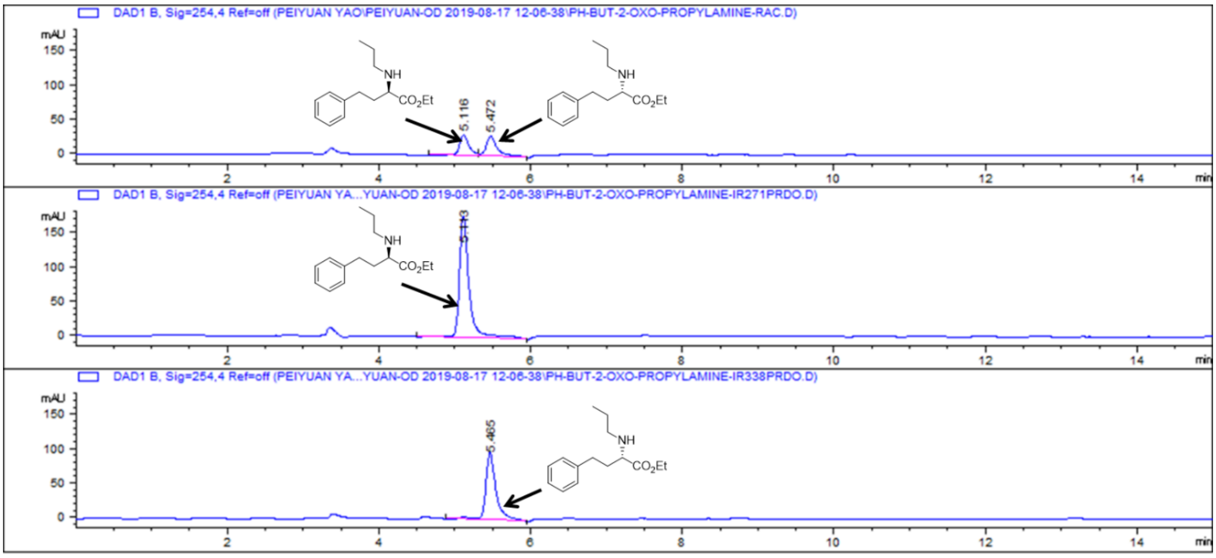

Figure S78. GC spectra of crude products of IR271 ((*R*)-**1d**) and IR338 ((*S*)-**1d**).

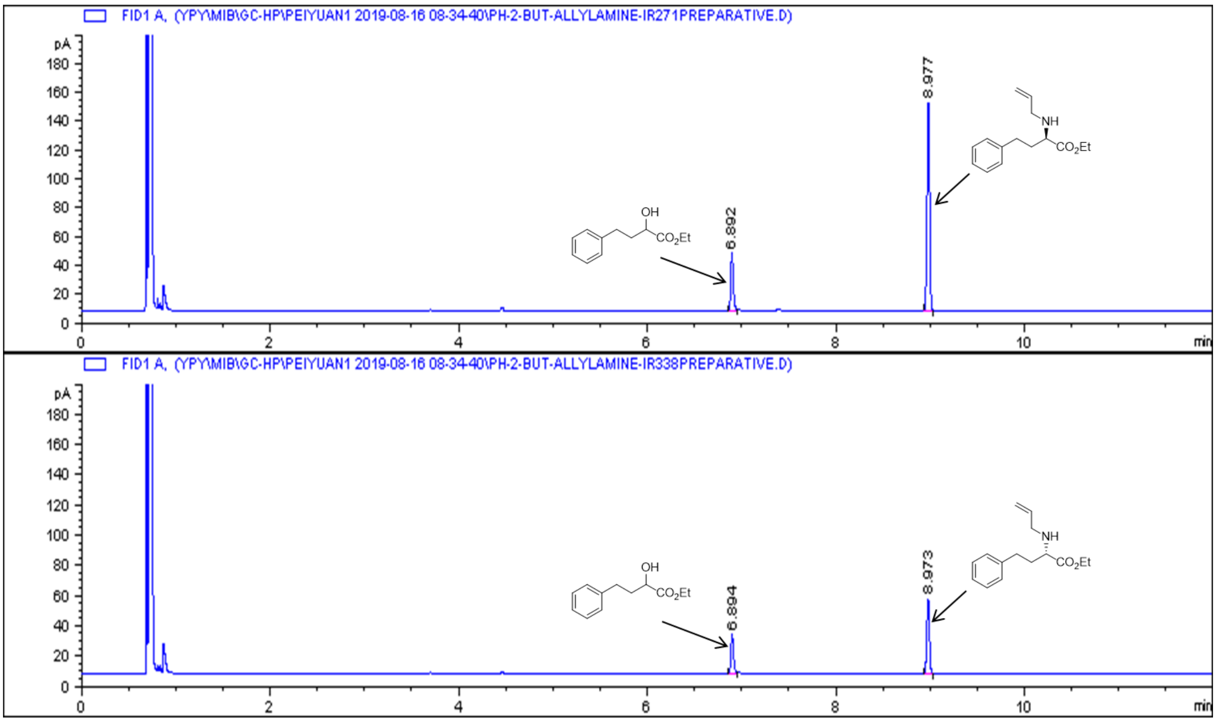

Figure S79. HPLC spectra of racemic ethyl -4-phenyl-2-(allylamino)butyrate (**1d**), products of IR271 ((*R*)-**1d**) and IR338 ((*S*)-**1d**), HPLC condition: CHIRALPAK® IC column, n-hexane /2-propanol (0.1% diethylamine) = 95:5, flow rate = 1.0 mL/min, 254 nm UV detector, tR = 5.24 min (*R*) and tR = 5.63 min (*S*), column temperature = 25°C.

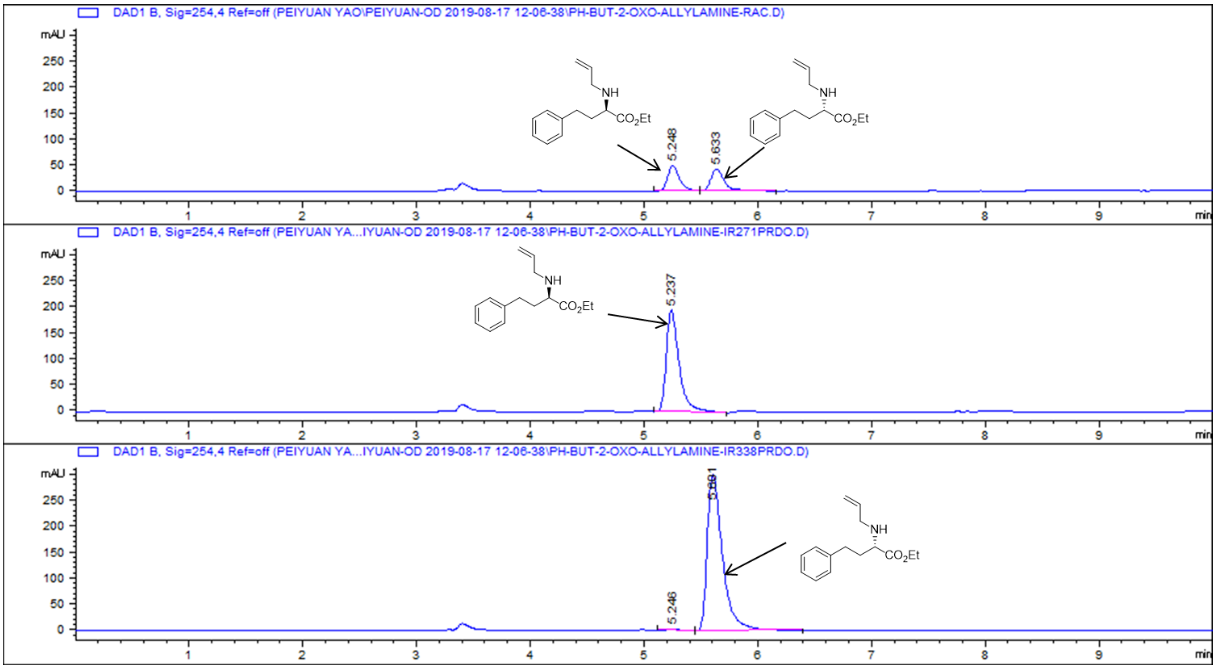

Figure S80. HPLC spectra of racemic ethyl 4-phenyl-2-(cyclopropylamino)butyrate (**1e**), products of IR271 ((*R*)-**1e**) and IR338 ((*S*)-**1e**), HPLC condition: CHIRALPAK® AD-H column, n-hexane /2-propanol (0.1% diethylamine) = 97:3, flow rate = 1.0 mL/min, 254 nm UV detector, tR = 4.97 min (*R*) and tR = 5.21 min (*S*), column temperature = 25°C.

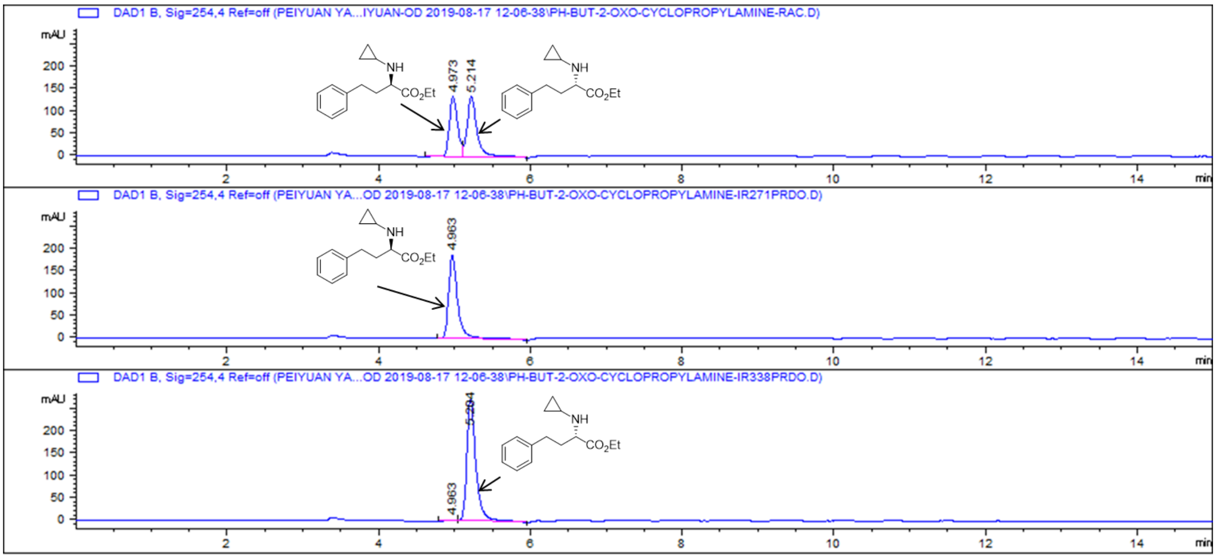

Figure S81. GC spectra of ethyl 4-phenyl-2-oxobutyrate(**1**), ethyl 2-hydroxy-4-phenylbutyrate, standard of ethyl (*R*)-4-phenyl-2-(*N*-(4-methylbenzyl)amino)butyrate ((*R*)-**1g**), analytical reaction product of IR23 ((*R*)-**1g**).

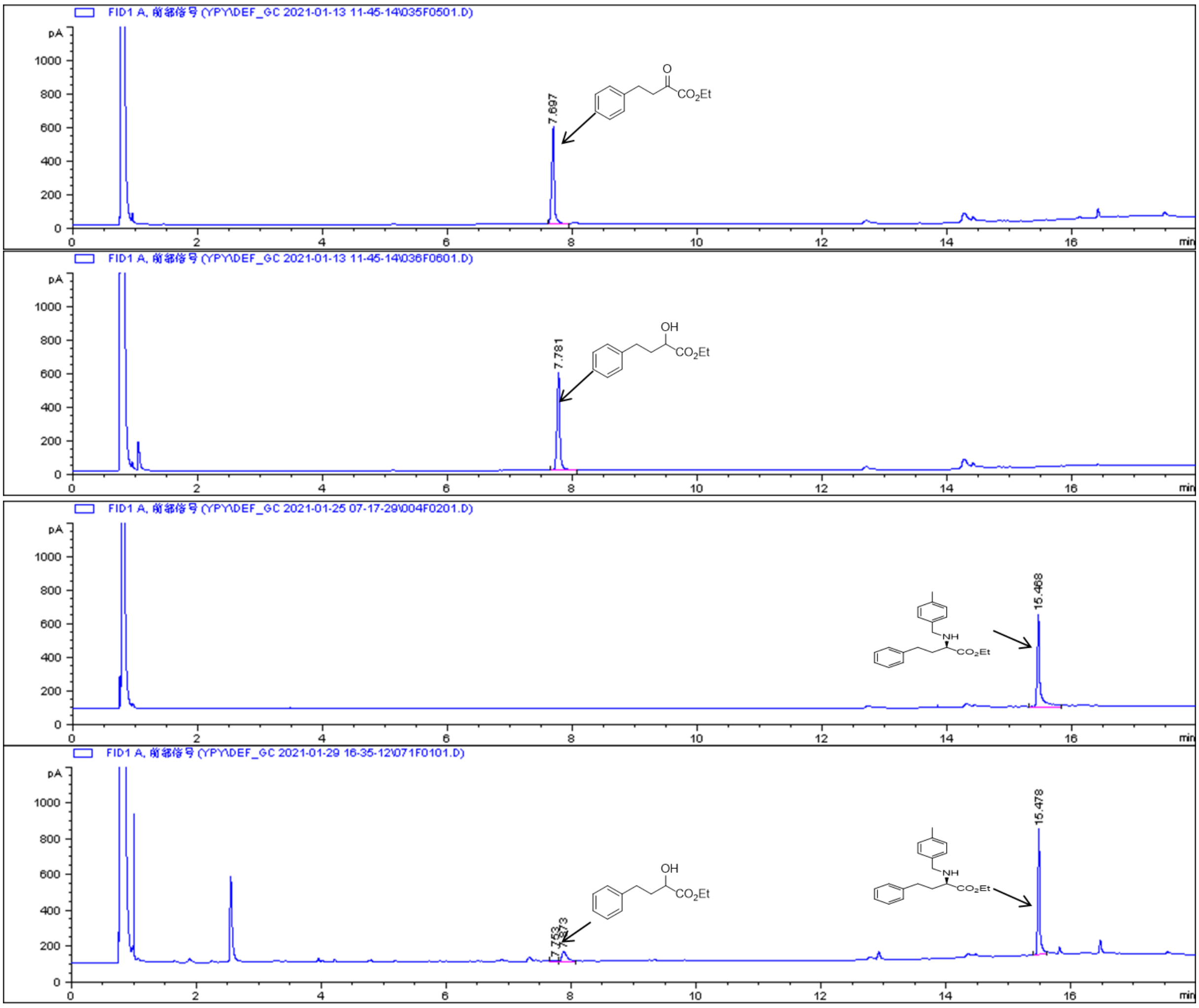

Figure S82. HPLC spectra of racemic ethyl 4-phenyl-2-(*N*-(4-methylbenzyl)amino)butyrate (**1g**), standard of (*R*)-**1g**, product of IR23 ((*R*)-**1g**), HPLC condition: CHIRALPAK® AD-H column, n-hexane /2-propanol (0.1% diethylamine) = 97:3, flow rate = 1.0 mL/min, 254 nm UV detector, tR = 9.34 min (*R*) and tR = 8.36 min (*S*), column temperature = 30°C.

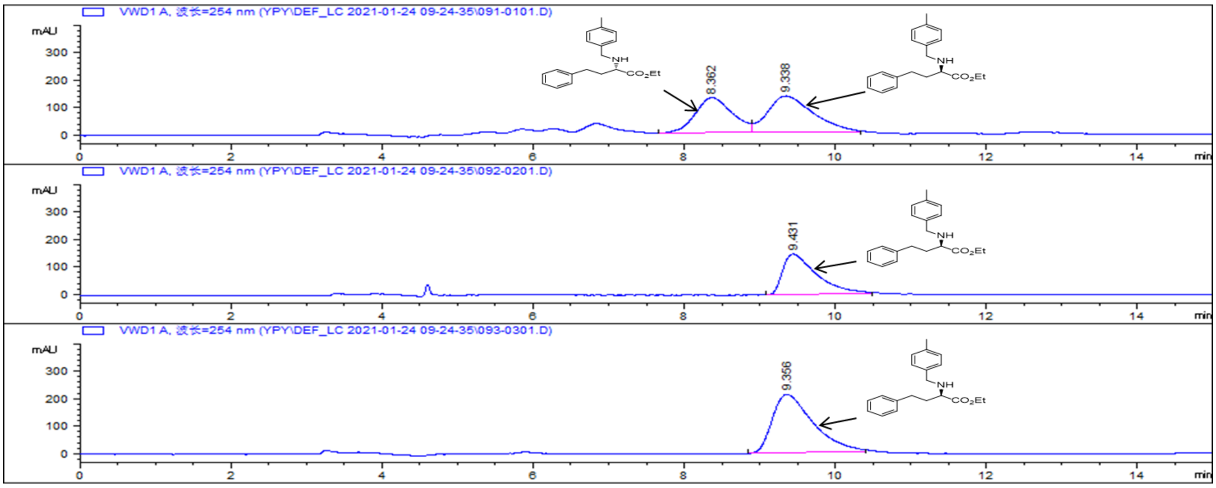

Figure S83. GC spectra of ethyl 2-hydroxy-3-phenylpropionate, crude products of IR271 ((*R*)-**2a**) and IR355 ((*S*)-**2a**).

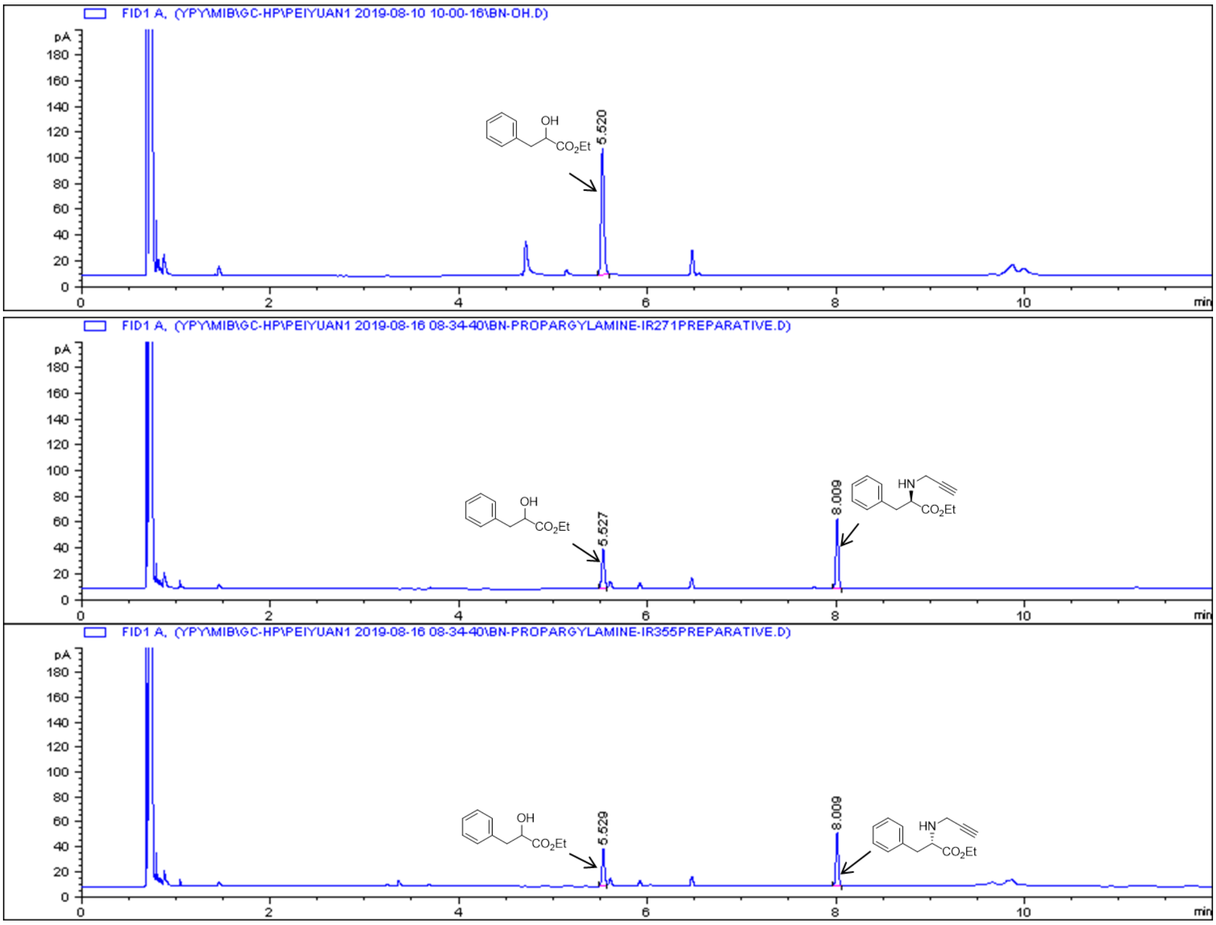

Figure S84. HPLC spectra of standard of ethyl (*R*)-3-phenyl-2-(propargylamino) propionate ((*R*)-**2a**), products of IR271 ((*R*)-**2a**) and IR355 ((*S*)-**2a**), HPLC condition: CHIRALPAK® AD-H column, n-hexane /2-propanol (0.1% diethylamine) = 97:3, flow rate = 1.0 mL/min, 254 nm UV detector, tR = 10.86 min (*R*) and tR = 11.81 min (*S*), column temperature = 25°C.

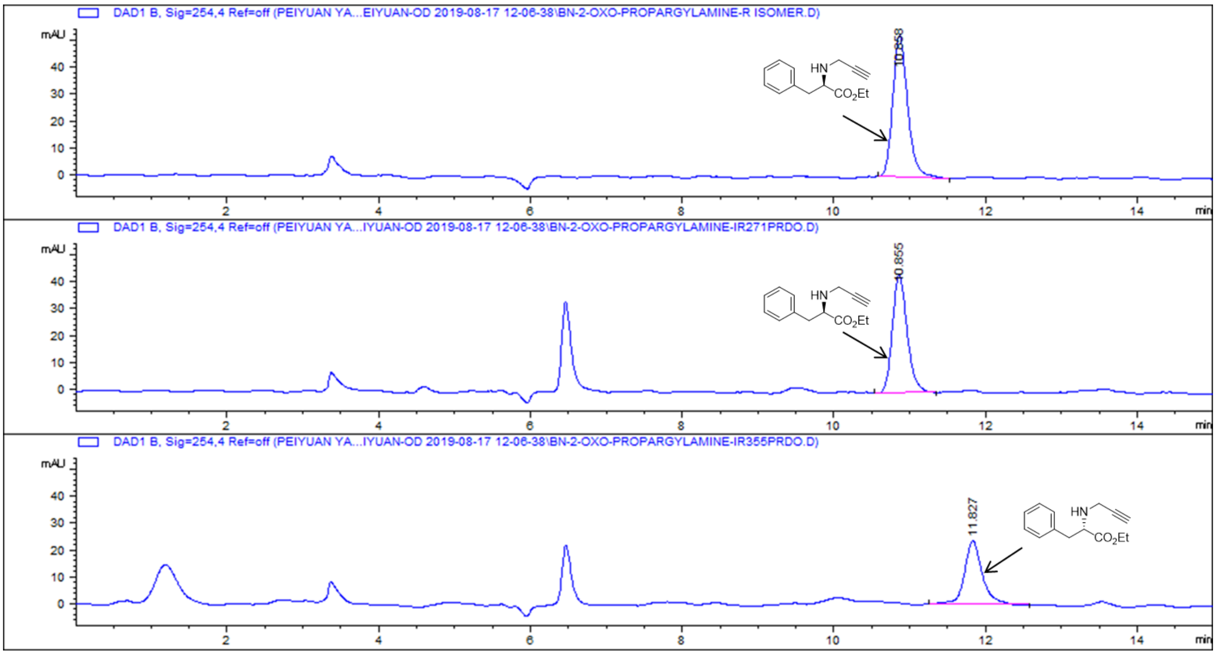

Figure S85. GC spectra of ethyl pyruvate (**3**), analytical reaction product of IR355 ethyl 2-(propargylamino)propionate (**3a**), products of IR23 ((*R*)-**3a**) and IR125 ((*S*)-**3a**), standards of (*S*)-**3a** after acetylization by acetic anhydride catalyzed by 4-(*N*,*N*-dimethyl)pyridine.

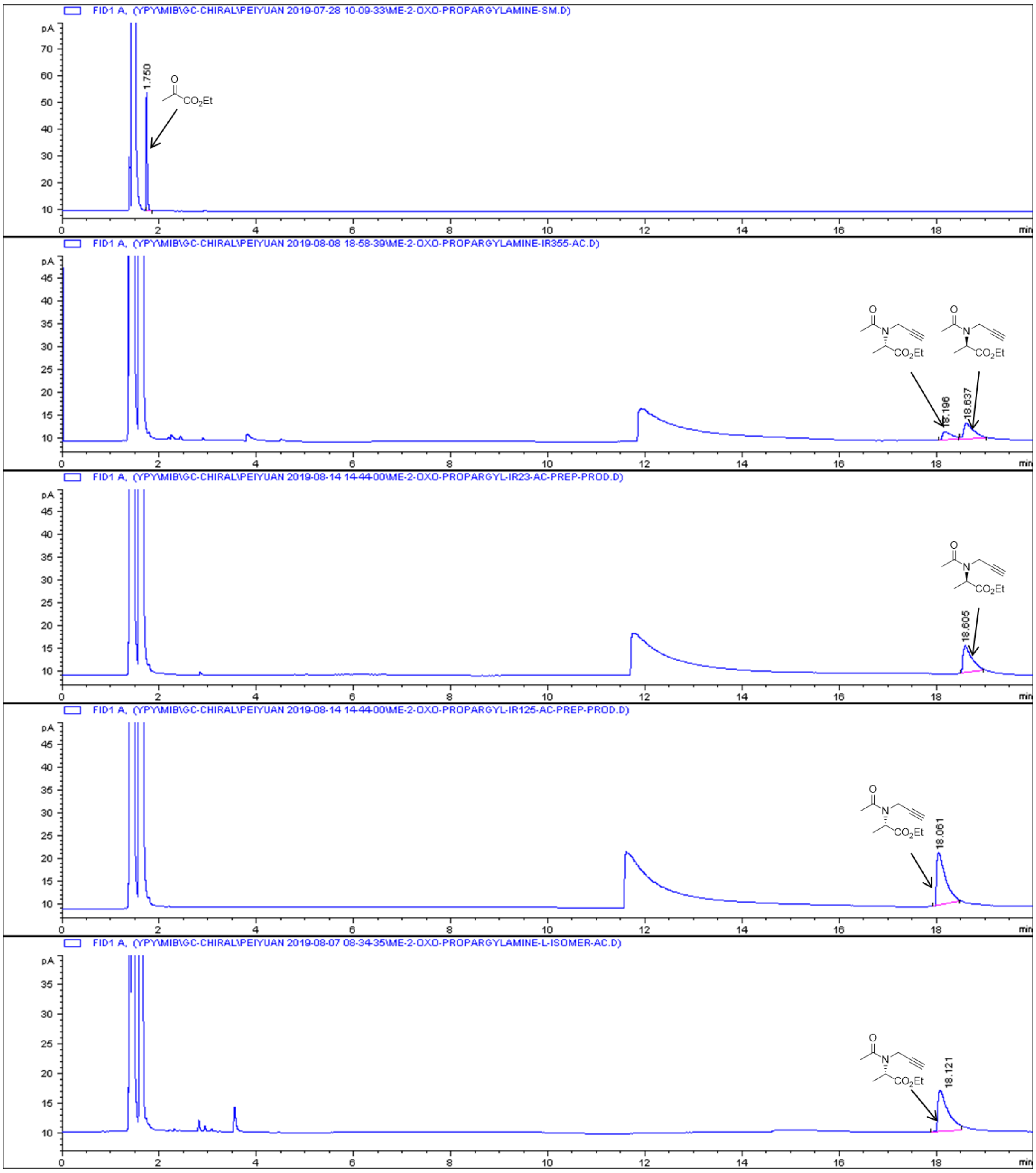

Figure S86. GC spectra of ethyl 2-oxopentanoate (**4**), ethyl 2-hydroxypentanoate, analytical reaction product of IR355 ethyl 2-(propargylamino)valerate (**4a**), products of IR271 ((*R*)-**4a**) and IR358 ((*S*)-**4a**), standard of (*R*)-**4a**.

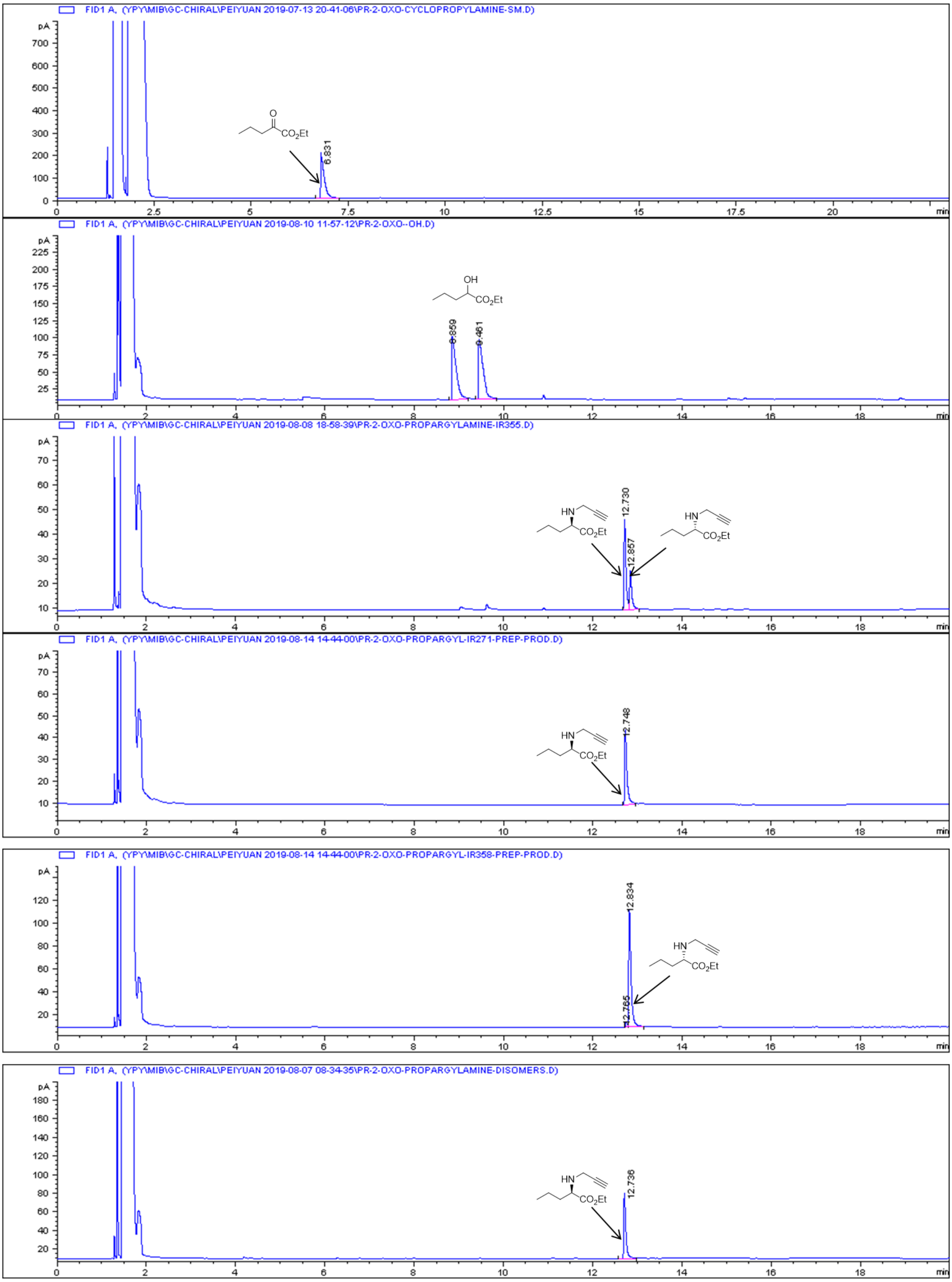

Figure S87. GC spectra of ethyl 4-methyl-2-oxopentanoate (**5**), ethyl 2-hydroxy-4-methylpentanoate, analytical reaction product of IR125 ethyl 4-methyl-2-(propargylamino) valerate (**5a**), products of IR271 ((*R*)-**5a**) and IR358 ((*S*)-**5a**), standard of (*S*)-**5a**.

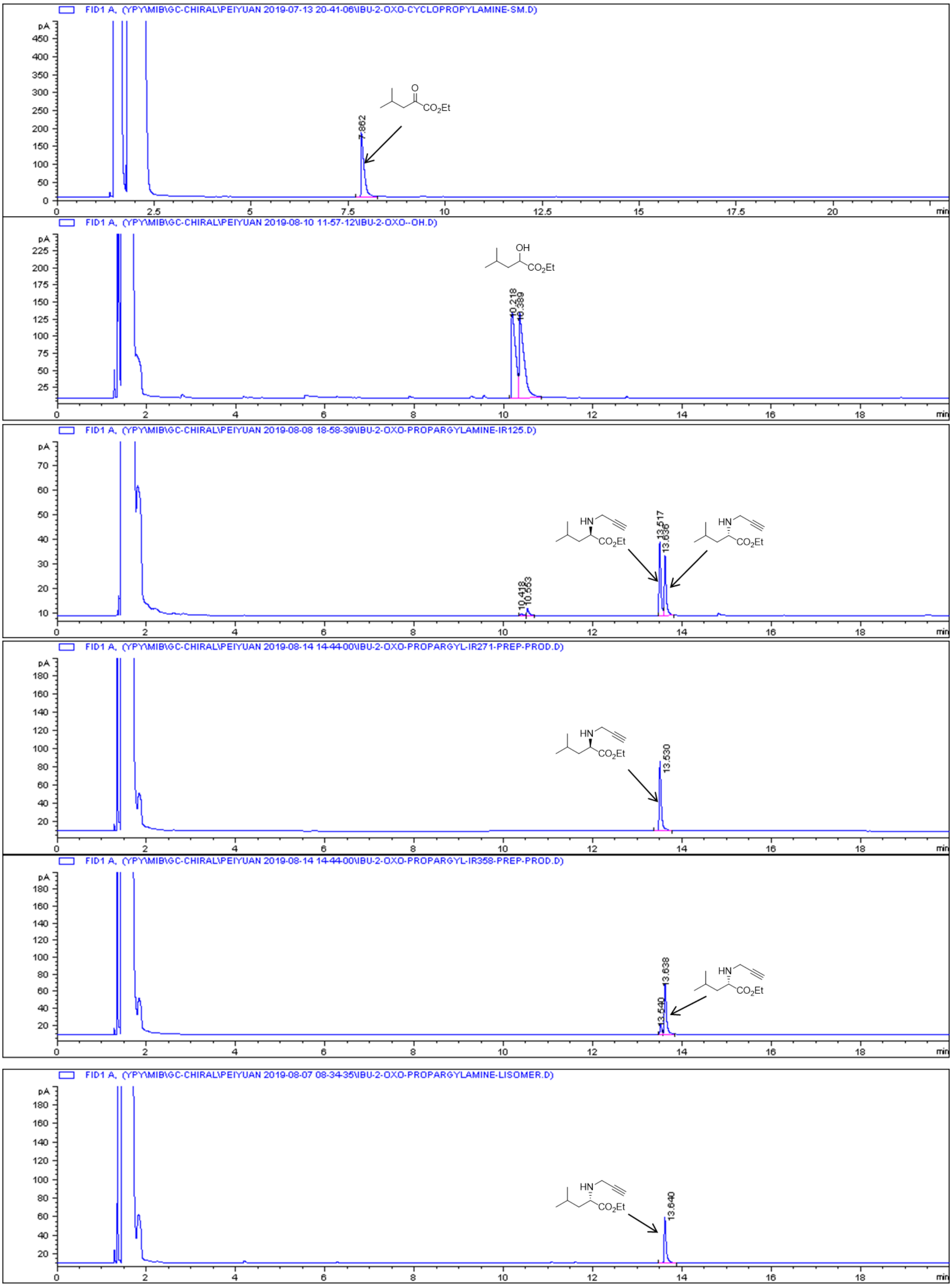

Figure S88. GC spectra of ethyl 2-oxoheptanoate (**6**), ethyl 2-hydroxyheptanoate, analytical reaction product of IR338 ethyl (propargylamino)heptanoate, products of IR271 ((*R*)-**6a**) and IR355 ((*S*)-**6a**).

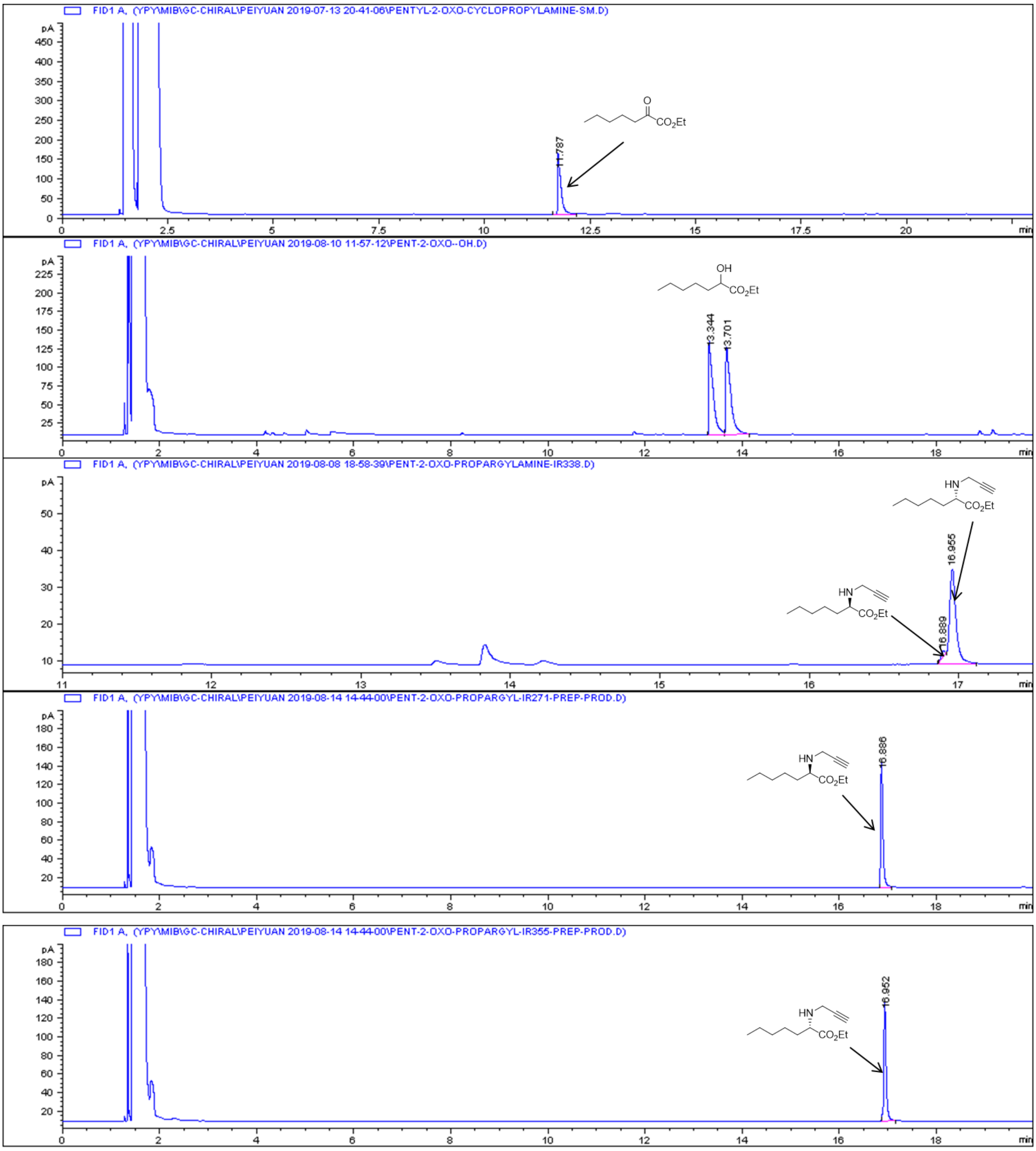

Figure S89. GC spectra of ethyl 4-(2-fluorophenyl)-2-oxobutyrate(**7**), ethyl 4-(2-fluorophenyl)-2-hydroxybutyrate, crude products of IR271 ((*R*)-**7a**) and IR338 ((*S*)-**7a**).

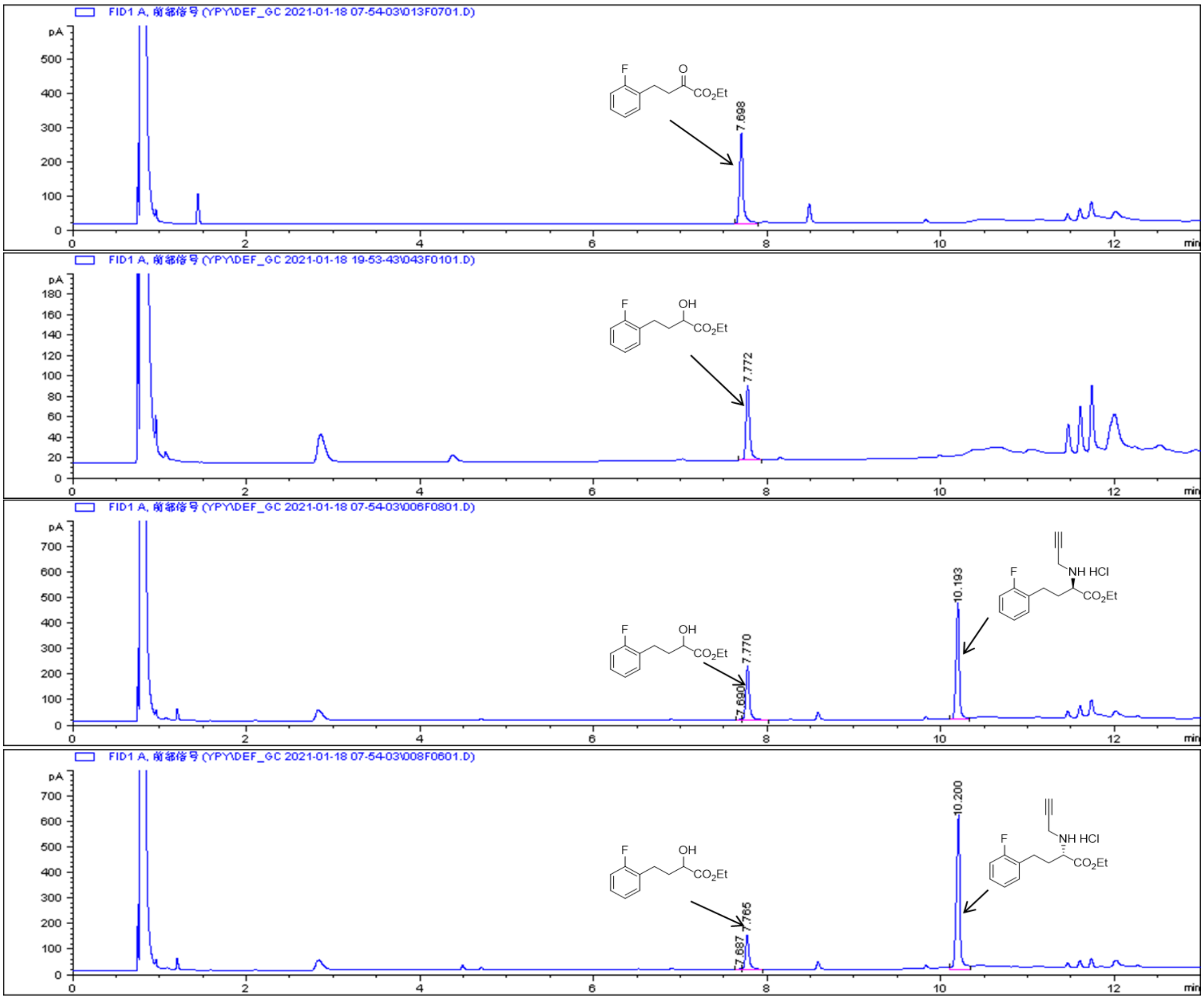

Figure S90. HPLC spectra of racemic ethyl 4-(2-fluorophenyl)-2-(propargylamino) butyrate (**7a**), products of IR271 ((*R*)-**7a**) and IR338 ((*S*)-**7a**), HPLC condition: CHIRALPAK® IC column, n-hexane /2-propanol (0.5% triethylamine) = 95:5, flow rate = 1.0 mL/min, 254 nm UV detector, tR = min (*R*) and tR = min (*S*), column temperature = 30°C.

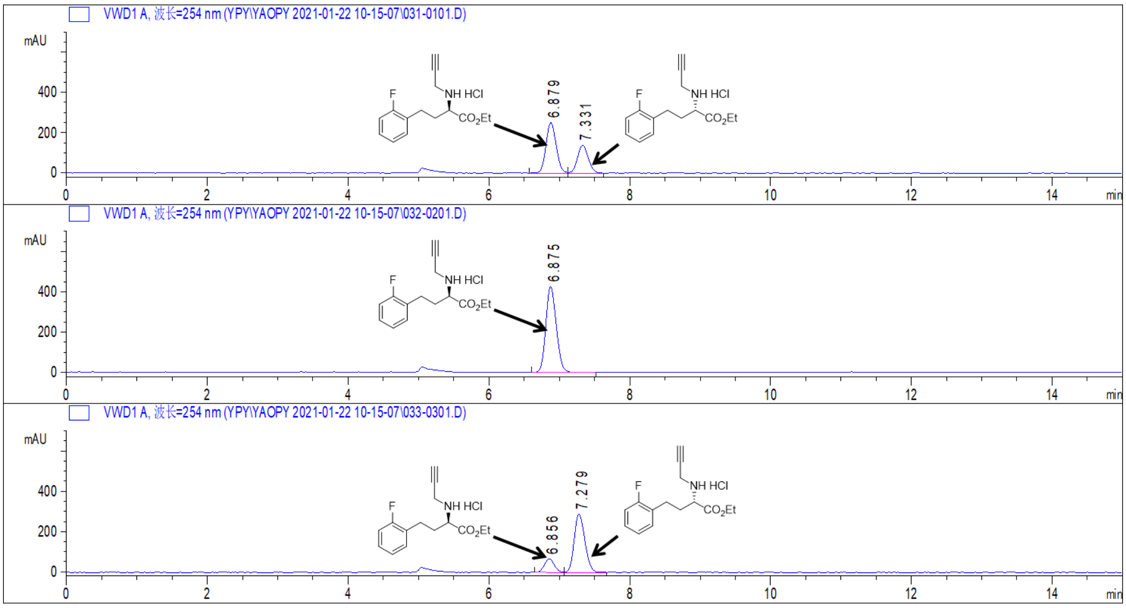

Figure S91. GC spectra of ethyl 4-(3-fluorophenyl)-2-oxobutyrate(**8**), ethyl 4-(3-fluorophenyl)-2-hydroxybutyrate, crude products of IR271 ((*R*)-**8a**) and IR338 ((*S*)-**8a**).

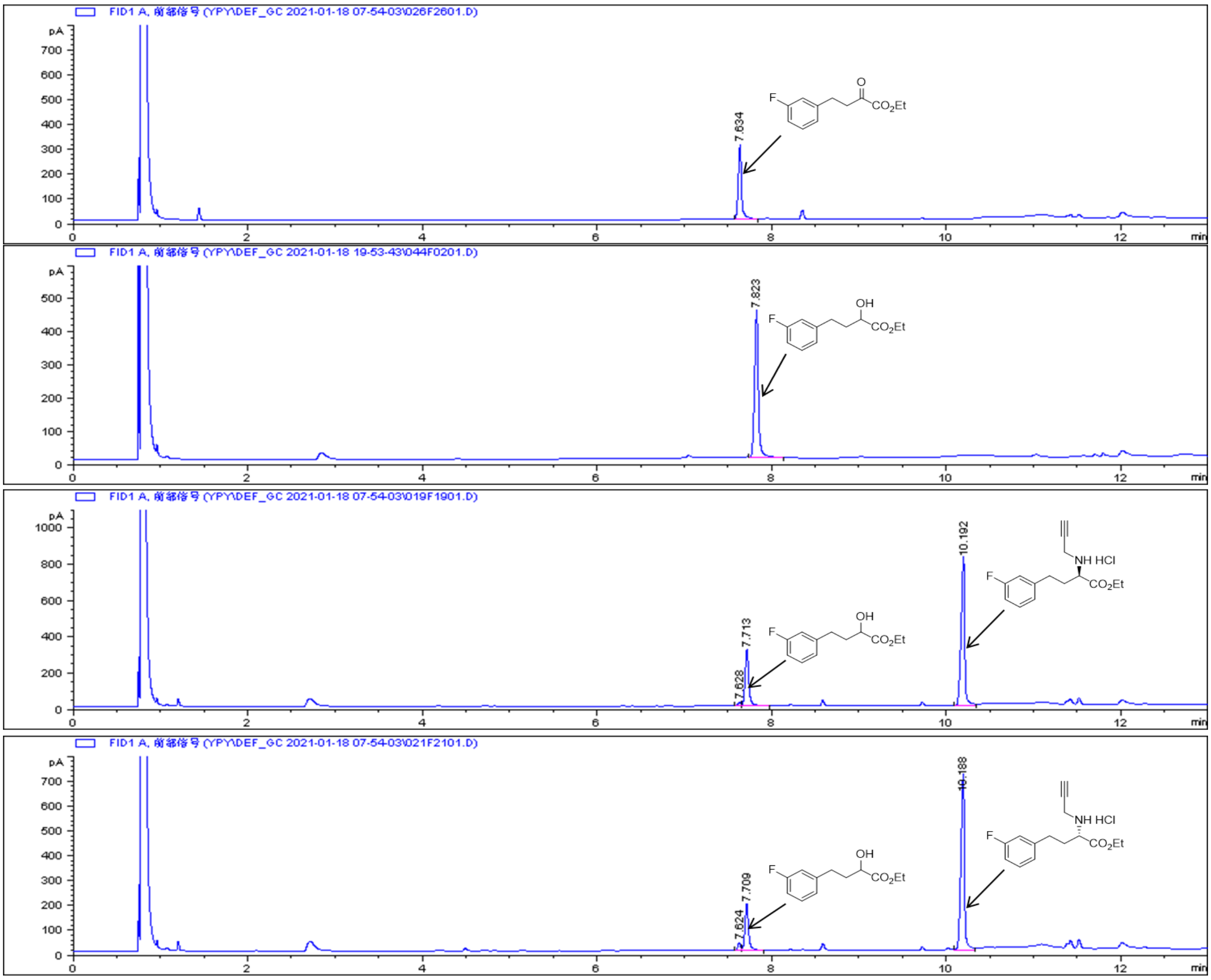

Figure S92. HPLC spectra of racemic ethyl 4-(3-fluorophenyl)-2-(propargylamino) butyrate (**8a**), products of IR271 ((*R*)-**8a**) and IR338 ((*S*)-**8a**), HPLC condition: CHIRALPAK® IC column, n-hexane /2-propanol (0.5% triethylamine) = 95:5, flow rate = 1.0 mL/min, 254 nm UV detector, tR =   min (*R*) and tR =   min (*S*), column temperature = 30°C.

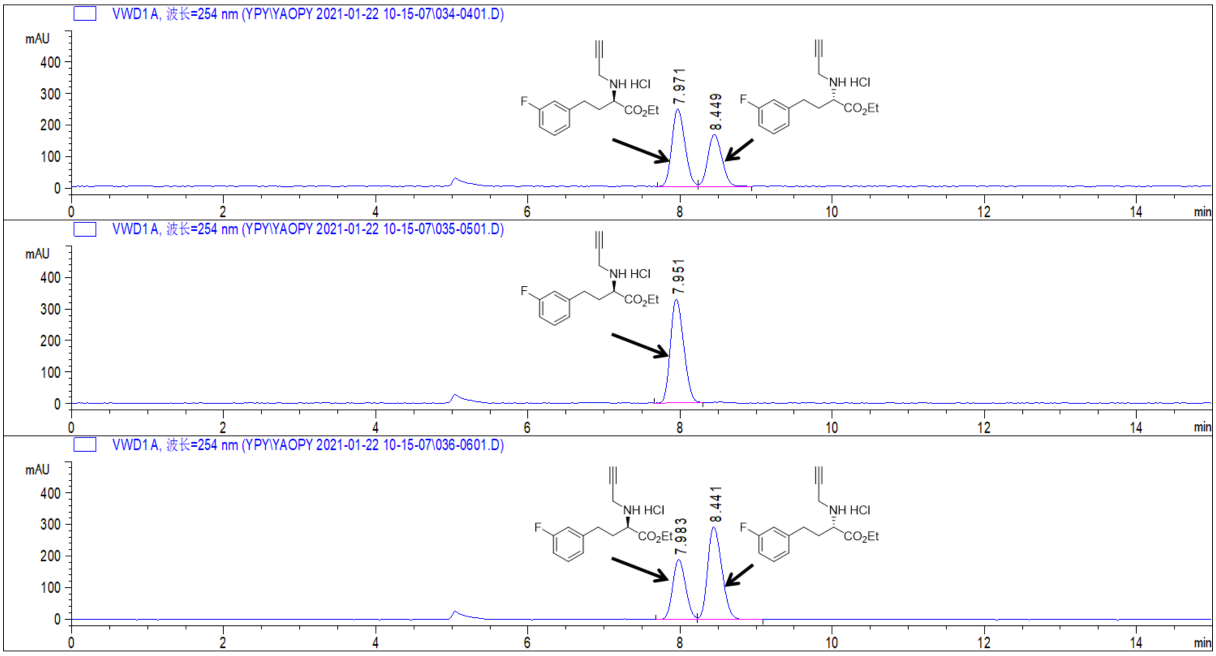

Figure S93. GC spectra of ethyl 4-(4-fluorophenyl)-2-oxobutyrate(**9**), ethyl 4-(4-fluorophenyl)-2-hydroxybutyrate, crude products of IR271 ((*R*)-**9a**) and IR338 ((*S*)-**9a**).

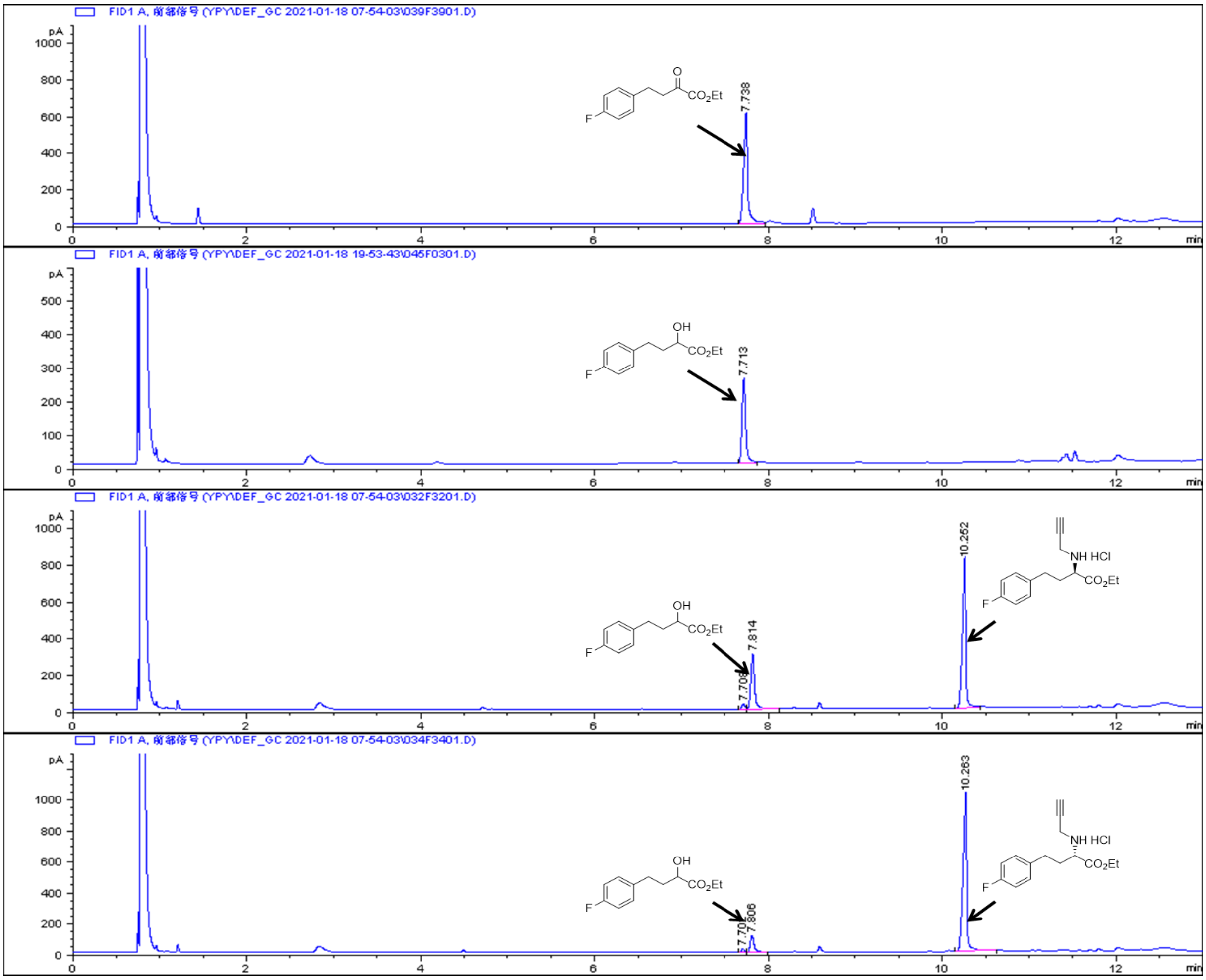

Figure S94. HPLC spectra of racemic ethyl 4-(4-fluorophenyl)-2-(propargylamino) butyrate (**9a**), products of IR271 ((*R*)-**9a**) and IR338 ((*S*)-**9a**), HPLC condition: CHIRALPAK® IC column, n-hexane /2-propanol (0.5% triethylamine) = 95:5, flow rate = 1.0 mL/min, 254 nm UV detector, tR = min (*R*) and tR = min (*S*), column temperature = 30°C.

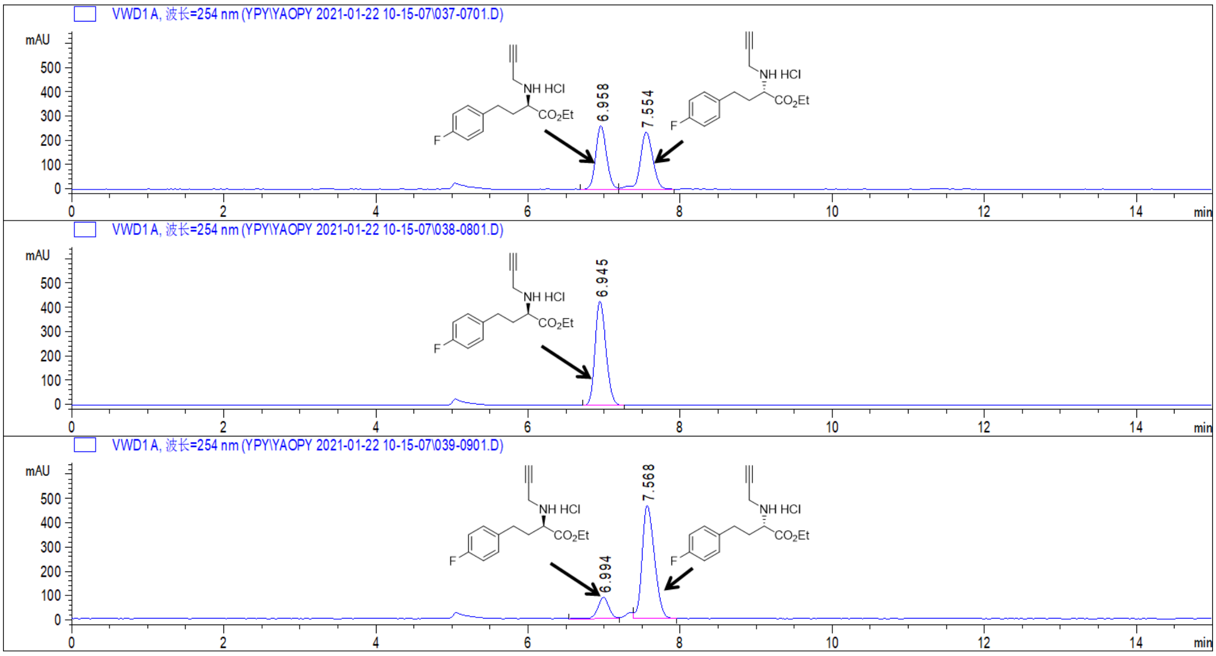

Figure S95. GC spectra of ethyl 2-oxo-2-phenylacetate(**10**), ethyl 2-hydroxy-2-phenylacetate, standard of ethyl (*S*)-2-phenyl-2-(propargylamino)acetate ((*S*)-**10a**) and analytical reaction of IR23.

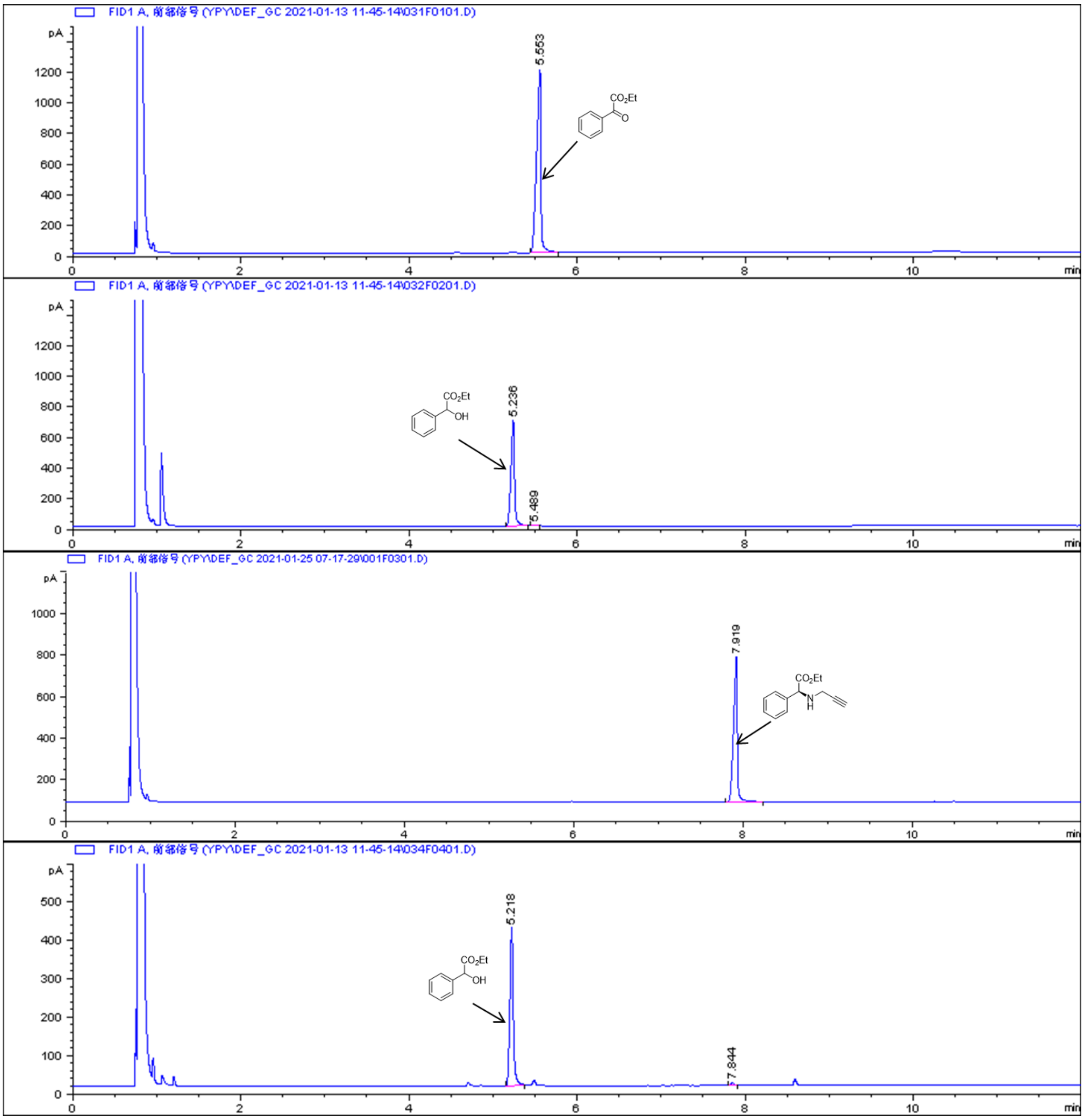

3.6. Time Course Study for the preparation of 1a.

Figure S96. A time course study for the preparation of (*R*)-**1a** catalysed by pIR-271. With time (h) (x-axis) and % conversion (y-axis), monitored by GC-FID.

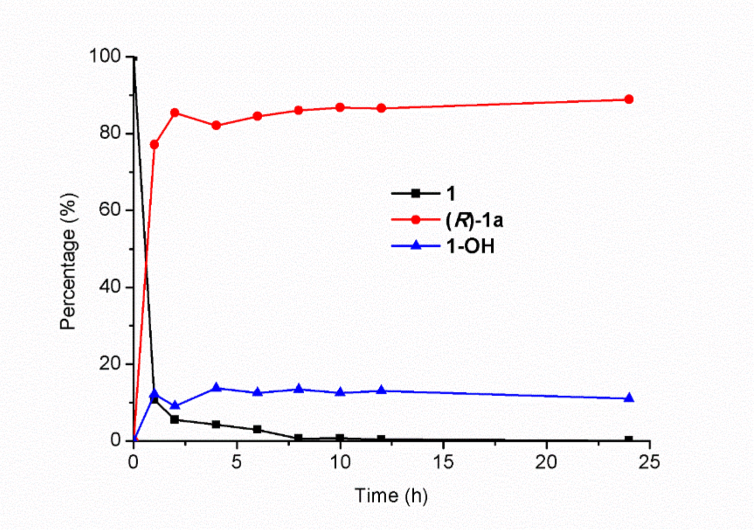

Figure S97. A time course study for the preparation of (*S*)-**1a** catalysed by pIR-338. With time (h) (x-axis) and % conversion (y-axis), monitored by GC-FID.

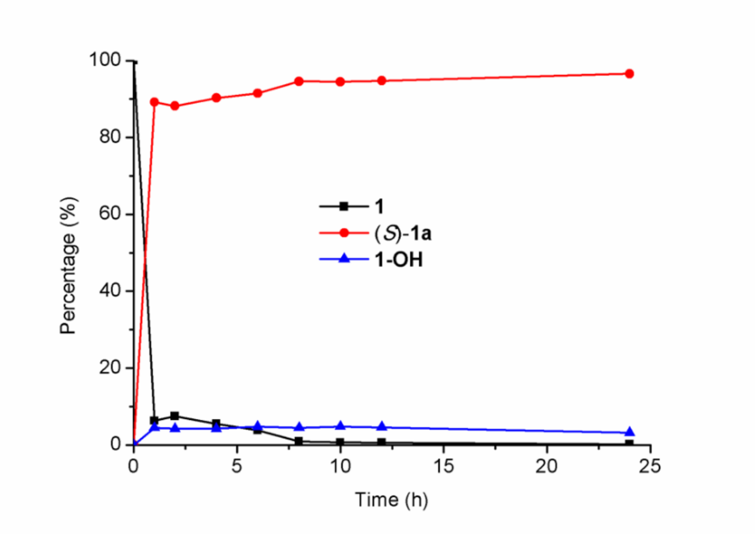

3.7. Investigation of background ketoreductase activity.

Table S5. Investigation of background ketoreductase activity with purified pIR-338 in the preparation of 1a under standard conditions[a], where various controls were employed. Conversion was monitored by GC-FID. *Bs*GDH, glucose dehydrogenase from *Bacillus subtilis*.[5]

Transformation No.	Major Parameter Change	Specific Condition Alterations	% of ethyl 2-oxo-4-phenylbutyrate (1)	% of ethyl 2-hydroxy-4-phenylbutyrate	% of 1a
**1**	Varied IRED concentration	[0.05 mg/mL] IRED	<1	43	**56**
**2**	Varied IRED concentration	[0.1 mg/mL] IRED	0	0	**>99**
**3**	Varied IRED concentration	[0.2 mg/mL] IRED	0	0	**>99**
**4**	Varied IRED concentration	[0.3 mg/mL] IRED	0	0	**>99**
**5**	Varied IRED concentration	[0.4 mg/mL] IRED	0	0	**>99**
**6**	Varied IRED concentration, Higher GDH concentration	[0.25 mg/mL] IRED, [12.5 U/mL] CDX-901	32	21	**41**
**7**	Varied IRED concentration, Higher GDH concentration	[0.5 mg/mL]IRED, [12.5 U/mL] CDX-901	15	24	**61**
**8**	Varied IRED concentration, Higher GDH concentration	[0.75 mg/mL] IRED, [12.5 U/mL] CDX-901	21	22	**57**
**9**	Varied IRED concentration, Higher GDH concentration	[1 mg/mL] IRED, [12.5 U/mL] CDX-901	10	20	**70**
**10**	Varied IRED concentration, Higher GDH concentration	[2 mg/mL] IRED,[12.5 U/mL] CDX-901	13	17	**70**
**11**	Varied IRED concentration, Higher GDH concentration	[3 mg/mL] IRED, [12.5 U/mL]CDX-901	21	12	**67**
**12**	No IRED, only CDX-901	No IRED, only CDX-901 [12.5 U/mL]	25	75	**0**
**13**	Excess amine eqv	[10 mM] ketone, 10 eqv amine	<1	37	**62**
**14**	Stoichiometric NADPH	No GDH, IRED lysate [4 mg/m]L, [50 mM] NADPH	0	0	**>99**
**15**	Stoichiometric NADPH	No GDH, IRED purified [0.5 mg/mL], [50 mM] NADPH	0	0	**>99**
**16**	Varied GDH concentration, high IRED concentration	[5 U/mL]] CDX-901, [0.5 mg/mL] IRED	0	0	**>99**
**17**	Varied GDH concentration, high IRED concentration	[2.5 mg/mL] CDX-901, [0.5 mg/mL] IRED	0	0	**>99**
**18**	BsGDH	BsGDH Lysate [0.5 mg/mL]	14	43	**43**
**19**	BsGDH	BsGDH Pure [0.5 mg/mL]	0	0	**>99**

[a] Reaction conditions: 50 mM ethyl 2-oxo-4-phenylbutyrate (**1**), propargylamine (**a**, 100 mM), 0.2 mg/mL IRED purified, 5 U/mL CDX-901 GDH, 0.4 mM $NADP^+$, 62.5 mM glucose, 10% (v/v) DMSO, sodium phosphate buffer (100 mM, pH 7.5), 500 µL reaction volume, 30 °C, 200 rpm, 24 h.

4. References

[1] a) M. Burns, C. A. Martinez, B. Vanderplas, R. Wisdom, S. Yu, R. A. Singer, *Org. Process Res. Dev.* **2017**, *21*, 871-877; b) J. M. Bueno, M. Carda, B. Crespo, A. C. Cuñat, C. de Cozar, M. L. León, J. A. Marco, N. Roda, J. F. Sanz-Cervera, *Bioorg. Med. Chem. Lett.* **2016**, *26*, 3938-3944.

[2] D. Fleury, M. B. Fleury, N. Platzer, *Tetrahedron*, **1981**, *37*, 493-501.

[3] A. F. Abdel-Magid, K. G. Carson, B. D. Harris, C. A. Maryanoff, R. D. Shah, *J. Org. Chem.* **1996**, *61*, 3849-3862.

[4] J. R. Marshall, P. Yao, S. L. Montgomery, J. D. Finnigan, T. W. Thorpe, R. B. Palmer, J. Mangas-Sanchez, R. J. Duncan, R. S. Heath, K. M. Graham, D. J. Cook, S. J. Charnock, N. J. Turner, *Nat. Chem.*, doi:10.1038/s41557-020-00606-w.

[5] G. A. Aleku, S. P. France, H. Man, J. Mangas-Sanchez, S. L. Montgomery, M. Sharma, F. Leipold, S. Hussain, G. Grogan, N. J. Turner, *Nat. Chem.* **2017**, *9*, 961-969.

[6] S. L. Montgomery, A. Pushpanath, R. S. Heath, J. R. Marshall, U. Klemstein, J. L. Galman, D. Woodlock, S. Bisagni, C. J. Taylor, J. Mangas-Sanchez, J. I. Ramsden, B. Dominguez, N. J. Turner, *Sci. Adv.* **2020**, *6*, eaay9320.
